# Long Range Electronic Effects on the Host–Guest Complexation within the Oxygen Depleted 5,5′-Bicalixarene Cavities

**DOI:** 10.1021/acs.joc.3c01566

**Published:** 2023-10-31

**Authors:** Michal Farber, Pintu Maity, Abhishek Baheti, Adina Golombek, Tal Schwartz, Roman Dobrovetsky, Arkadi Vigalok

**Affiliations:** School of Chemistry, The Sackler Faculty of Exact Sciences, Tel Aviv University, Tel Aviv 69978, Israel

## Abstract

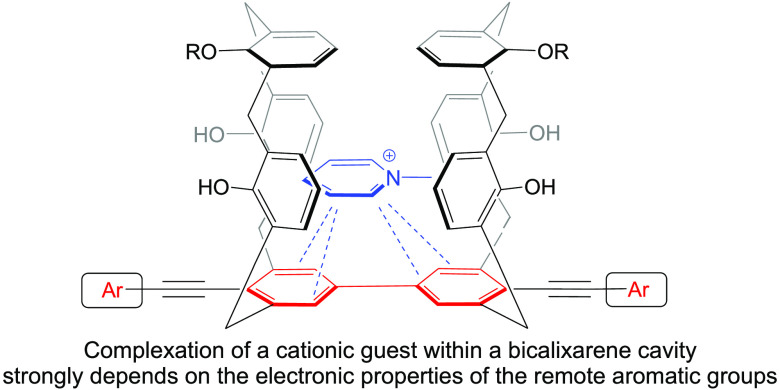

We report the synthesis of a series of the oxygen-depleted conjugated 5,5′-Bicalix[4]arene compounds bearing various substituents at the terminal positions of the conjugated chain and their fluorescence response to the presence of a cationic *N*-methylpyridinium guest. The complexation of this cation within the bicalixarene cavity results in the fluorescence quenching, with the host molecules bearing electron-donating groups demonstrating a stronger fluorescence response. These results show the importance of the electronic effects on the host–guest complexation within the hydrophobic calixarene scaffolds.

For several decades, calixarene compounds have been associated with the supramolecular complexation of small molecules and ions within their hydrophobic aromatic cavities.^1^ Considering the electron-rich nature of a calixarene cavity, most of the common guests bear a formal positive charge.^2^ Interestingly, although numerous studies on the calixarene host–guest complexation have been reported, none of them explored common electronic effects on binding properties. Such a lack of studies can be explained by the structural constraints of the calixarene scaffolds. Thus, the attachment of various groups to the phenolic oxygen atoms of the lower rim only marginally affects the electronic properties of the aromatic cavity. Introduction of the substituents directly to the cavity via the substitution at the upper rim should strongly affect the electronic properties, but also bring steric hindrance and changes the calixarene’s conformation (Figure 1a). The conical geometry of the major conformer of a common calixarene scaffold, such as calix[4]arene (hereafter, calixarene), is another challenge for the host–guest complexation as the aromatic rings are tilted which should decrease π-interactions. Thus, relatively low binding constants have been reported for the majority of the small organic cations within the calixarene cavity, most of them obtained from the ^1^H NMR titration studies performed at high concentrations.^3,4^ Scaffolds containing two calixarene moieties demonstrate stronger guest binding compared with the single calixarene compounds (Figure 1b), likely due to simultaneous participation of both cavities in the guest complexation.^5^ However, the issues with the substituent attachment still remain, and no systematic studies on the effect of the substituent on the host–guest complexation in any of the calixarene scaffolds have been reported, to the best of our knowledge.

**Figure 1:**
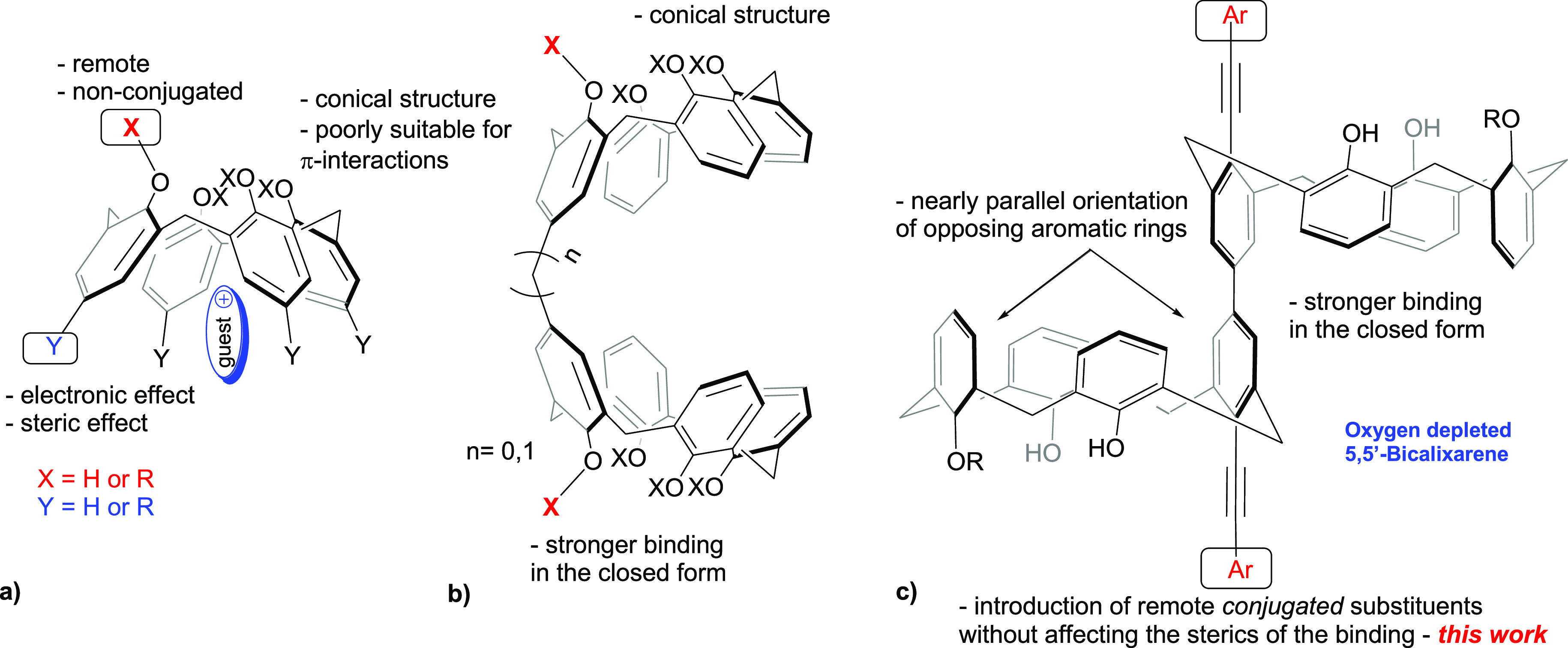
Cation host–guest complexation with selected calix[4]arene scaffolds.

In our search for the responsive fluorescent probes based on host–guest complexation within the calixarene cavities, we developed the synthesis of the conjugated oxygen-depleted 5,5′-Bicalixarene scaffolds (Figure 1c).^6^ These scaffolds demonstrated interesting activity in the fluorescent detection of nitric oxide, in both organic and aqueous solutions.^7^ We hypothesized that they also possess two structural features that make them ideally suitable for the studies of the substituent effect on the host guest complexation: (1) having a conjugated fragment in place of the phenolic oxygen allows direct studies of the electronic effect without changing the steric properties of the cavities and (2) available crystallographic data show that the cavities exist in the pinched cone conformation with the deoxygenated aromatic ring being parallel to the opposite phenolic ring.^6,8^ This parallel arrangement is particularly suitable for the π-interactions with planar guests, such as the *N*-methylpyridinium cation.^9^ Here, we present our studies of the substituent electronic effect on host–guest complexation within the calixarene scaffolds.

Compounds **1a** and **2a** were reported earlier.^7b^ The synthetic procedures toward compounds **1a**–**e**, **2a,b**, and **3a,b** are shown in Scheme 1. Generally, the 5,5′-Bicalixarene scaffold was prepared according to the modified procedure, originally reported by Neri et al.,^10^ with K_2_S_2_O_8_ used as an oxidant in the phenol dimerization step. The Sonogashira cross-coupling reaction at the lower rim followed the improved protocol where the more potent Pd_2_(dba)_3_/*t*-Bu_3_P catalytic system was used in place of the previously described (Ph_3_P)_2_PdCl_2_.^6,11^ While generally the corresponding arylacetylene coupling partner was reacted directly with the bicalixarene ditriflate **6** giving the desired product in a moderate yield, in the case of **1b**, low yields were obtained. Thus, compound **6** was first converted to terminal diacetylene **7**, followed by cross-coupling with 4-iodoanisol. Compounds **1a**–**e** and **2a** were used to establish the electronic effects on host–guest complexation with the bicalixarene scaffold. Compounds **2b** and **3a,b** were prepared to study the potential effect of the conjugation length on the complexation properties. The two weakly donating methyl groups at the 3 and 5 positions in **3a,b** were used to compensate for the higher electronegativity of the sp-hybridized acetylenic carbon atoms. In addition, compound **1f** (Ar = 4-NMe_2_C_6_H_4_) bearing electron-donating −NMe_2_ groups was prepared; however, it was unstable in solution under typical conditions and was not used in further studies. All prepared compounds are white or slightly yellowish solids highly soluble in organic solvents (CH_2_Cl_2_, DMF). In solution, the compounds show strong blue fluorescence when irradiated with a common 365 nm UV lamp. Compounds **2a,b** and **3b** emit at longer wavelengths (ca. 420 nm) due to more extended conjugation. Normalized emission spectra of compounds **1**–**3** are shown in Figure 2.

**Scheme 1:**
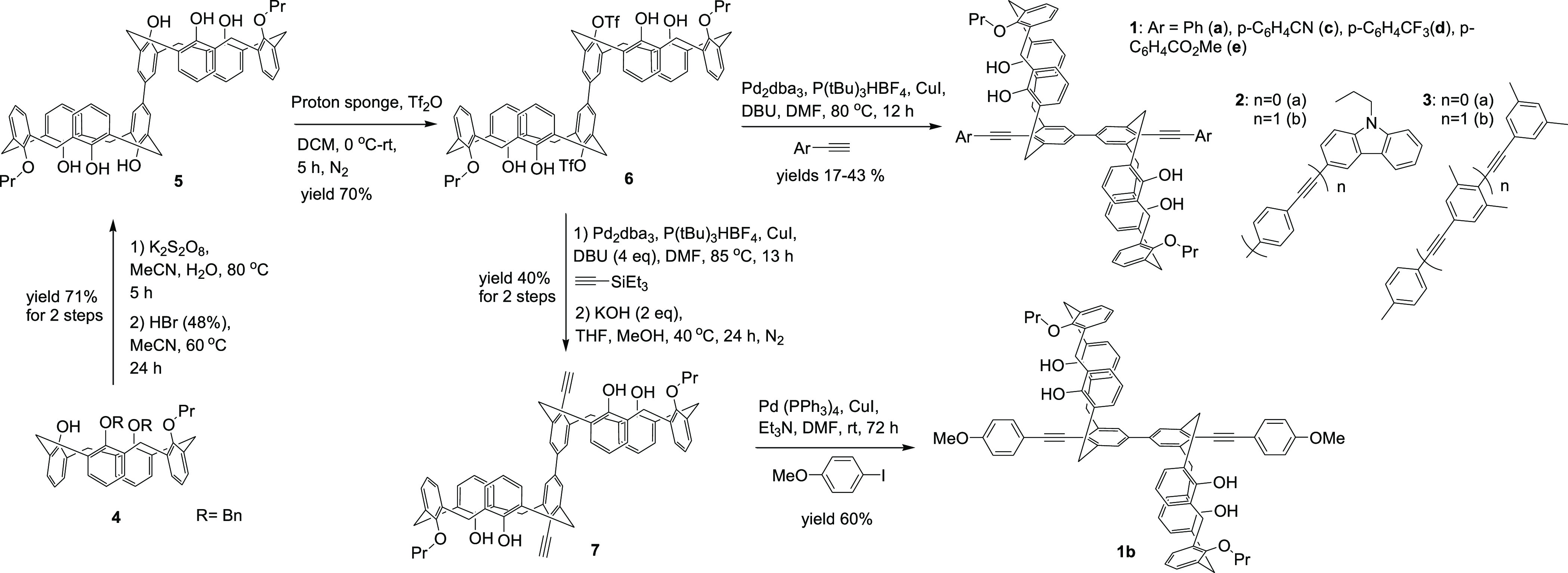
Synthesis of Compounds **1**–**3**

**Figure 2:**
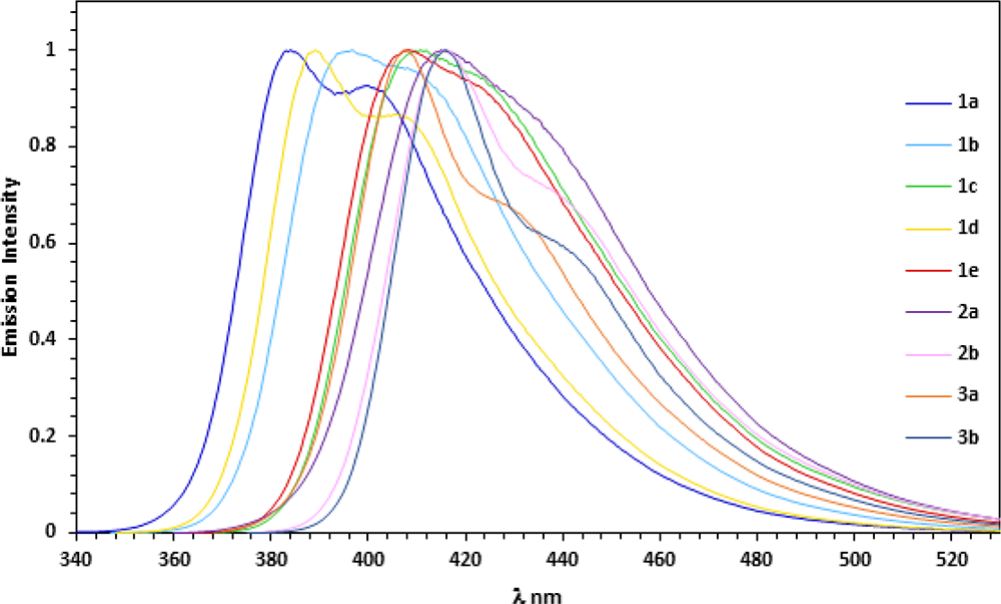
Normalized emission spectra of compounds **1**–**3**.

Compounds **1**–**3** undergo gradual fluorescence quenching in the presence of *N*-methylpyridinium triflate (*N*-MeN^+^C_5_H_5_ OTf^–^, **8**) in a CHCl_3_–CH_3_CN (9:1) solution. Figure 3 shows the fluorescence response of compounds **1**–**3** to different concentrations of **8**. Earlier studies of the fluorescent response of calix[4]arene compounds to the cation of **8** indicated the static quenching process.^12^ To determine the type of quenching operating for the bicalixarenes receptors **1**–**3**, we performed the fluorescence lifetime measurements with a selected conjugated 5,5′-bicalixarene compound. Under the experimental conditions, no changes in the fluorescence decay were observed when **1b**, **1c**, or **3b** was mixed with **8** in CHCl_3_–CH_3_CN (9:1) solution (Figures S50–55), suggesting that static quenching takes place. Thus, the apparent binding constants (*K*_a_) between the bicalixarene cavities and the cation of **8** can be derived directly from the Stern–Volmer plots (Figures S49). The calculated constants are shown in Table 1. The results demonstrate that electron-rich **1b** responds significantly more strongly to **8** than electron-poor **1c**–**e**. Although **1f** demonstrated limited stability, its electron-rich carbazole analogue **2a** was stable and showed the strongest host–guest binding of **8** (Table 1, entry 1). Inserting a conjugated phenylene ethynylene linker between the carbazole fluorophore and bicalixarene receptor (compound **2b**) resulted in a substantial drop in the binding affinity with the constant being similar to the unsubstituted **1a** (Table 1, entry 4). In contrast, extension of the conjugation with one or two arylene ethynylene linkers (compounds **3a,b**) did not significantly affect the binding compared with the parent **1a**. Overall, the *K*_a_’s found here are larger than those obtained by ^1^H NMR spectroscopy for the parent 5,5′-Bicalixarene^10^ and similar hosts containing two calixarene cavities.^4b^ The latter experiments were performed in different solvents and with compounds carrying alkyl groups at the lower rim. For the direct comparison of the *K*_a_’s, we performed the ^1^H NMR titration measurements (CDCl_3_–CD_3_CN, 9:1) between **8** and compounds **1b,c** representing the host with an electron-donating and electron-withdrawing substituent, respectively (Figures S56–S59). The calculated values of (1.0 ± 0.2) × 10^4^ M^–1^ (**1b**) and (0.9 ± 0.1) × 10^3^ M^–1^ (**1c**) are in a good agreement with the fluorescence data obtained at 50-fold lower concentrations (0.01 mM vs 0.5 mM). It must be noted that the effective concentrations (activities) of compounds can deviate from the concentrations commonly used in the equilibrium studies, and even more so when salts (such as **8**) are involved.^13^ Indeed, the complexation of an *N*-alkylpyridinium cation within a calixarene cavity shows considerable dependence on the nature of the counterion.^3,14^ From this point, the results obtained from the fluorescence measurements at high dilution should better reflect the activities of the compounds under the equilibrium conditions than those obtained by the NMR technique.

**Figure 3:**
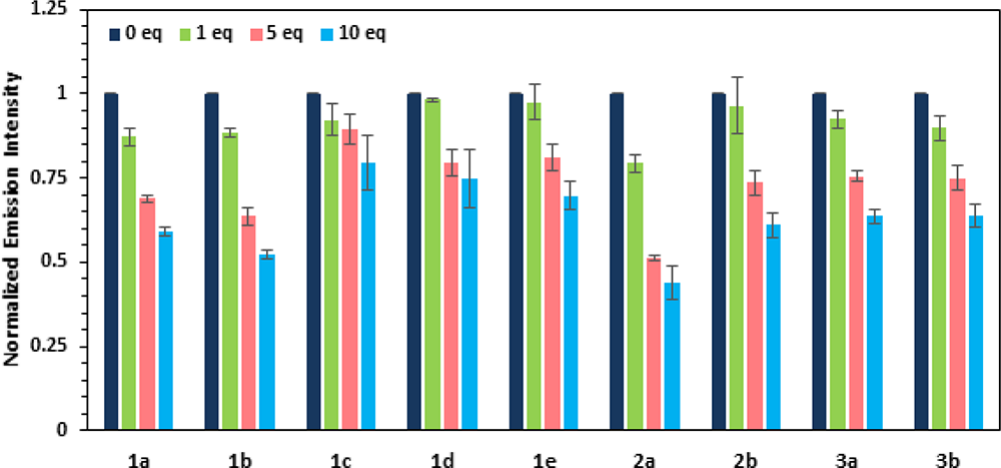
Normalized emission response of compounds **1**–**3** to the presence of the cationic **8**.

**Table 1:** Experimental *K*_a_ Values for the Host–Guest Complexation of Cationic **8**

Entry	Compound	*K*_a_ × 10^–3^ [M^–1^]
1	**2a**	12.7 ± 1.8
2	**1b**	9.2 ± 0.9
3	**1a**	6.7 ± 0.8
4	**2b**	6.6 ± 0.4
5	**3a**	5.7 ± 0.3
6	**3b**	5.5 ± 0.5
7	**1e**	4.5 ± 0.2
8	**1d**	3.7 ± 0.7
9	**1c**	2.4 ± 0.5

Importantly, the obtained binding constants show excellent correlation with the Hammett constants of the corresponding substituents (Figure 4).^15^ These results demonstrate that the electronic properties of the substituents affect the host–guest complexation of a cationic guest within a calixarene cavity.

**Figure 4:**
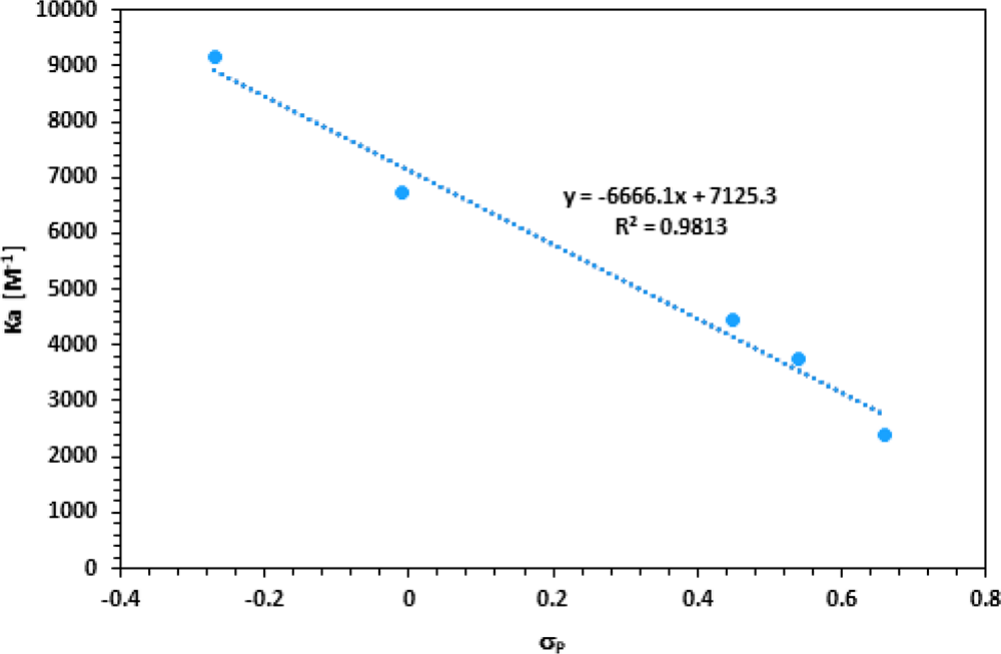
*K*_a_ dependence on the electronic properties (σ_*para*_) of the substituents in **1a**–**e**.

While it seems reasonable that the electron-donating substituents increase the binding of the guest within the calixarene cavity, their influence on the nature of the host–guest interactions deserves a comment. We obtained the structure of the core diethynyl-5,5′-bicalixarene receptor **7**(6) which shows relatively parallel arrangement of the deoxygenated aromatic ring and the ring opposite to it (Figure 5a). Other available X-ray structures of the mono- and bicalixarenes bearing an ethynyl group at the lower rim demonstrate a similar arrangement.^6,8,11^ Moreover, the remaining phenolic rings are pitched toward each other and engage in the hydrogen bonding (Figure 5a), making them less likely available for the interaction with the organic guest cation.^16^ We very recently provided evidence for the host–guest interactions involving **8** and predominantly the oxygen-depleted aromatic ring.^9^ Significantly stronger ^1^H NMR chemical shifts, and higher binding constants, observed for the cation of **8** in the bicalixarene systems as compared to single calixarene cavities suggest that this cation is trapped inside the closed bicalixarene capsule.^5,7,10^ Indeed, such a closed capsule was density functional theory (DFT) calculated at the B3LYP-D3/6-31G(d,p) level of theory to be thermodynamically favored over the open structure by over 3 kcal/mol (see SI). Although there is no crystallographic information on a *N*-methylpyridinium cation complexation within the cavity of a calix[4]arene, it is conceivable that the interactions between the two planar moieties lead to a shifted (slipped) structure potentially due to π–π and cation−π interactions (Figure 5b). The ^1^H NMR measurements of the complexation between **1b**, **c** and **8** demonstrated larger Δδ changes for the protons H_a,a′_ (downfield shifted) and H_b,b′_ (upfield shifted) upon the increased concentrations of **8**, compared with other aromatic protons of the host molecules, suggesting stronger interactions of the coplanar aromatic rings with the aromatic cationic guest.

**Figure 5:**
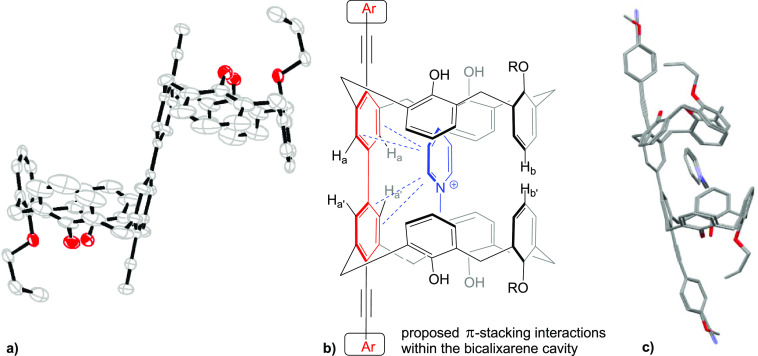
(a) ORTEP structure of the oxygen-depleted 5,5′-bicalixarene **7**; (b) proposed interactions between the 5,5′-bicalixarene host and cation of **8**; (c) superposition of the calculated structure of **8@1b** and **8@1c**.

Further support for such an organization of the guest within the bicalixarene cavity was obtained from the DFT calculations of the structures of **8@1b**,**c** (Figure 5c and SI). The complexation of cationic **8** within the bicalixarene capsule gives very similar structures for both electron-rich **1b** and electron-poor **1c**. Despite the slightly twisted conjugated biphenylene fragment (dihedral angle of 26°), the guest is situated nearly parallel to it. Importantly, the complexation of **8** in **1b** is favored over the complexation in **1c** by ca. 7 kcal/mol, testifying to the importance of the electron density on the biphenylene part of the host molecule. While the nature of the aromatic π stacking was proposed to involve the electrostatic interactions between the aromatic rings,^17^ a notion supported by the Hammett analysis of the substituent effect, a number of theoretic studies revealed that the aromatic π systems of the opposing rings remain unperturbed. Instead, the observed substituent effects were attributed to the direct disperse interactions between the substituents and the aromatic system.^18,19^ With two forces competing with each other, the dispersion and the electrostatic repulsion, the common motif of a slipped π stacking shows that the van der Waals interactions are more dominant.^20^ Interestingly, it was also shown that as the substituents become farther removed from the interacting aromatic groups, the direct interactions between the two rings may become stronger.^21^ Clearly, the electronic density at the bicalixarene aromatic rings modulated by the remote substituents plays an important role in the host–guest interactions, thus providing support for the direct aromatic π interactions.^22^ From this point, compounds **1**–**3** with spatially remote conjugated substituents in a sterically protected aromatic cavity can be an excellent model to further explore the nature of these interactions.

## Experimental Section

### General

The synthetic manipulations involving air-sensitive compounds were performed in a nitrogen filled Innovative Technology or Vigor glovebox. All solvents were degassed and stored under high-purity nitrogen and activated with 4 Å molecular sieves. All deuterated solvents were stored under high-purity nitrogen on 3 Å molecular sieves. Commercially available reagents (Aldrich, Strem, and Acros) were used as received. Heating was performed by using an oil bath with a temperature-controlled internal heater. The NMR spectra were recorded on a Bruker Avance 400 MHz spectrometer. ^1^H and ^13^C NMR signals are reported in ppm downfield from TMS. All measurements were performed at 22 °C in CDCl_3_/CD_2_Cl_2_ unless stated otherwise. CombiFlash NextGen 300+ with silica-filled columns was used for chromatographic purifications unless stated otherwise. Mass spectra were recorded on a VG-Autospec M-250 instrument. UV and fluorescence spectra were recorded on a Vernier fluorescence/UV–vis spectrophotometer and Hitachi F-2710 fluorescence spectrophotometer. Compounds **1a**, **2a**, **4**–**10** (SI), **13** (SI) were reported previously.^7b^

Association constant (*K*_a_, M^–1^) values for the formation of the complexes between the *N*-Methylpyridinium triflate (**8**) and the 5,5′-Bicalixarene derivative (**1**–**3**) were determined by fluorescence titration experiments in a 9:1 CHCl_3_–CH_3_CN mixture.^7b^ The initial (*F*_0_) and measured (*F*) fluorescence intensity ratio (*F*_0_/*F*) against the concentration of the quencher [**8**] was plotted, and the association constant was obtained from the equation *F*_0_/*F* = 1 + *K*_a_[**8**].

### Synthesis of Compounds **1**–**3**

#### **1b**

The compound was prepared using the procedure previously reported for **1a**.^7b^ Compound **7** (38 mg, 1.0 equiv, 0.040 mmol) was added to a solution of Pd(PPh_3_)_4_ (2.3 mg, 0.05 equiv, 0.002 mmol), CuI (0.4 mg, 0.05 equiv, 0.002 mmol), and 4-iodoanisole (93.6 mg, 10.0 equiv, 0.4 mmol) in Et_3_N/DMF (3:7), and the reaction mixture was stirred for 72 h at room temperature. The solvent was evaporated, and the resulting crude product was dissolved in CH_2_Cl_2_ and washed with brine several times. Drying the CH_2_Cl_2_ extract over MgSO_4_ followed by solvent removal under a vacuum gave the crude product. The residue was subjected to column chromatography (hexane–CH_2_Cl_2_, 1:2) to give compound **1b** as a white solid. Yield: 60% (28 mg). ^1^H NMR 7.61–7.65 (m, 4H), 7.00–7.10 (m, 12H), 6.85–6.91 (m, 8H), 6.63–6.76 (m, 10H), 4.74 (d, *J* = 13.0 Hz, 4H), 4.11 (d, *J* = 13.5 Hz, 4H), 3.93 (t, *J* = 6.5 Hz, 4H), 3.85 (s, 6H), 3.53 (d, *J* = 13.2 Hz, 4H), 3.43 (d, *J* = 13.6 Hz, 4H), 1.84 (sext, *J* = 7.2 Hz, 4H), 0.96 (t, *J* = 7.5 Hz, 6H). ^13^C{^1^H} NMR 159.6, 153.6, 151.5, 142.0, 139.4, 133.2, 129.5, 129.4, 129.0, 128.4, 127.4, 125.9, 121.5, 119.5, 116.7, 114.0, 97.4, 87.4, 78.9, 55.5, 36.4, 31.8, 23.4, 10.7. HRMS (ESI-TOF) *m*/*z*: [M + Na]^+^ calcd for C_80_H_70_O_8_Na 1181.4968; found 1181.4987.

#### **1c**

To a mixture of P(*t*-Bu)_3_H^+^ BF_4_^–^ (4.8 mg, 0.2 equiv, 0.0164 mmol) and Pd_2_dba_3_ (3.7 mg, 0.05 equiv, 0.0041 mmol) dissolved in 20 mL of dry DMF, CuI (39 mg, 2.5 equiv, 0.205 mmol), DBU (48 μL, 4 equiv, 0.328 mmol), 4-ethynylbenzonitrile (104.2 mg, 10 equiv, 0.82 mmol) and **6** (100 mg, 1.0 equiv, 0.082 mmol) were added, and the solution was heated at 80 °C for 12 h. The solvent was evaporated, and the resulting crude product was dissolved in CH_2_Cl_2_ and washed with brine several times. Drying the CH_2_Cl_2_ extract over MgSO_4_ followed by solvent removal under vacuum gave the crude product. The residue was subjected to column chromatography (hexane–CH_2_Cl_2_, 1:2) to give a light yellow solid of **1c**. Yield: 38% (35 mg). ^1^H NMR 7.75–7.79 (m, 4H), 7.65 (d, *J* = 8.3 Hz, 4H), 7.06–7.14 (m, 12H), 6.90 (d, *J* = 7.6 Hz, 4H), 6.69–6.78 (m, 10H), 4.75 (d, *J* = 12.8 Hz, 4H), 3.98–4.02 (m, 8H), 3.49–3.55 (m, 8H), 1.80 (sext, *J* = 7.2 Hz, 4H), 0.89 (t, *J* = 7.4 Hz, 6H). ^13^C{^1^H} NMR 153.3, 150.6, 142.7, 140.1, 132.9, 132.0, 130.1, 129.9, 129.6, 129.1, 128.5, 126.6, 126.5, 126.1, 121.1, 119.8, 119.0, 110.6, 95.0, 94.6, 79.5, 36.3, 31.8, 23.2, 10.6. HRMS (ESI-TOF) *m*/*z*: [M+ Na]^+^ calcd for C_80_H_64_N_2_O_6_Na 1171.4662; found 1171.4664.

#### **1d**

The product was obtained similarly to **1c**, using 4-ethynyl-α,α,α-trifluorotoluene instead of 4-ethynylbenzonitrile. The reaction residue was subjected to column chromatography (hexane–CH_2_Cl_2_, 1:1) to give **1d** as a white solid. Yield: 28% (28 mg). ^1^H NMR 7.79(d, *J* = 8.1 Hz, 4H), 7.61 (d, *J* = 8.2 Hz, 4H), 7.11–7.14 (m, 8H), 7.05 (d, *J* = 7.5 Hz, 4H), 6.94 (d, *J* = 7.6 Hz, 4H), 6.73–6.79 (m, 10H), 4.77 (d, *J* = 12.8 Hz, 4H), 3.95–4.04 (m, 8H), 3.48–3.56 (m, 8H), 1.80 (sext, *J* = 7.2 Hz, 4H), 0.87 (t, *J* = 7.4 Hz, 6H). ^13^C{^1^H} NMR 153.4, 150.8, 142.5, 139.9, 133.0, 131.8, 129.8, 129.6, 129.1, 128.8, 128.5, 126.7, 126.4, 126.1, 125.2, 125.1, 121.2, 119.7, 95.0, 92.4, 79.4, 36.3, 31.8, 23.2, 10.4. ^19^F NMR −63.08. HRMS (ESI-TOF) *m*/*z*: [M+ Na]^+^ calcd for C_80_H_64_F_6_O_6_Na 1257.4505; found 1257.4507.

#### **1e**

The product was obtained in a procedure similar to that for **1c** using 4-ethynylbenzoate instead of 4-ethynylbenzonitrile. The residue was subjected to column chromatography (hexane–CH_2_Cl_2_, 3:7) to afford a yellowish solid of **1e**. Yield: 26% (26 mg). ^1^H NMR (8.03 (d, *J* = 8.5 Hz, 4H), 7.74 (d, *J* = 8.4 Hz, 4H), 7.10–7.13 (m, 6H), 7.05 (dd, *J* = 7.4, 1.7 Hz, 4H), 6.92 (d, *J* = 7.6 Hz, 4H), 6.72–6.76 (m, 8H), 4.77 (d, *J* = 12.8 Hz, 4H), 3.94–4.05 (m, 14H), 3.47–3.56 (m, 4H), 1.80 (sext, *J* = 7.6 Hz, 4H), 0.89 (t, *J* = 7.4 Hz, 6H). ^13^C{^1^H} NMR 167.0, 153.4, 150.8, 142.5, 139.8, 132.8, 131.5, 130.4, 129.8, 129.6, 129.5, 129.1, 128.5, 126.7, 126.4, 126.0, 121.4, 119.6, 95.8, 93.0, 79.4, 52.3, 36.4, 31.8, 23.3, 10.6. HRMS (ESI-TOF) *m*/*z*: [M+ Na]^+^ calcd for C_82_H_70_O_10_Na 1237.4868; found 1237.4878.

#### **2b**

P(*t*-Bu)_3_H^+^BF_4_^–^ (4 mg, 0.014 mmol) and Pd_2_(dba)_3_ (3.2 mg, 0.004 mmol) dissolved in 10 mL of dry DMF were stirred under nitrogen at room temperature for 20 min. CuI (32 mg, 0.17 mmol), DBU (42 μL, 0.28 mmol), and **12** (113 mg, 0.34 mmol) were added, followed by dropwise addition of **6** (84 mg, 0.07 mmol) in 5 mL of dry DMF. The mixture was heated at 85 °C for 24 h. After the evaporation of the solvent, the crude product was dissolved in CH_2_Cl_2_, washed with brine, and dried over MgSO_4_, and the solvent was evaporated. Purification with Combi*Flash* (CH_2_Cl_2_–hexane, 1:1) afforded **2b** as a white solid. Yield: 17% yield (18 mg). ^1^H NMR 8.33 (d, *J* = 1.0 Hz, 2H), 8.12 (d, *J* = 7.7 Hz, 2H), 7.70–7.78 (m, 4H), 7.67 (dd, *J* = 8.5, 1.6 Hz, 2H), 7.56–7.63 (m, 4H), 7.43–7.54 (m, 6H), 7.32 (s, 4H), 7.27 (ddd, *J* = 7.9, 6.7, 1.4 Hz, 3H), 7.10–7.17 (m, 4H), 7.08 (dd, *J* = 7.6, 1.5 Hz, 4H), 7.04 (d, *J* = 7.6 Hz, 4H), 6.80–6.90 (m, 6H), 6.74 (t, *J* = 7.5 Hz, 3H), 4.79 (d, *J* = 12.8 Hz, 4H), 4.31 (t, *J* = 7.2 Hz, 4H), 4.00–4.12 (m, 8H), 3.50–3.60 (m, 8H), 1.85–1.98 (m, 8H), 0.93–1.05 (m, 12H). ^13^C{^1^H} NMR 153.7, 151.2, 142.6, 141.4, 140.7, 140.1, 133.6, 131.8, 131.6, 130.1, 129.7, 129.5, 129.3, 129.1, 128.7, 126.8, 126.6, 126.4, 124.5, 124.3, 123.6, 123.2, 122.7, 121.8, 120.8, 120.1, 119.8, 113.3, 109.5, 109.4, 96.5, 92.9, 91.7, 87.9, 80.0, 45.2, 36.4, 31.9, 23.6, 22.7, 11.9, 10.8. HRMS (ESI-TOF) *m*/*z*: [M + Na]^+^ calcd for C_112_H_92_N_2_O_6_Na 1583.6853, found 1583.6864.

#### **3a**

The product was obtained using compound **15** instead of **12**, in a procedure similar to the preparation of **2b**. The crude product was purified with Combi*Flash* (CH_2_Cl_2_–hexane, 1:1) to afford white solid **3a**. Yield: 27% (22 mg). ^1^H NMR 7.68–7.70 (m, 4H), 7.53–7.55 (m, 4H), 7.30 (s, 4H), 7.18 (brs, 4H), 7.11 (dd, *J* = 7.5, 1.3 Hz, 4H), 7.06 (dd, *J* = 7.6, 1.5 Hz, 4H), 7.00–7.02 (m, 7H), 6.80–6.85 (m, 6H), 6.73 (t, *J* = 7.5 Hz, 3H), 4.77 (d, *J* = 12.7 Hz, 4H), 3.98–4.07 (m, 8H), 3.50–3.55 (m, 8H), 2.32 (s, 12H), 1.81–1.90 (m, 4H), 0.94 (t, *J* = 7.4 Hz, 6H). ^13^C{^1^H} NMR 153.6, 151.1, 142.6, 140.1, 138.6, 133.5, 131.8, 130.8, 130.1, 129.7, 129.6, 129.3, 128.8, 126.8, 126.6, 126.4, 124.9, 123.0, 121.8, 120.1, 96.3, 91.9, 91.6, 89.0, 80.0, 36.4, 31.9, 23.6, 21.3, 10.7. HRMS (ESI-TOF) *m*/*z*: [M + H]^+^ calcd for C_98_H_83_O_6_ 1355.6190, found 1355.6173.

#### **3b**

The product was obtained by using compound **19** instead of **12**, in a procedure similar to the preparation of **2b**. The crude product was purified with Combi*Flash* (CH_2_Cl_2_–hexane, 1:1) to afford white solid **3b**. Yield: 43% (27 mg). ^1^H NMR 7.71 (d, *J* = 7.7 Hz, 4H), 7.56 (d, *J* = 8.1 Hz, 4H), 7.29–7.31 (m, 8H), 7.19 (s, 4H), 7.02–7.13 (m, 14H), 6.81–6.88 (m, 6H), 6.74 (t, *J* = 7.5 Hz, 4H), 4.78 (d, *J* = 12.7 Hz, 4H), 4.00–4.08 (m, 8H), 3.51–3.56 (m, 8H), 2.52 (s, 12H), 2.33 (s, 12H), 1.83–1.89 (m, 4H), 0.95 (t, *J* = 7.3 Hz, 6H). ^13^C{^1^H} NMR 153.6, 151.1, 142.6, 140.8, 140.1, 138.6, 133.6, 131.8, 130.8, 130.1, 129.7, 129.4, 129.3, 129.4, 128.8, 126.80, 126.6, 126.4, 125.2, 124.0, 123.4, 122.8, 122.4, 121.8, 120.1, 100.4, 96.3, 92.1, 91.4, 90.7, 86.4, 80.0, 36.3, 31.9, 23.6, 21.2, 21.2, 10.7. HRMS (ESI-TOF) *m*/*z*: [M + Na]^+^ calcd for C_118_H_98_O_6_Na 1633.7261, found 1633.7258.

## Data Availability

The data underlying this study are available in the published article and its Supporting Information.

## Supporting Information Available

Synthesis and characterization of all new compounds, UV–vis, fluorescence and NMR spectra, fluorescence and NMR complexation studies, and computational details (PDF)
