# Supplementary material for: Long Range Electronic Effects on the Host–Guest Complexation within the Oxygen Depleted 5,5′-Bicalixarene Cavities
Source: J Org Chem. 2023 Oct 31;88(22):15983–8. doi: 10.1021/acs.joc.3c01566 (PMC10661034; doi:10.1021/acs.joc.3c01566)
Supplement: Supplementary file 1 — jo3c01566_si_001.pdf [file jo3c01566_si_001.pdf]

Supporting Information

Long Range Electronic Effects on the Host-Guest Complexation within the Oxygen Depleted 5,5'-Bicalixarene Cavities.

Michal Farber, Pintu Maity, Abhishek Baheti, Adina Golombek, Tal Schwartz, Roman Dobrovetsky* and Arkadi Vigalok*

School of Chemistry, The Sackler Faculty of Exact Sciences, Tel Aviv University, Tel Aviv 69978, Israel.

*E-mail: avigal@tauex.tau.ac.il; rdobrove@tauex.tau.ac.il

Table of Contents

Contents	Page
1. General Information	S2
2. Synthesis of compounds **11-19**	S3-S5
3. NMR and Mass Spectra	S6-S28
4. Absorption and Emissions Spectra	S29-S39
5. NMR binding experiments	S39-S40
6. DFT calculations	S41-S64
7. References	S65

1. General Information

General. The synthetic manipulations involving air-sensitive compounds were performed in a nitrogen filled Innovative Technology or Vigor glove box. All solvents were degassed and stored under high-purity nitrogen and activated 4Å molecular sieves. All deuterated solvents were stored under high-purity nitrogen on 3Å molecular sieves. Commercially available reagents (Aldrich, Strem and Acros) were used as received. Heating was performed using an oil bath with a temperature-controlled internal heater. The NMR spectra were recorded on Bruker Avance 400MHz spectrometer. ^{1}H and ^{13}C NMR signals are reported in ppm downfield from TMS. All measurements were performed at 22 °C in $CDCl_3/CD_2Cl_2$ unless stated otherwise. CombiFlash® NextGen 300+ with silica-filled columns was used for chromatographic purifications unless stated otherwise. Mass Spectra were recorded on a VG-Autospec M-250 instrument. UV and Fluorescence spectra were recorded on Vernier fluorescence/UV-Vis spectrophotometer and Hitachi F-2710 fluorescence spectrophotometer. Compounds **1a**, **2a**, **4-10**, **13** were reported previously.[1]
Association constant (Ka, M^{-1}) values for the formation of the complexes between the N-Methyl pyridinium triflate (**8**) and the 5,5'-Bicalixarene derivative (**1-3**) were determined by fluorescence titration experiments in a 9:1 $CHCl_3$:CH_3CN mixture.[1] The initial (F_0) and measured (F) fluorescence intensity ratio (F_0/F) against the concentration of the quencher [**8**] was plotted and the association constant was obtained from the equation $F_0/F = 1 + Ka[\mathbf{8}]$.
Association constant (Ka, M^{-1}) values for the formation of the complexes between the N-Methyl pyridinium triflate (**8**) and **1b,c** were also determined by the ^{1}H NMR titration method in a 9:1 $CDCl_3$:CD_3CN mixture. The host concentration was kept constant at 0.5mM while the guest concentrations varied at 0.1, 0.2, 0.5, 1.0, 1.5, 2.5 and 5.0 mM. The constants were calculated from the equation: $\Delta\delta = \frac{\delta_{\Delta HG}}{2[H]_0}\left(\left([G]_0 + [H]_0 + \frac{1}{K}\right) - \sqrt{\left([G]_0 + [H]_0 + \frac{1}{K}\right)^2 - 4[G]_0[H]_0}\right)$ reported in ref. 2, using Excel Solver software. Fitting the data using online application at http://supramolecular.org/apps/ gave the same results.

2. Synthesis of compounds 11-19

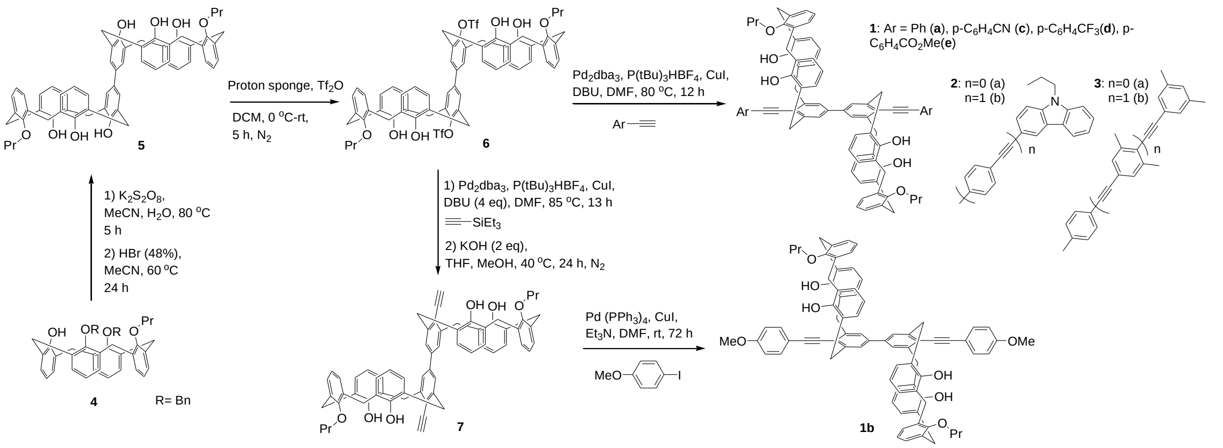

Scheme S1: General synthesis of compounds **1-3**.

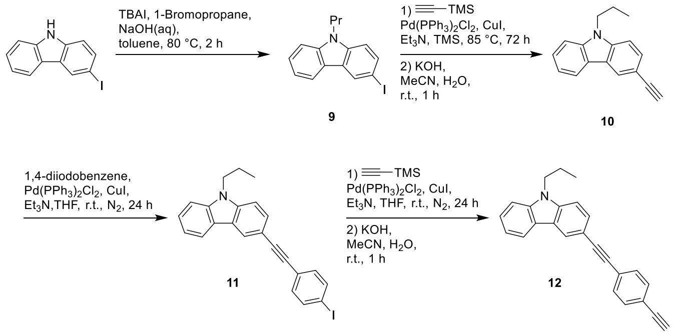

Scheme S2: General synthesis of compound **10** and **12**.

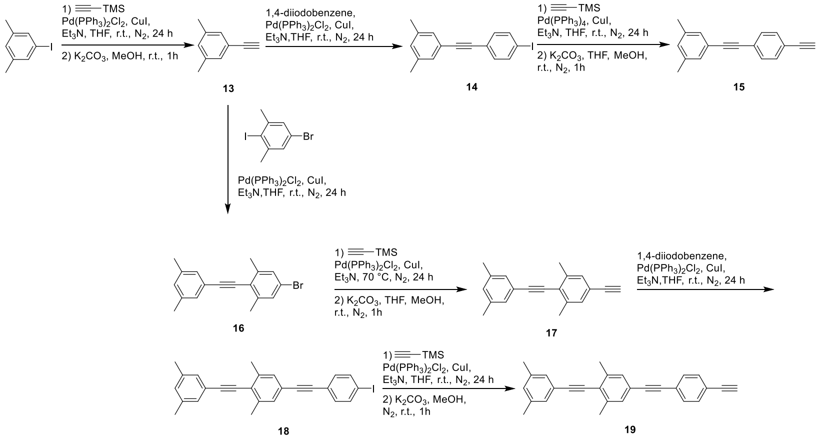

Scheme S3: General synthesis of compound **15** and **19**.

11: The synthesis was based on the literature procedure.[3] A mixture of 1,4-diiodobenzene (3.5 g, 10.5 mmol), $Pd(PPh_3)_2Cl_2$ (75.2 mg, 0.11 mmol) CuI (20.9 mg, 0.11 mmol), dry THF (25 mL) and Et_3N (3 mL, 21 mmol) was stirred under nitrogen atmosphere at room temperature for 5 minutes before dropwise addition of **10**[21] (0.5 g, 2.1 mmol). After stirring at room temperature for additional 24 hours, the solvent was evaporated under vacuum and the crude product was purified using Combi*Flash*® (CH_2Cl_2-hexane, 3:7) to give yellow solid **11**. Yield: 58% (0.53 g). ^1H NMR 8.30 (s, 1H), 8.10 (d, J = 7.7 Hz, 1H), 7.70 (d, J = 7.4 Hz, 2H), 7.63 (d, J = 8.5 Hz, 1H), 7.50 (dd, J = 8.1, 7.2 Hz, 1H), 7.42 (d, J = 8.1 Hz, 1H), 7.38 (d, J = 8.5 Hz, 1H), 7.25-7.31(m, 3H), 4.28 (t, J = 7.1 Hz, 2H), 1.93 (sext, J = 7.2 Hz, 2H), 0.99 (t, J = 7.2 Hz, 3H). $^{13}C\{^1H\}$ NMR141.0, 140.4, 137.6, 133.1, 129.3, 126.3, 124.2, 123.6, 123.0, 122.5, 120.6, 119.5, 112.9, 109.1, 109.0, 93.5, 92.6, 86.8, 44.9, 22.4, 11.9. HRMS (APPI-TOF) m/z: $[M+H]^+$ calcd for $C_{23}H_{19}IN$ 436.0562, found 436.0558.

12: The synthesis was based on the literature procedure.[4] Compound **11** (0.35 mg, 0.8 mmol), $Pd(PPh_3)_2Cl_2$ (28 mg, 0.04 mmol) CuI (7.6 mg, 0.04 mmol), dry THF (3 mL) and Et_3N (10 mL, 62 mmol) were stirred under nitrogen atmosphere at room temperature. After 5 minutes of stirring, ethynyltrimethylsilane (0.7 mL, 5 mmol) was added slowly, and the mixture was left stirring at room temperature for overnight. The solvent was removed under vacuum and the crude product was purified using Combi*Flash*® (CH_2Cl_2-hexane, 3:7) to obtain yellow solid. The protected product was dissolved in CH_3CN (15 mL) and after addition of water (1 mL) and potassium hydroxide (0.19 g, 3.3 mmol) the mixture was left stirring at room temperature for 1 hour. After removal of the solvent under vacuum, a solution of NH_4Cl (1M) was added and the solution was extracted with $CHCl_3$ (X3). The organic layers were combined and washed with NH_4Cl (1M) and water. The organic layer was dry over $MgSO_4$, filter and the solvent was evaporated under vacuum to afford yellow solid **12**. Yield: 79% (0.21 g). ^1H NMR 8.35 (s, 1H), 8.14 (d, J = 7.7 Hz, 1H), 7.67 (dd, J = 8.5, 1.5 Hz, 1H), 7.50-7.59 (m, 5H), 7.43 (d, J = 8.2 Hz, 1H), 7.38 (d, J = 8.5 Hz, 1H), 7.28-7.32 (m, 1H), 4.25 (t, J = 7.1 Hz, 2H), 3.22 (s, 1H), 1.92 (sext, J = 7.3 Hz, 2H), 0.99 (t, J = 7.4 Hz, 3H). $^{13}C\{^1H\}$ NMR 141.0, 140.4, 132.2, 131.4, 129.4, 126.2, 124.6, 124.2, 123.0, 122.5, 121.3, 120.6, 119.5, 112.9, 109.1, 108.9, 93.3, 87.3, 83.6, 78.8, 44.8, 22.4, 11.9. HRMS (APPI-TOF) m/z: $[M+H]^+$ calcd for $C_{25}H_{20}N$ 334.1596, found 334.1602.

14: The product obtained using compound **13**[5] instead of **10**, in a similar procedure as **11**. The crude product was purified using Combi*Flash*® (CH_2Cl_2-hexane, 3:7) to afford white solid **14**. Yield: 32% (1 g). ^1H NMR 7.67-7.69 (m, 2H), 7.23-7.25 (m, 2H), 7.17 (s, 2H), 6.99 (s, 1H), 2.32 (s, 6H). $^{13}C\{^1H\}$ NMR 138.1, 137.6, 133.2, 130.6, 129.4, 123.2, 122.7, 94.0, 91.3, 87.9, 21.2. HRMS (APPI-TOF) m/z: $[M]^+$ calcd for $C_{16}H_{13}I$ 332.0062, found 332.0057.

15: The procedure was similar to the reported in the literature.[6] Compound **14** (1 g, 3.2 mmol), $Pd(PPh_3)_4$ (0.37 mg, 0.32 mmol), CuI (61 mg, 0.32 mmol), dry THF (40 mL) and Et_3N (4.5 mL, 32 mmol) were added under nitrogen and stirred at room temperature. After 5 minutes, ethynyltrimethylsilane (4.4 mL, 32 mmol) was added slowly and the mixture left stirring at room temperature for overnight. After removal of the solvent the product was purified by column chromatography with silica gel (hexane) to give yellow oil. The protected product was dissolved in dry THF (20 mL) and treated with MeOH (60 mL) and K_2CO_3 (0.47 g, 3.4 mmol) under nitrogen atmosphere. The mixture was stirred at room temperature for 1.5 hours. Water was added, and the mixture was extracted with CH_2Cl_2 (X3). The organic layers were dried over $MgSO_4$, filtered, and concentrated to dryness to afford orange oil **15**. Yield: 24% (0.18 g). ^1H NMR 7.46 (s, 4H), 7.17 (brs, 2H), 6.99 (brs, 1H), 3.17 (s, 1H), 2.32 (s, 6H). $^{13}C\{^1H\}$ NMR 138.1, 132.2, 131.6, 130.6, 129.4, 124.1, 122.6, 121.8, 91.9, 88.3, 83.5, 78.9, 21.2. HRMS (APPI-TOF) m/z: $[M]^+$ calcd for $C_{18}H_{1\ 4}$230.1096, found 230.1100.

16: The procedure is based on the reported literature method.[6] 4-Bromo-2,6-dimethyliodobenzene (14 g, 45 mmol), $Pd(PPh_3)_2Cl_2$ (0.10 g, 0.15 mmol), CuI (28 mg, 0.15 mmol), dry THF (25 mL) and Et_3N (21 mL, 150 mmol) were stirred under nitrogen at room temperature for 5 minutes. Compound **13** was added dropwise, and the mixture was stirred under nitrogen atmosphere at room temperature for overnight. The solvent was removed, and the residue was dissolved in CH_2Cl_2 and washed with water (X3) and brine (X3). The organic layer was dried over $MgSO_4$, filtered, and concentrated to dryness under vacuum. The crude product was purified by column chromatography with silica gel (hexane) to give white solid 16. Yield: 48% (2.2 g). ^1H NMR 7.24 (brs, 2H), 7.17 (brs, 2H), 7.00 (brs, 1H), 2.49 (s, 6H), 2.34 (s, 6H). $^{13}C\{^1H\}$ NMR δ: 142.2, 138.2, 130.4, 129.8, 129.2, 123.2, 122.4, 121.7, 99.4, 85.7, 21.3, 21.0. HRMS (APPI-TOF) m/z: $[M]^+$ calcd for $C_{18}H_{17}Br$ 312.0514, found 312.0506.

17: The procedure is based on the literature method.[7] Compound **16** (2.2 g, 7.2 mmol), $Pd(PPh_3)_2Cl_2$ (0.10 g, 0.14 mmol), CuI (27 mg, 0.14 mmol) and Et_3N (30 mL, 216 mmol) were stirred under nitrogen at room temperature. After 5 minutes ethynyltrimethylsilane (5 mL, 36 mmol) was added dropwise and the reaction mixture was stirred under nitrogen atmosphere at 70 °C for overnight. The mixture was diluted with DCM and washed with water (X3) and brine (X3). The organic layer was dried over $MgSO_4$, filtered, and concentrated under reduced pressure. The crude product was purified by column chromatography with silica gel (hexane) to give bright yellow solid. The TMS protected product was dissolved in dry THF (45 mL) and after MeOH (135 mL) addition was treated with K_2CO_3 (2 g, 14.4 mmol). The mixture was left standing under nitrogen atmosphere at room temperature for overnight. After removal of the solvents, water was added, and the mixture was extracted with CH_2Cl_2 (X2). The organic layer was washed with brine, dried over $MgSO_4$, filtered, and the solvent evaporated under reduced pressure. The crude product was purified by column chromatography with silica gel (hexane) to give bright yellow solid **17**. Yield: (0.57 g, 31% yield). ^1H NMR 7.21 (brs, 2H), 7.17 (brs, 2H), 6.99 (brs, 1H), 3.10 (s, 1H), 2.48 (s, 6H), 2.33 (s, 6H). $^{13}C\{^1H\}$ NMR 140.3, 138.1, 130.3, 130.4, 129.2, 124.2, 123.2, 121.1, 100.1, 86.1, 83.9, 78.0, 21.2, 21.0. HRMS (APPI-TOF) m/z: $[M]^+$ calcd for $C_{20}H_{18}$ 258.1409, found 258.1412.

18: The product obtained using compound **17** instead of **10**, in a similar procedure as **11**. The crude product was purified by column chromatography with silica gel (hexane) to afford bright yellow solid **18**. Yield: 67% (0.68 g). ^{1}H NMR 7.68-7.70 (m, 2H), 7.23-7.25 (m, 4H), 7.18 (brs, 2H), 6.99 (brs, 1H), 2.50 (s, 6H), 2.33 (s, 6H). ^{13}C{^{1}H} NMR 140.4, 138.1, 137.6, 133.2, 130.4, 129.9, 129.2, 123.9, 123.2, 122.9, 121.8, 100.2, 94.2, 91.2, 89.5, 86.3, 21.3, 21.1. HRMS (APPI-TOF) m/z: $[M]^+$ calcd for $C_{26}H_{21}I$ 460.0688, found 460.0685.

19: Compound **18** (0.68 g, 1.48 mmol), $Pd(PPh_3)_2Cl_2$ (15.6 mg, 0.02 mmol) CuI (4.3 mg, 0.02 mmol), dry THF (20 mL) and Et_3N (2 mL, 14.8 mmol) were stirred under nitrogen atmosphere at room temperature. After 5 minutes of stirring, ethynyltrimethylsilane (1 mL, 7.5 mmol) was added slowly, and the mixture was left stirring at room temperature for overnight. The solvent was removed under vacuum and the residue was dissolved in DCM. After washings with water and brine, the organic layer was dried over $MgSO_4$, filtered and evaporated to dryness under vacuum. The crude product was purified by column chromatography with silica gel (hexane) to give bright yellow solid. The TMS protected product was dissolved in dry THF (16 mL) and after MeOH (48 mL) addition was treated with K_2CO_3 (0.73 g, 5.3 mmol) under nitrogen atmosphere. The mixture was stirred at room temperature for 5 hours. After removal of the solvents the residue was dissolved in CH_2Cl_2 and was washed with water and brine. The organic layer was dried over $MgSO_4$, filtered, and concentrated to dryness to give bright yellow solid **19**. Yield: 60% (0.32 g). ^{1}H NMR 7.48 (brs, 4H), 7.26 (brs, 2H), 7.19 (brs, 2H), 7.00 (brs, 1H), 3.19 (s, 1H), 2.52 (s, 6H), 2.34 (s, 6H). ^{13}C{^{1}H} NMR 140.4, 138.1, 132.2, 131.6, 130.5, 129.9, 129.2, 123.9, 123.9, 123.3, 122.0, 121.9, 100.2, 91.8, 89.9, 86.3, 83.4, 79.0, 21.3, 21.1. HRMS (APPI-TOF) m/z: $[M]^+$ calcd for $C_{28}H_{22}$ 358.1722, found 358.1723.

3. NMR and Mass spectra

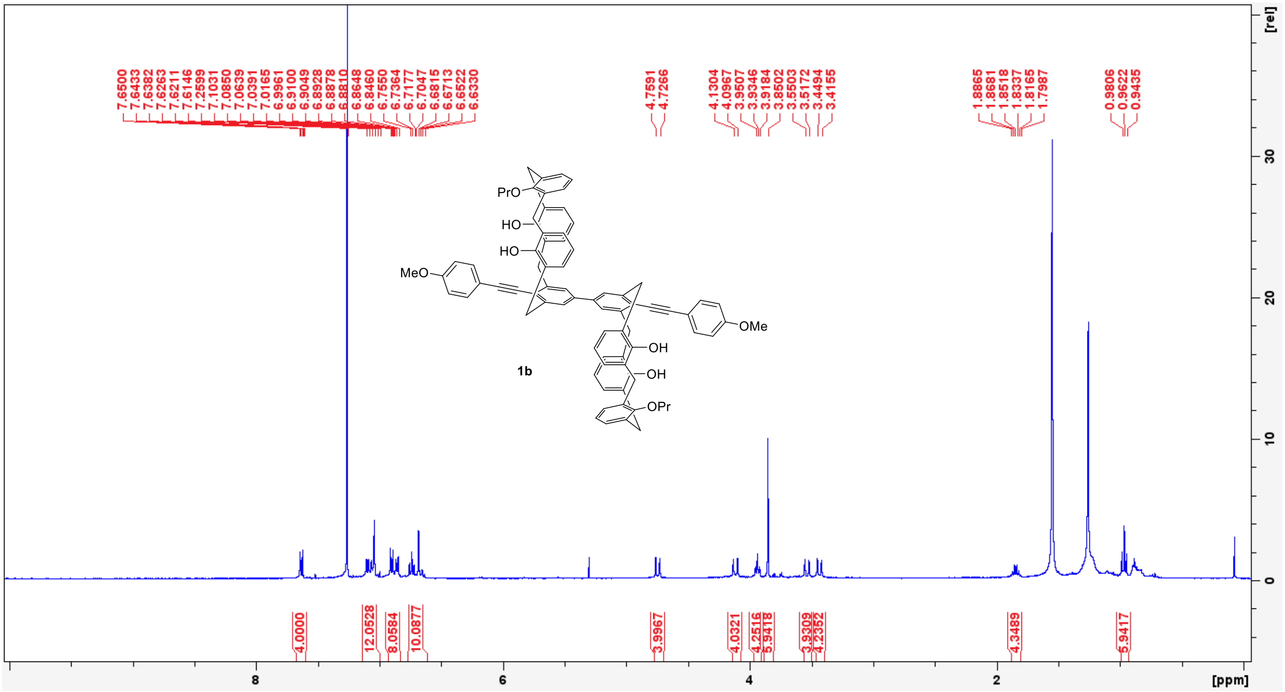

Fig. S1 ^{1}H NMR (400 MHz) spectrum of 1b in $CDCl_3$

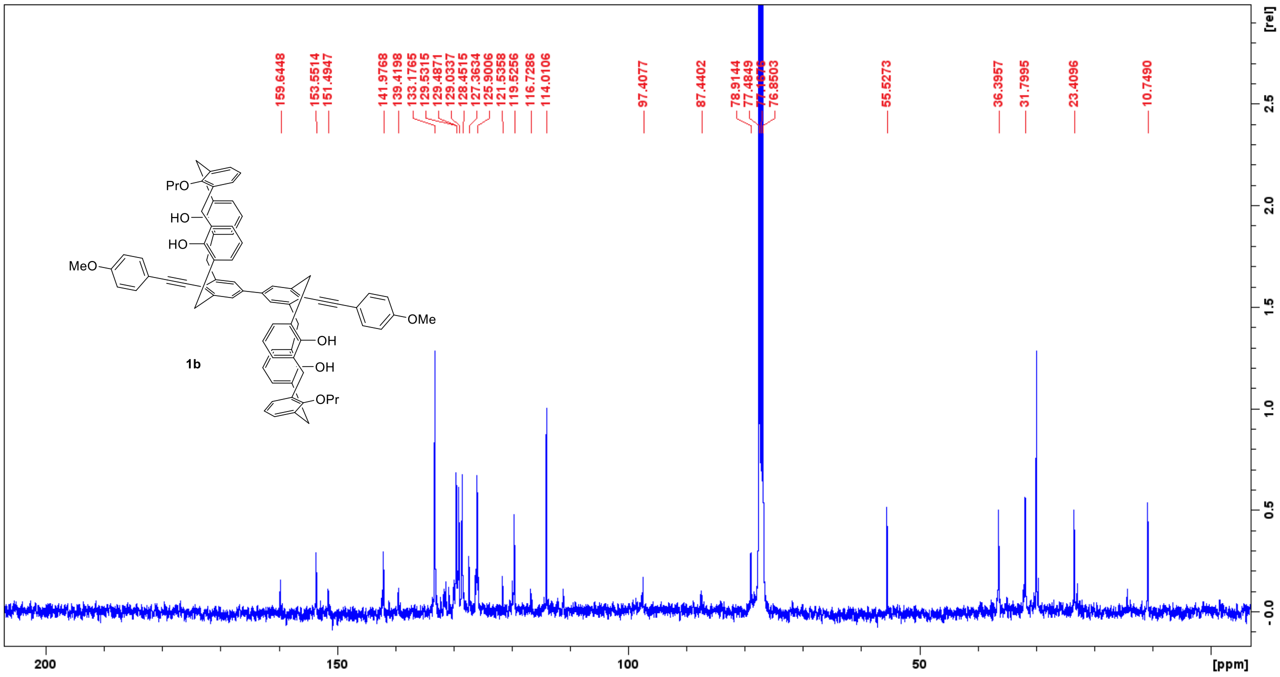

Fig. S2 $^{13}C\{^{1}H\}$ NMR (101 MHz) spectrum of 1b in $CDCl_3$

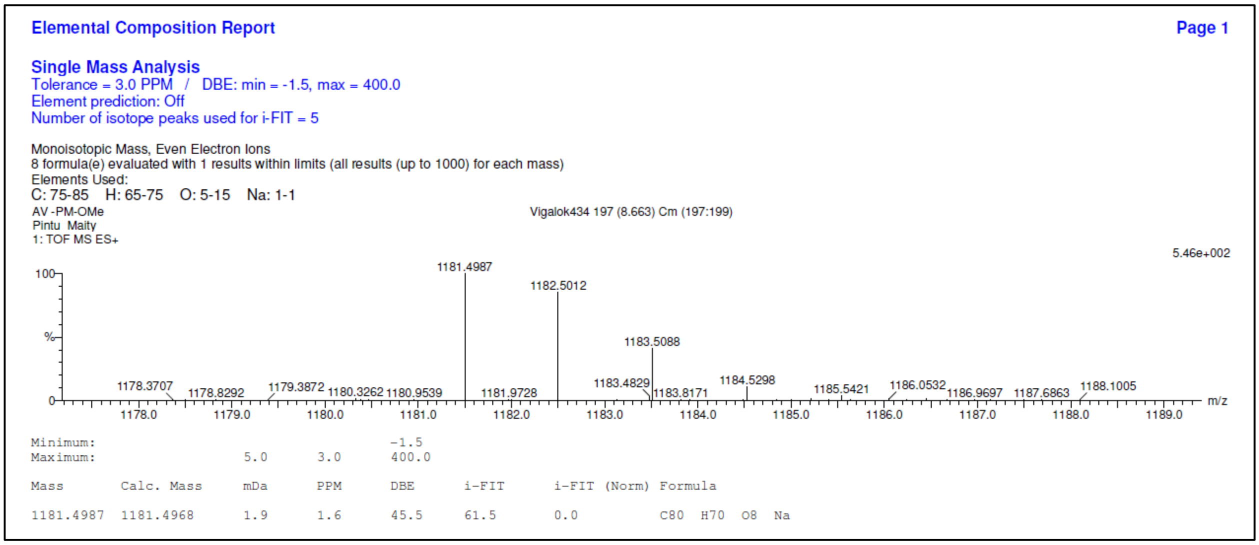

Fig. S3 Mass spectrum of 1b

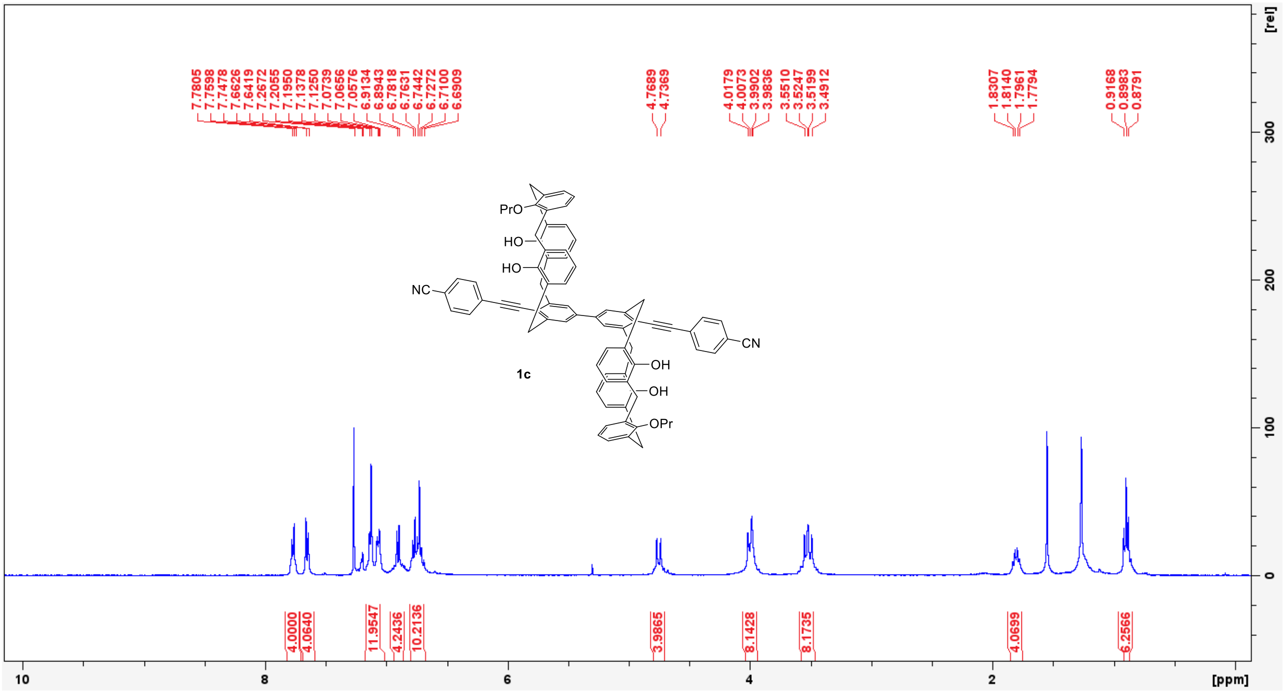

Fig. S4 ^{1}H NMR (400 MHz) spectrum of 1c in $CDCl_3$

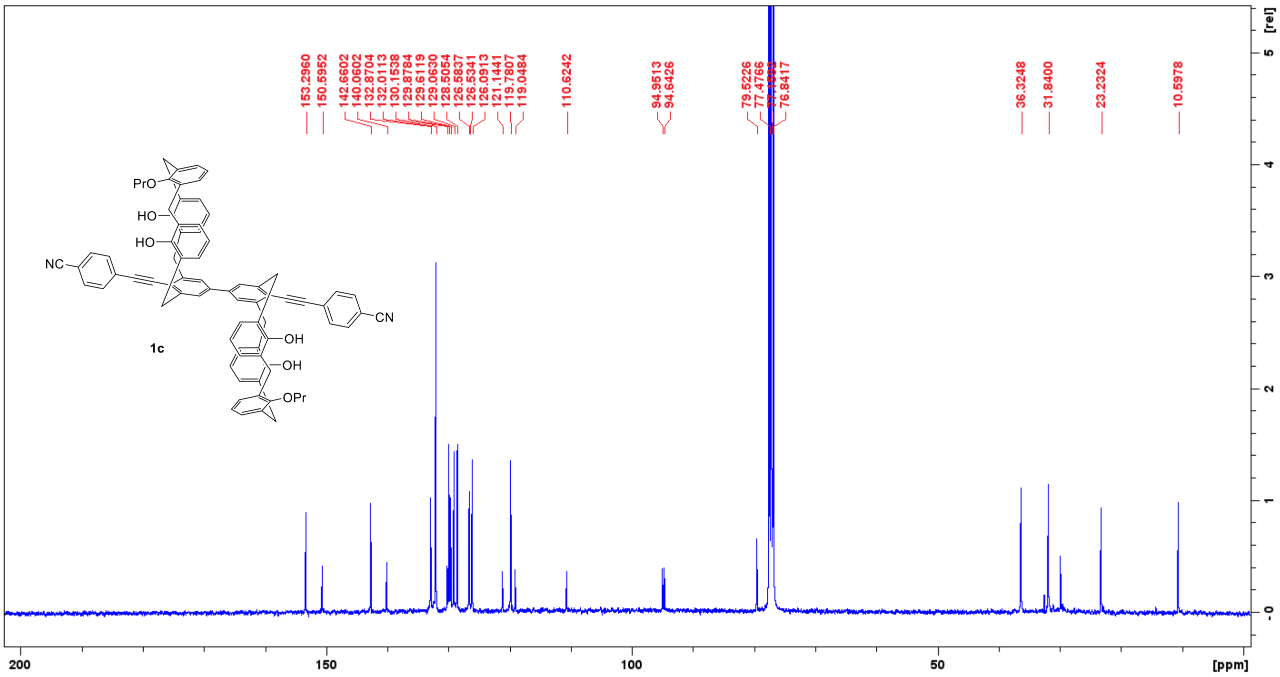

Fig. S5 $^{13}C\{^{1}H\}$ **NMR (101 MHz) spectrum of 1c in $CDCl_3$**

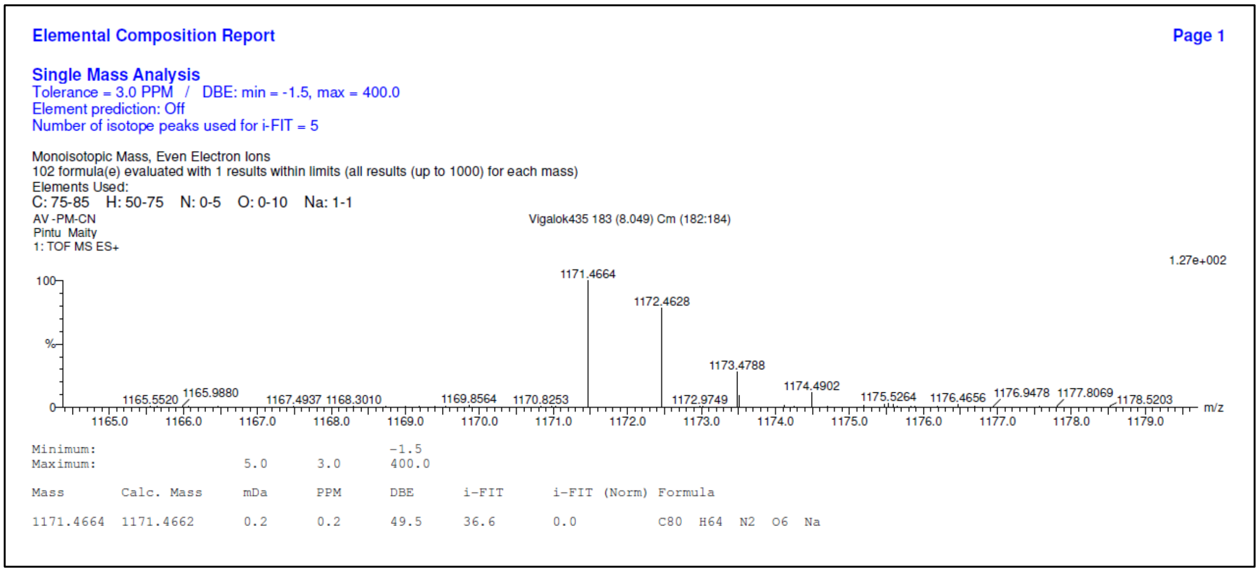

Fig. S6 Mass spectrum of 1c

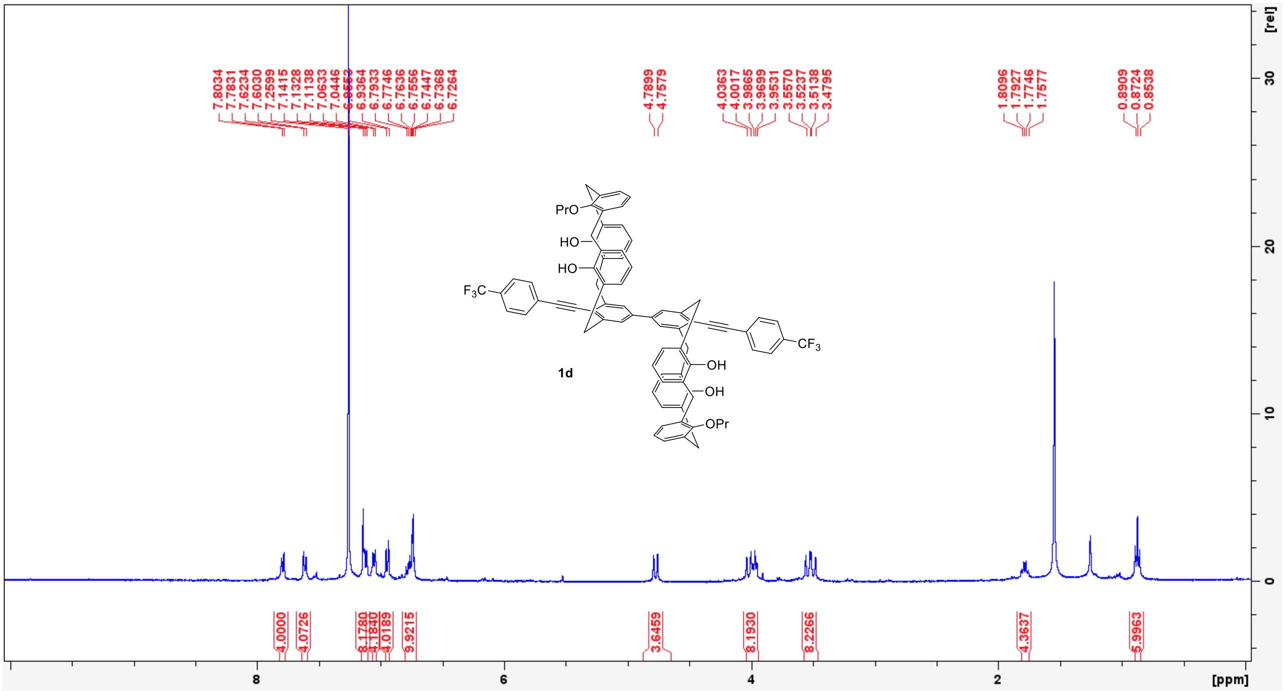

Fig. S7 ^{1}H NMR (400 MHz) spectrum of 1d in $CDCl_3$

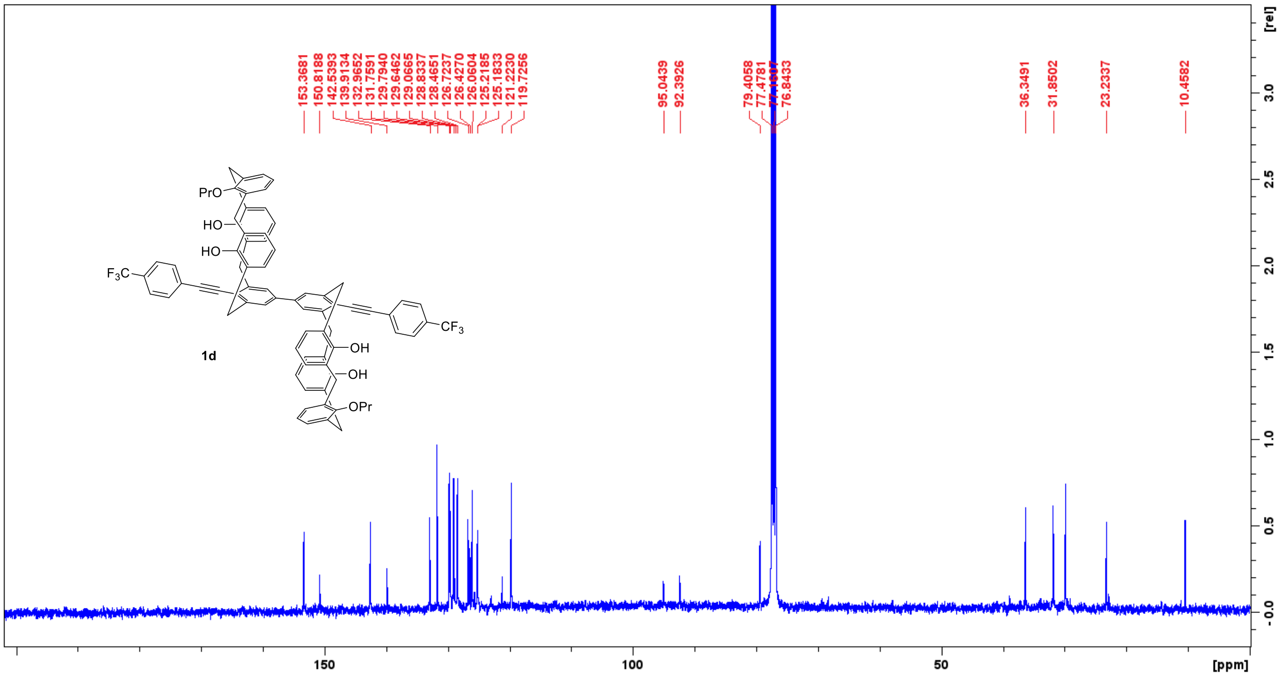

Fig. S8 $^{13}C\{^{1}H\}$ NMR (101 MHz) spectrum of 1d in $CDCl_3$

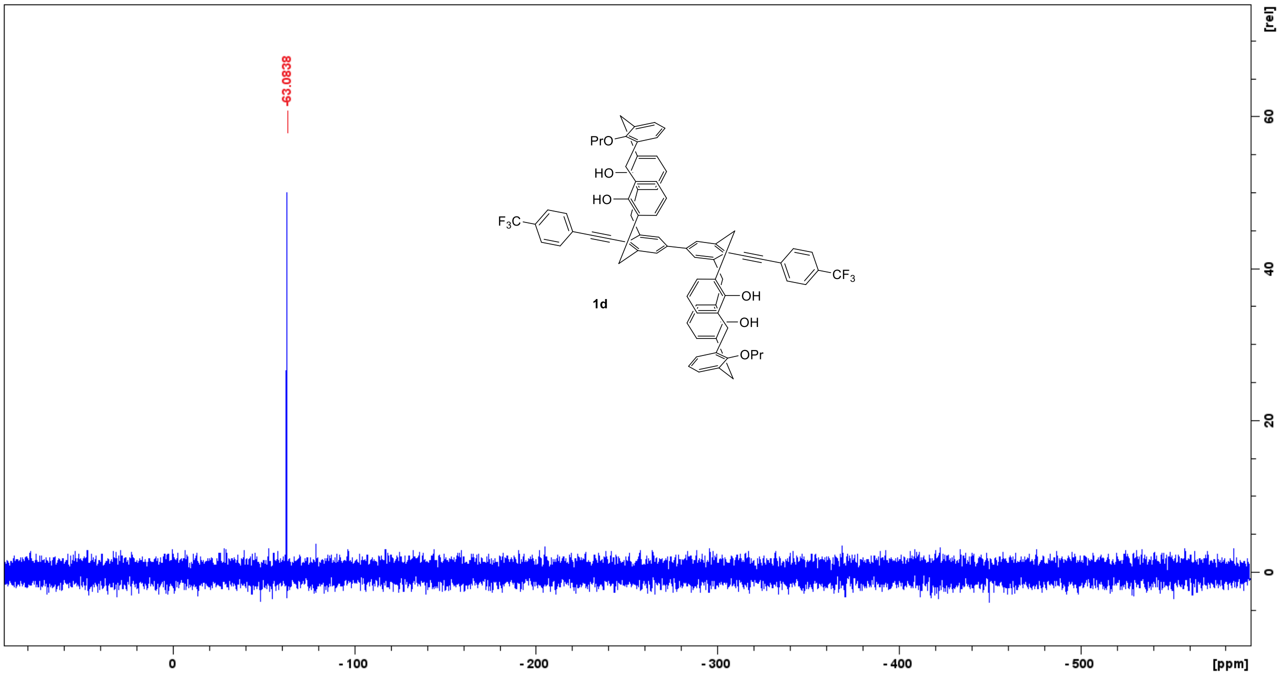

Fig. S9 ^{19}F NMR (376 MHz) spectrum of 1d in $CDCl_3$

Elemental Composition Report **Page 1**

Single Mass Analysis
Tolerance = 3.0 PPM / DBE: min = -1.5, max = 400.0
Element prediction: Off
Number of isotope peaks used for i-FIT = 5

Monoisotopic Mass, Even Electron Ions
23 formula(e) evaluated with 3 results within limits (all results (up to 1000) for each mass)
Elements Used:
C: 78-82 H: 60-70 O: 2-10 Na: 1-1 F: 0-8

AV -PM-CF3
Pintu Maity
1: TOF MS ES+

Vigalok436 31 (1.385) Cm (23:31)

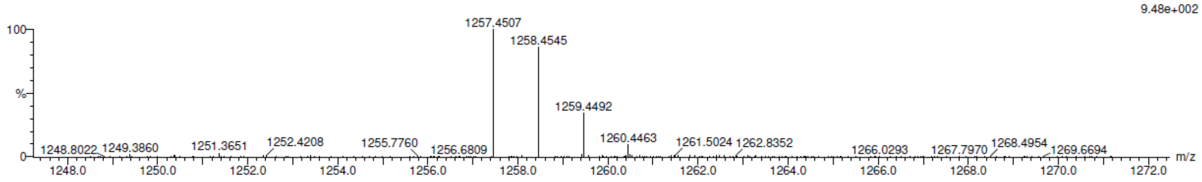

Mass	Calc. Mass	mDa	PPM	DBE	i-FIT	i-FIT (Norm)	Formula
Minimum:				-1.5			
Maximum:		5.0	3.0	400.0			
1257.4507	1257.4505	0.2	0.2	45.5	144.0	0.3	C80 H64 O6 Na F6
	1257.4529	-2.2	-1.7	48.5	146.1	2.5	C82 H65 O8 Na F3
	1257.4541	-3.4	-2.7	44.5	145.5	1.8	C79 H66 O9 Na F4

Fig. S10 Mass spectrum of 1d

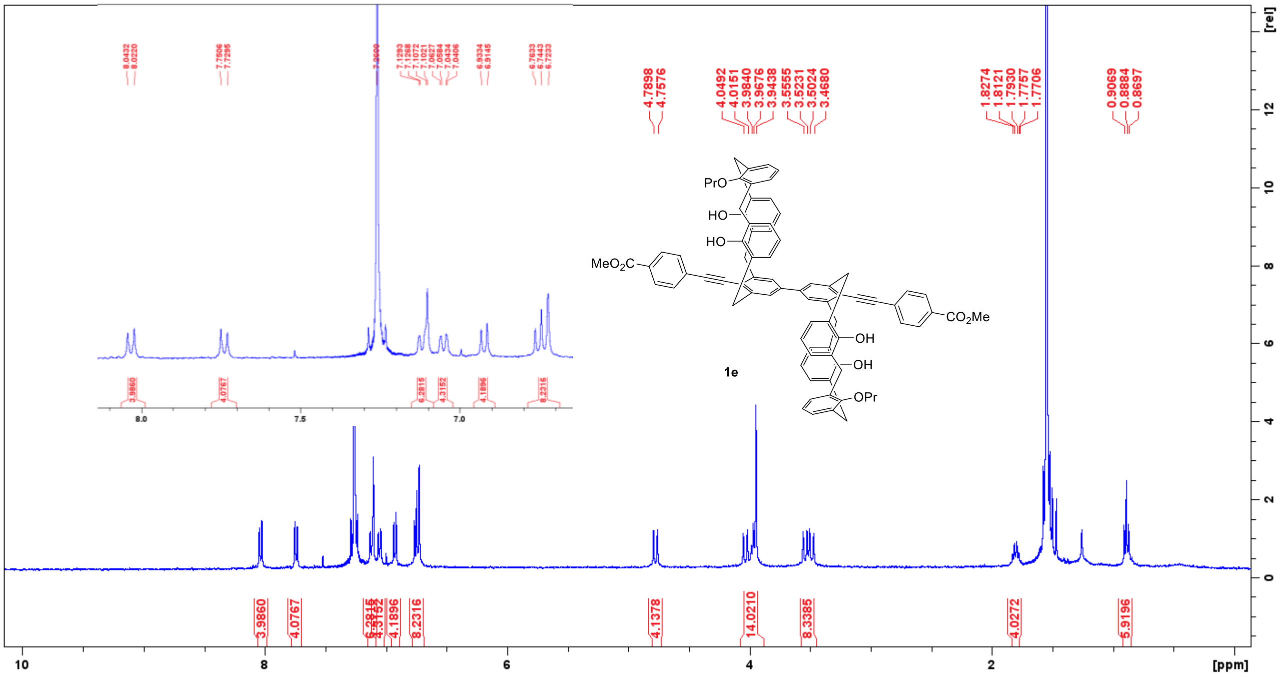

Fig. S11 ^{1}H NMR (400 MHz) spectrum of 1e in $CDCl_3$

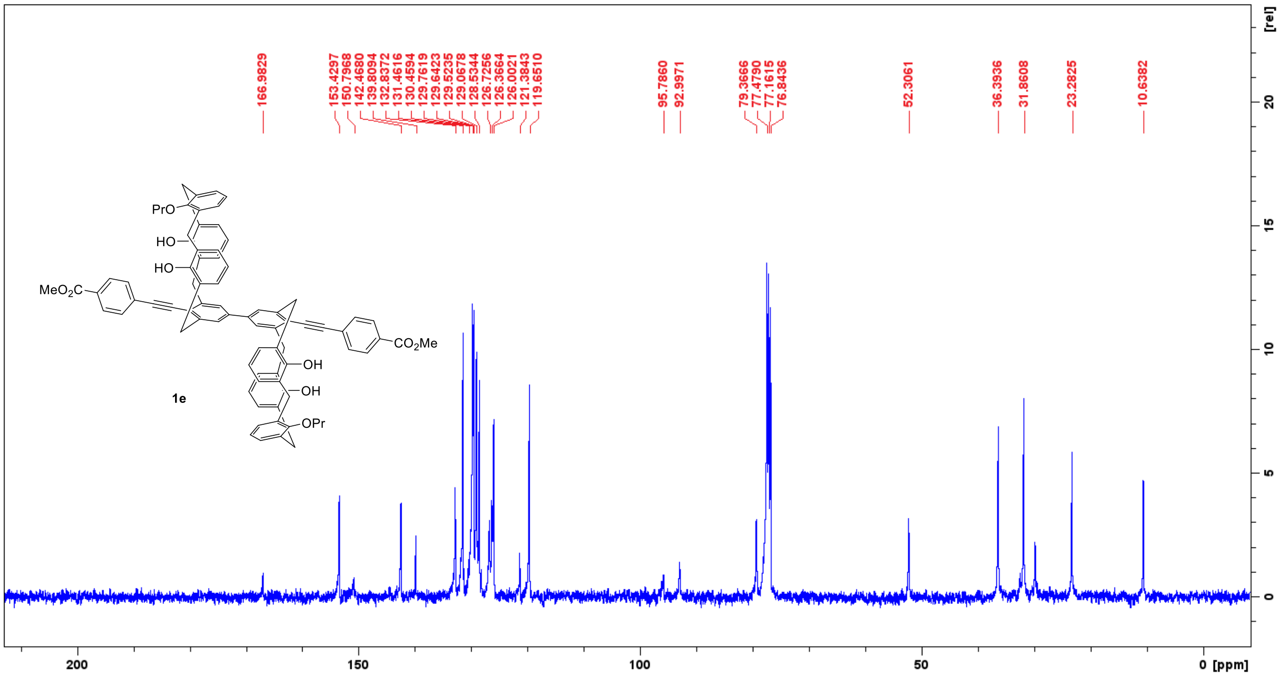

Fig. S12 ^{13}C{^{1}H} NMR (101 MHz) spectrum of 1e in $CDCl_3$

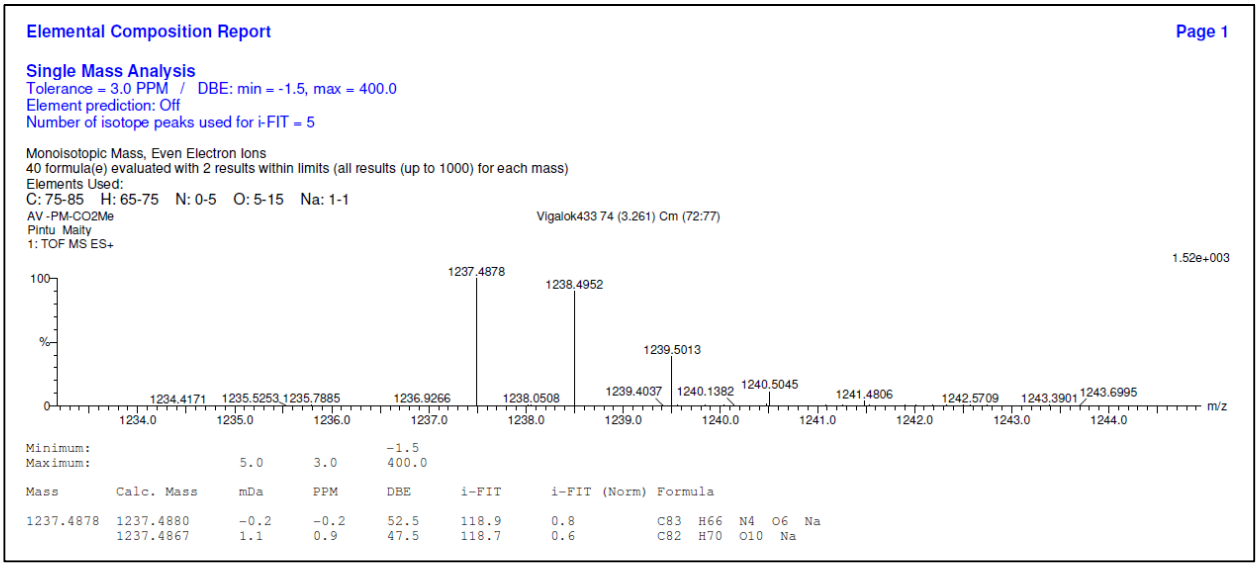

Fig. S13 Mass spectrum of 1e

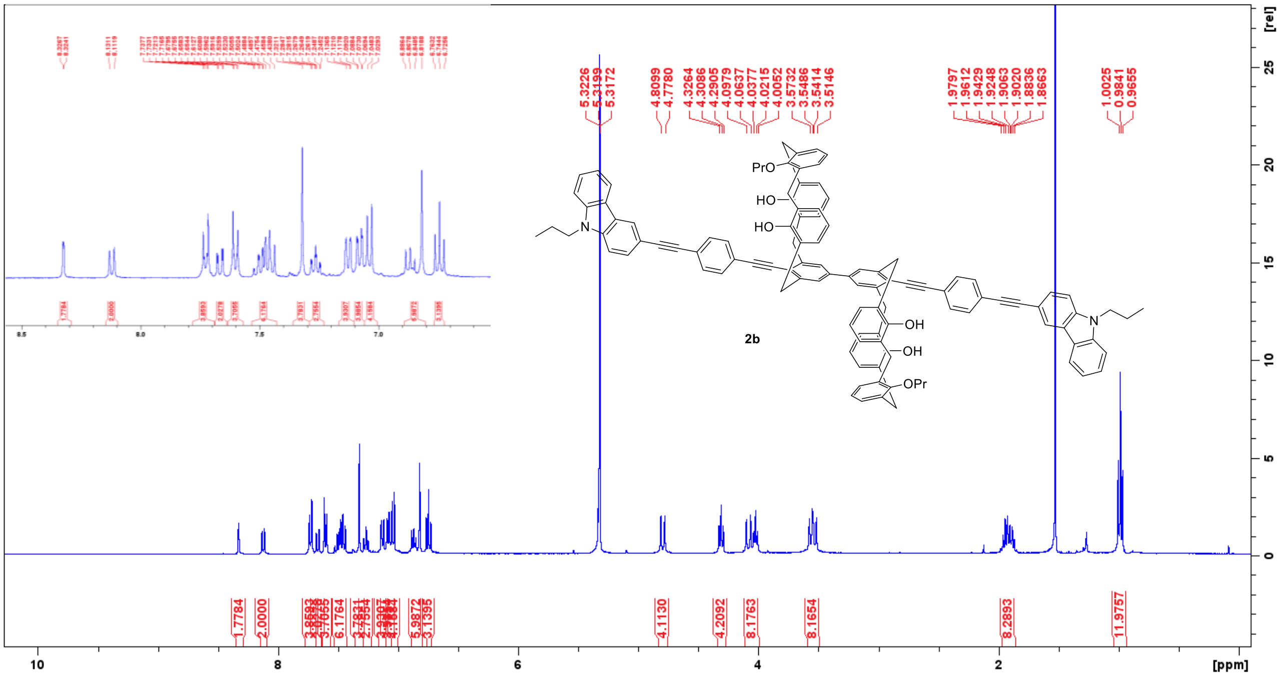

Fig. S14 ^{1}H NMR (400 MHz) spectrum of 2b in CD_2Cl_2

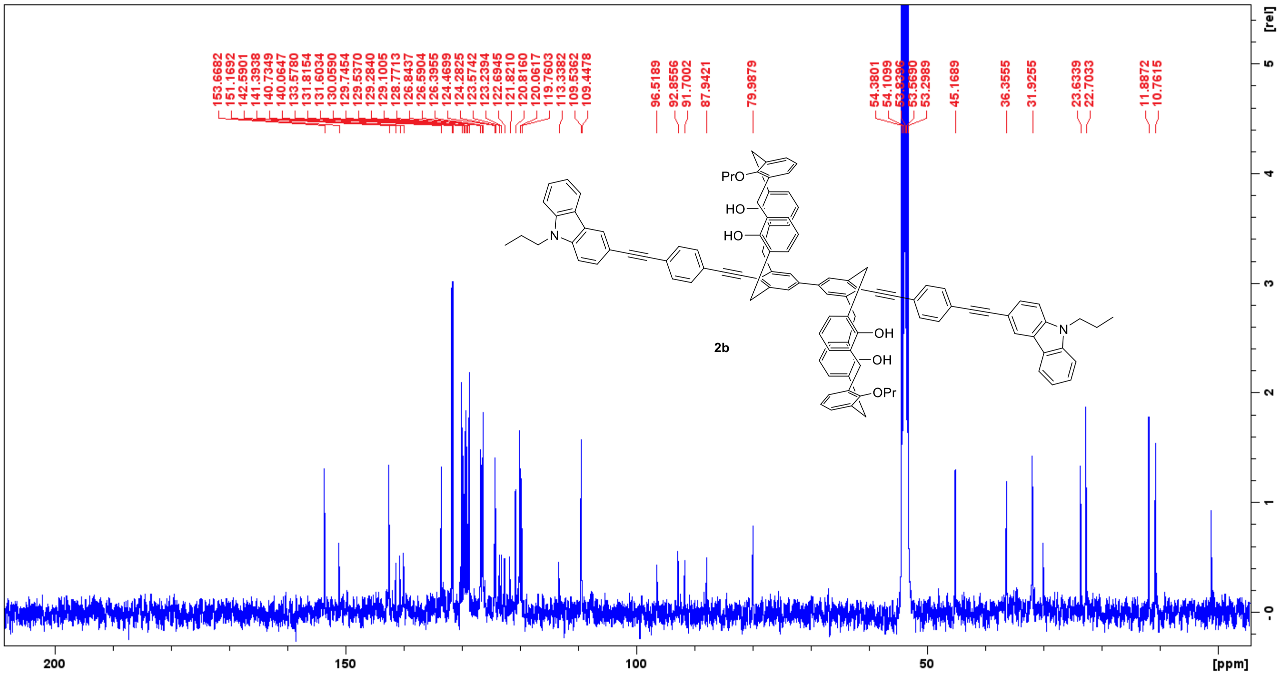

Fig. S15 $^{13}C\{^1H\}$ **NMR (101 MHz) spectrum of 2b in** CD_2Cl_2

Elemental Composition Report **Page 1**

Single Mass Analysis
Tolerance = 2.0 PPM / DBE: min = -1.5, max = 200.0
Element prediction: Off
Number of isotope peaks used for i-FIT = 3

Monoisotopic Mass, Odd and Even Electron Ions
211 formula(e) evaluated with 8 results within limits (up to 4 best isotopic matches for each mass)
Elements Used:
C: 100-120 H: 80-100 N: 0-5 O: 0-10 Na: 0-1
NGMF017 Michal Farber
Vigalok4086 10 (0.196) Cm (10:14) 1: TOF MS ES+ 8.51e+005

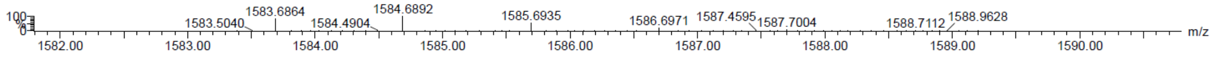

Minimum:				-1.5				
Maximum:		5.0	2.0	200.0				
Mass	Calc. Mass	mDa	PPM	DBE	i-FIT	Norm	Conf(%)	Formula
1583.6864	1583.6850	1.4	0.9	66.0	425.9	1.248	28.72	C111 H93 N O9
	1583.6853	1.1	0.7	67.5	425.9	1.253	28.55	C112 H92 N2 O6 Na
	1583.6864	0.0	0.0	71.0	425.9	1.305	27.13	C112 H89 N5 O5
	1583.6840	2.4	1.5	68.0	426.5	1.858	15.60	C110 H90 N5 O5 Na

Fig. S16 Mass spectrum of 2b

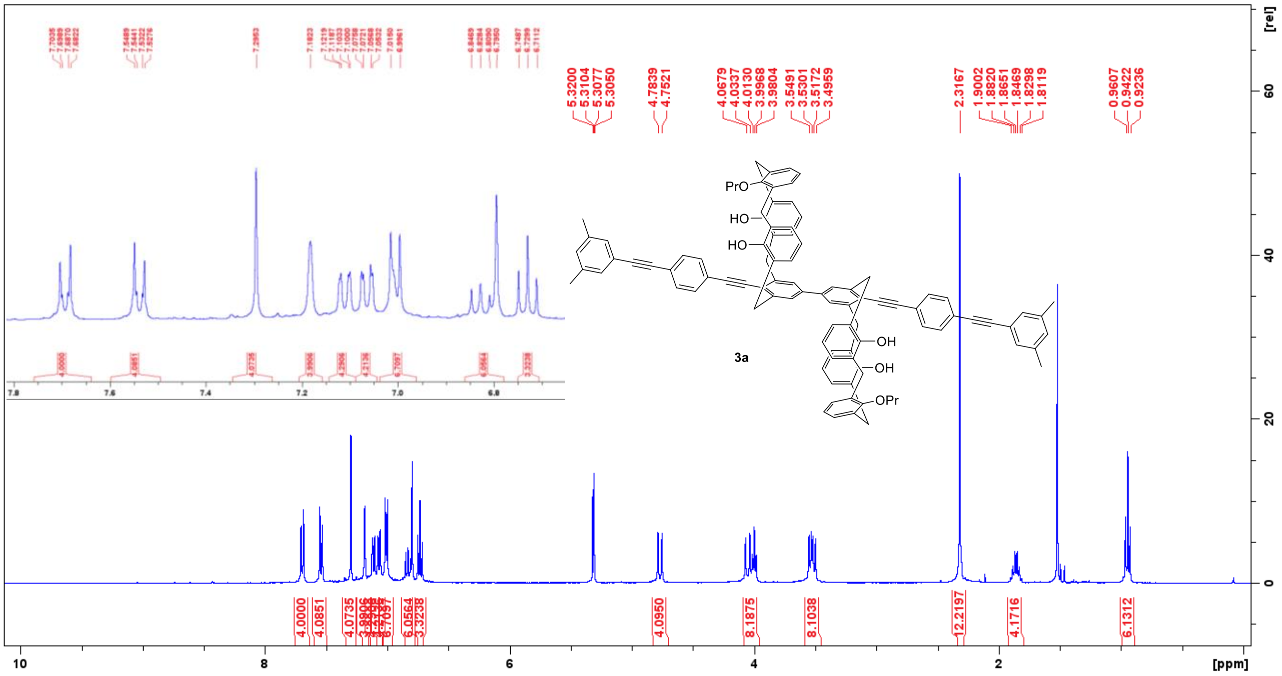

Fig. S17 ^{1}H NMR (400 MHz) spectrum of 3a in CD_2Cl_2

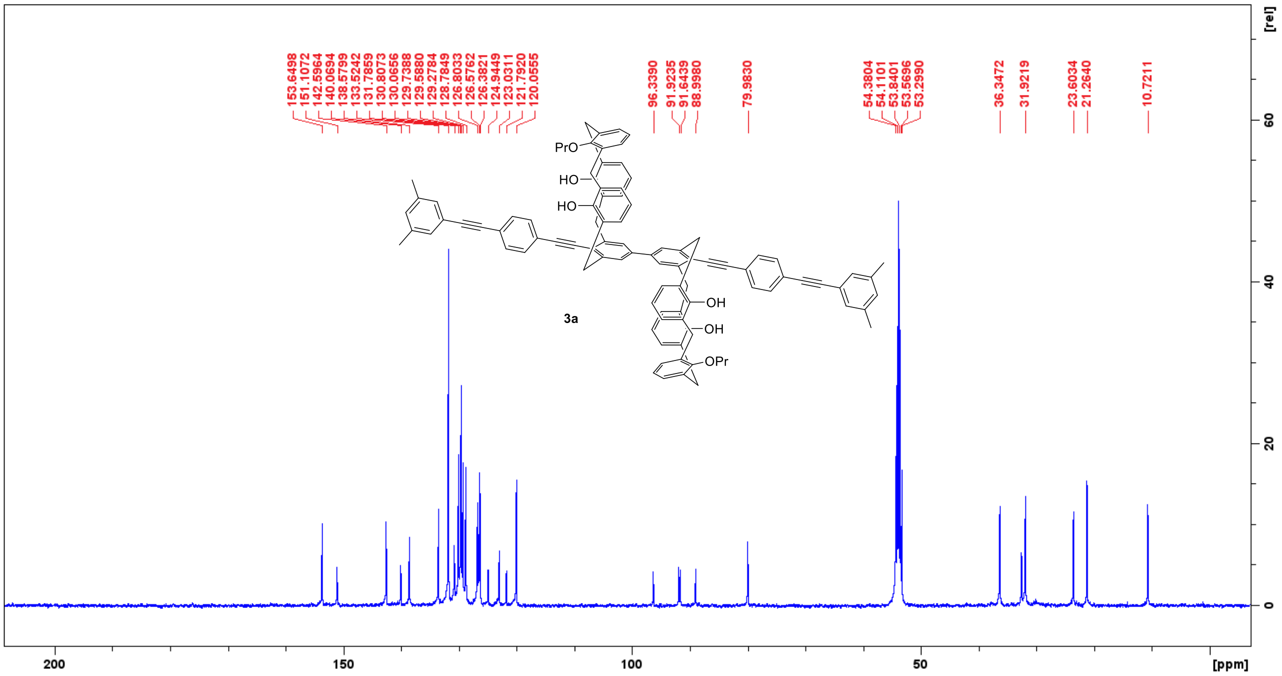

Fig. S18 $^{13}C\{^{1}H\}$ NMR (101 MHz) spectrum of 3a in CD_2Cl_2

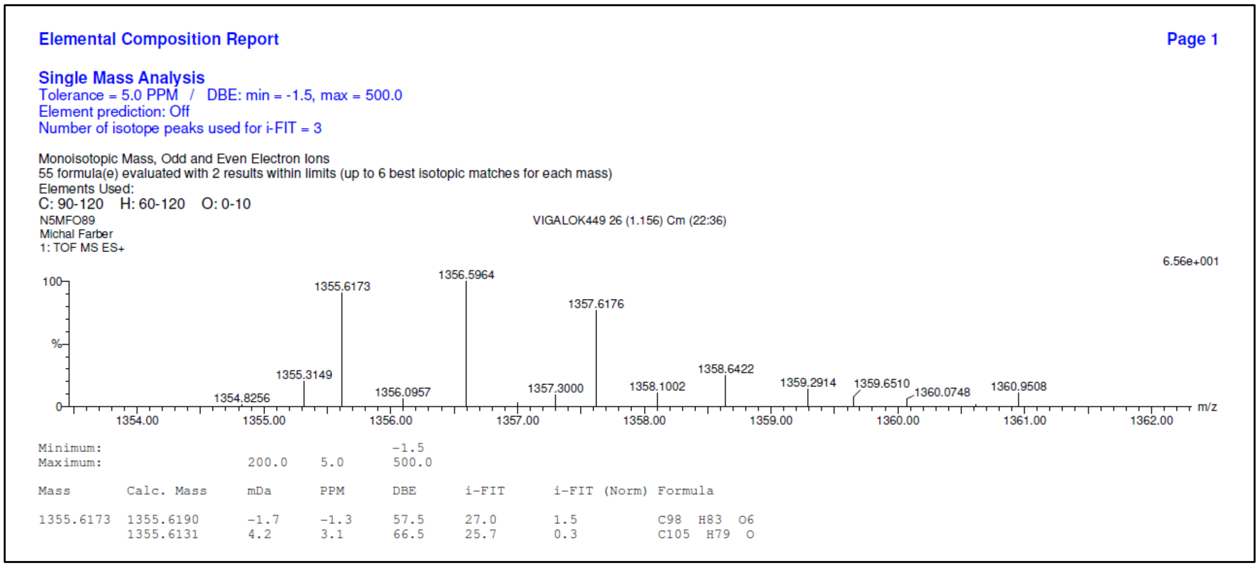

Fig. S19 Mass spectrum of 3a

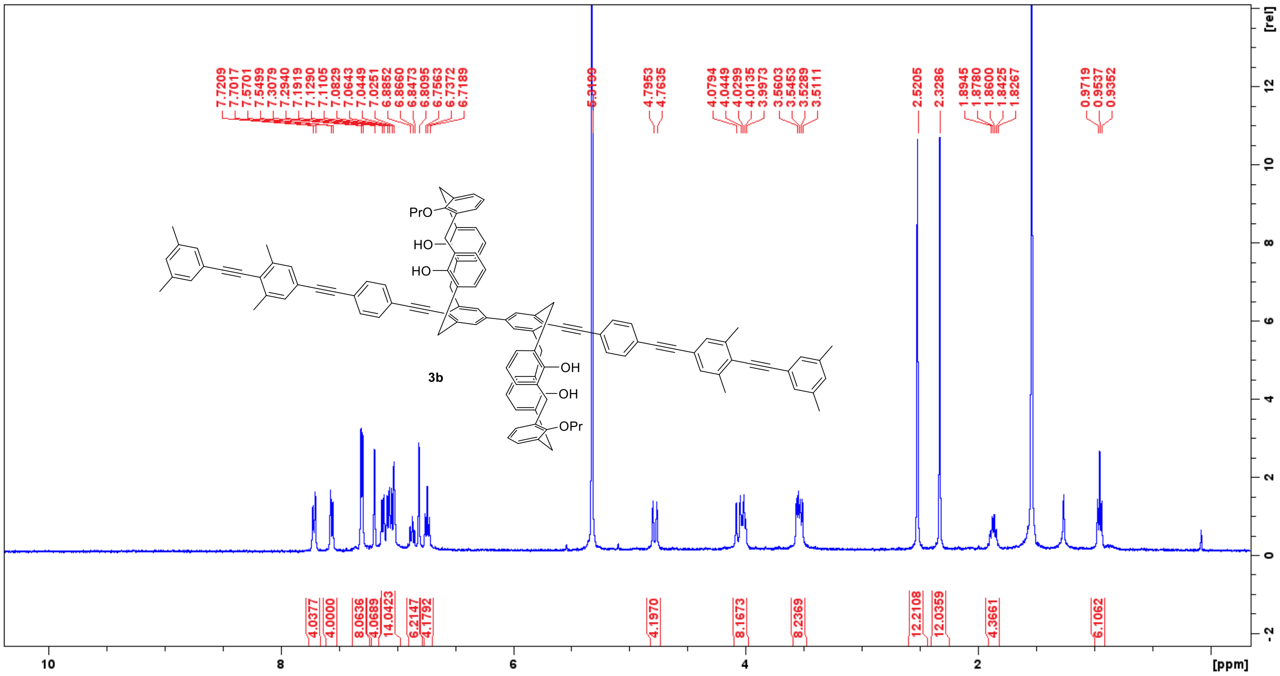

Fig. S19 ^{1}H NMR (400 MHz) spectrum of 3b in CD_2Cl_2

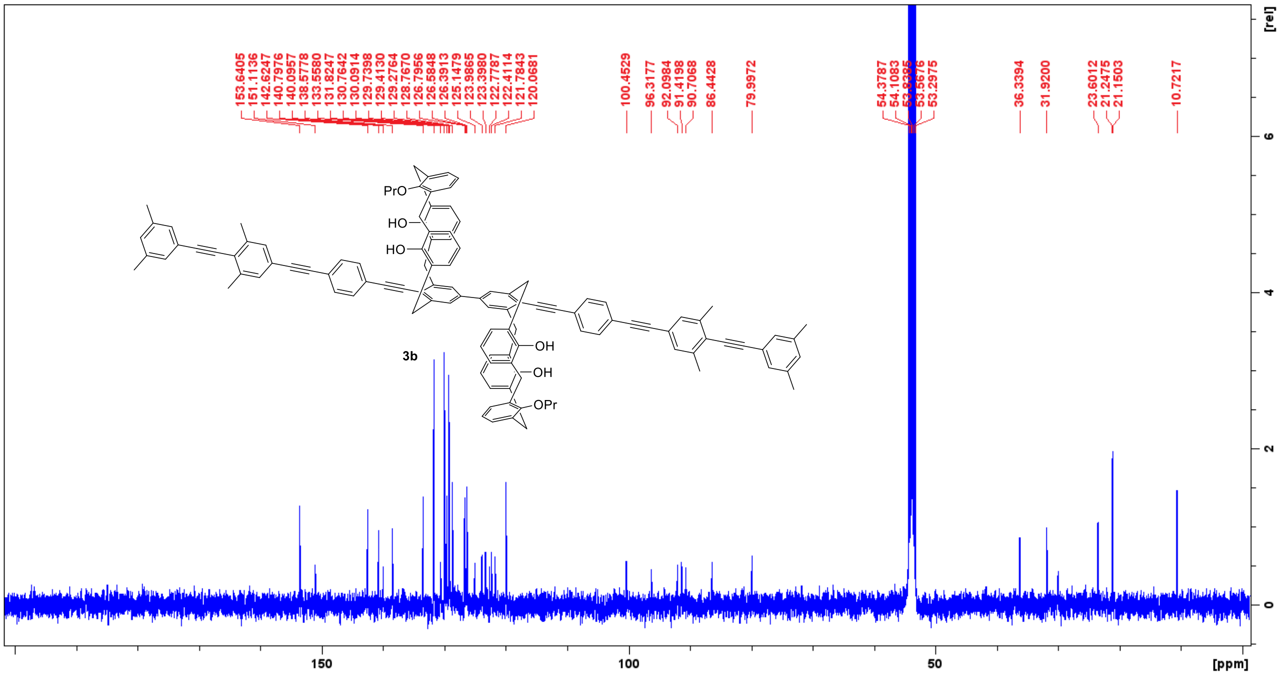

Fig. S20 $^{13}C\{^{1}H\}$ NMR (101 MHz) spectrum of 3b in CD_2Cl_2

Elemental Composition Report

Page 1

Single Mass Analysis

Tolerance = 2.0 PPM / DBE: min = -1.5, max = 200.0
Element prediction: Off
Number of isotope peaks used for i-FIT = 3

Monoisotopic Mass, Even Electron Ions
156 formula(e) evaluated with 4 results within limits (up to 4 best isotopic matches for each mass)
Elements Used:
C: 100-120 H: 80-100 N: 0-5 O: 0-10 Na: 0-1

NGMF043
Vigalok4087 9 (0.179) Cm (9:22)

Michal Farber

1: TOF MS ES+
2.28e+006

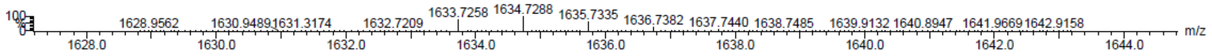

Mass	Calc. Mass	mDa	PPM	DBE	i-FIT	Norm	Conf(%)	Formula
Minimum:				-1.5				
Maximum:		5.0	2.0	200.0				
1633.7258	1633.7245	1.3	0.8	68.5	538.5	0.882	41.40	C115 H97 N2 O8
	1633.7261	-0.3	-0.2	69.5	538.6	0.975	37.71	C118 H98 O6 Na
	1633.7274	-1.6	-1.0	74.5	539.5	1.859	15.58	C119 H94 N4 O2 Na
	1633.7285	-2.7	-1.7	72.5	540.6	2.936	5.31	C120 H97 O6

Fig. S21 Mass spectrum of 3b

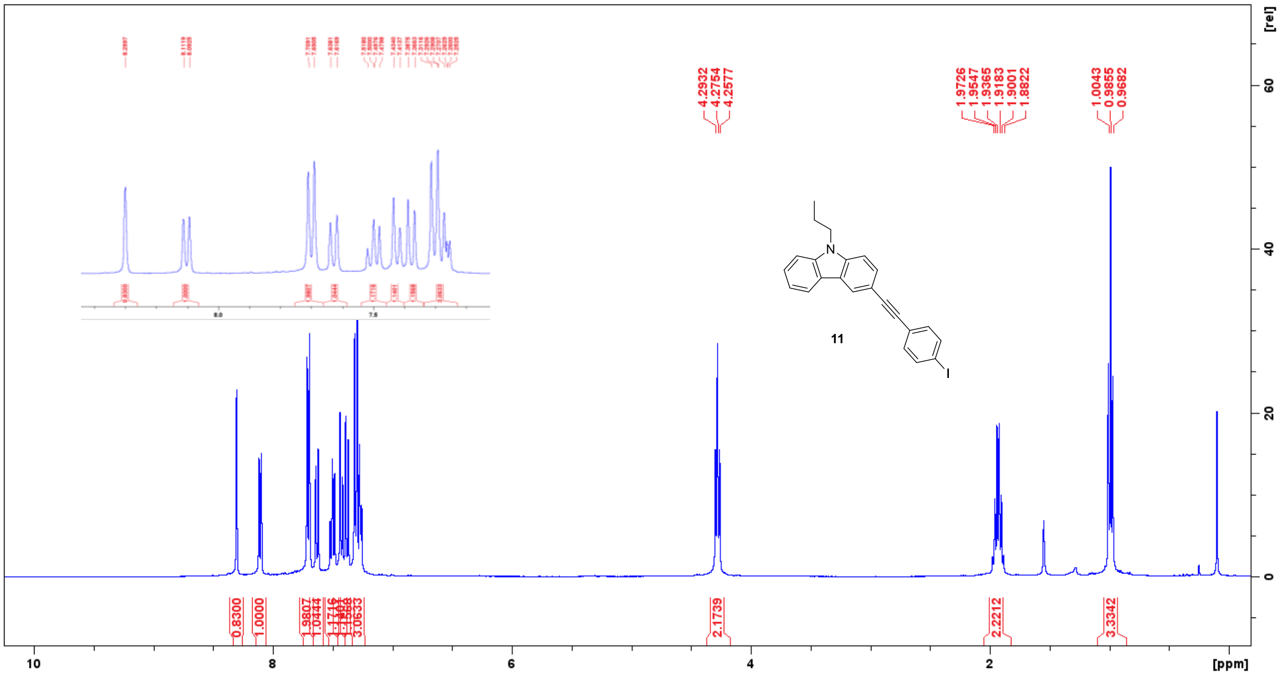

Fig. S22 ^{1}H NMR (400 MHz) spectrum of 11 in $CDCl_3$

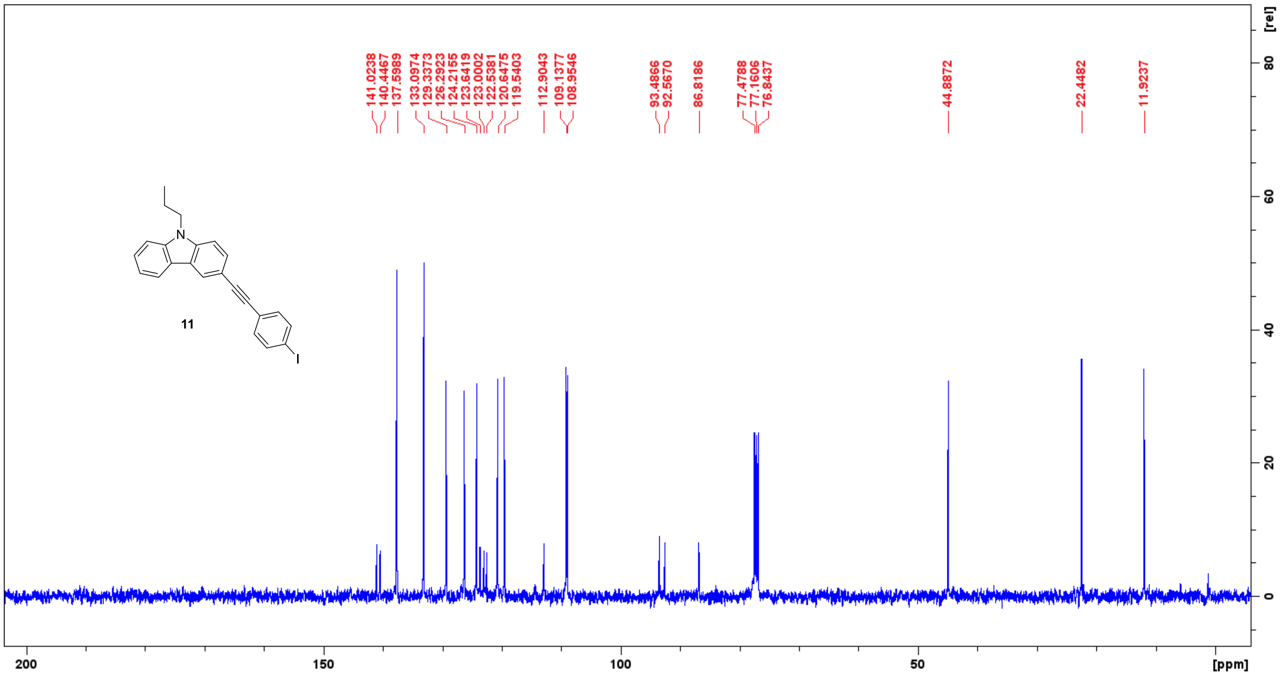

Fig. S23 $^{13}C\{^1H\}$ NMR (101 MHz) spectrum of 11 in $CDCl_3$

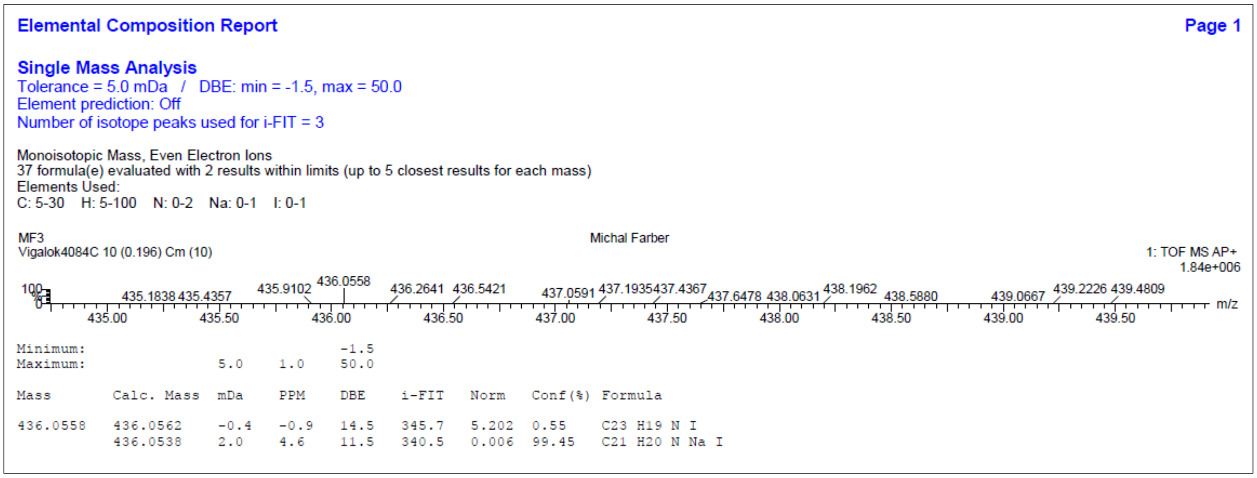

Elemental Composition Report Page 1

Single Mass Analysis
Tolerance = 5.0 mDa / DBE: min = -1.5, max = 50.0
Element prediction: Off
Number of isotope peaks used for i-FIT = 3

Monoisotopic Mass, Even Electron Ions
37 formula(e) evaluated with 2 results within limits (up to 5 closest results for each mass)
Elements Used:
C: 5-30 H: 5-100 N: 0-2 Na: 0-1 I: 0-1

Minimum:				-1.5				
Maximum:		5.0	1.0	50.0				
Mass	Calc. Mass	mDa	PPM	DBE	i-FIT	Norm	Conf(%)	Formula
436.0558	436.0562	-0.4	-0.9	14.5	345.7	5.202	0.55	C23 H19 N I
	436.0538	2.0	4.6	11.5	340.5	0.006	99.45	C21 H20 N Na I

Fig. S24 Mass spectrum of 11

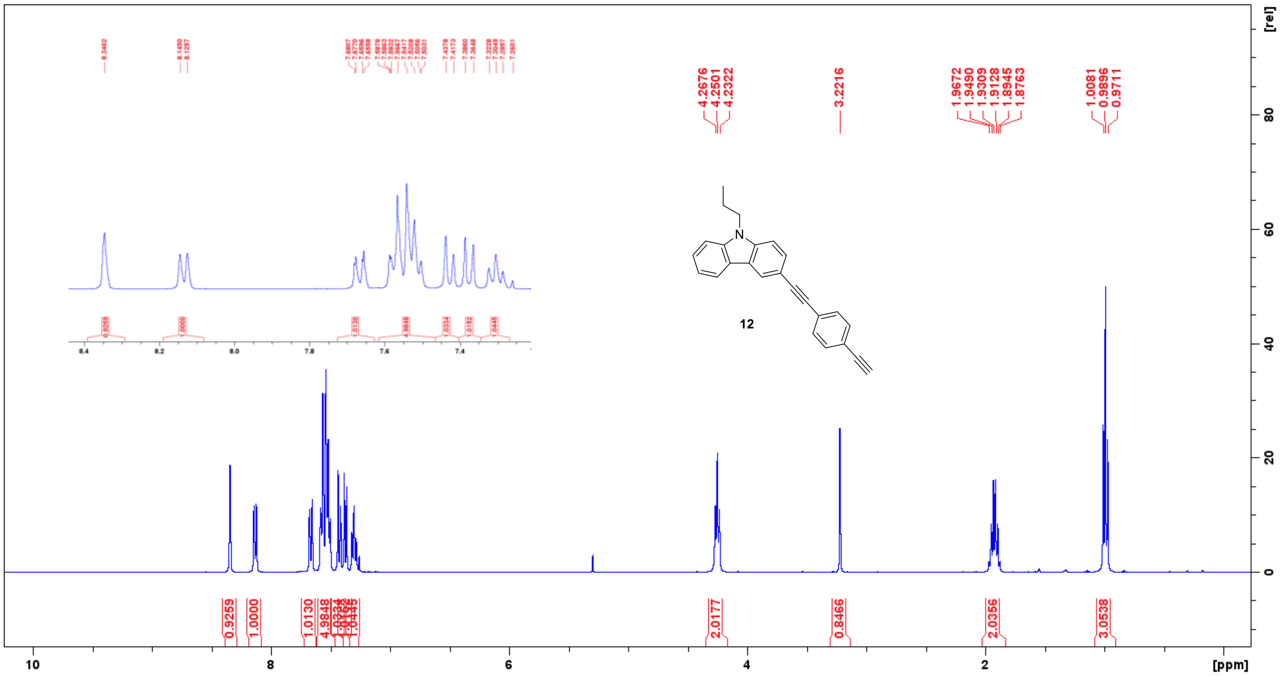

Fig. S25 ^{1}H NMR (400 MHz) spectrum of 12 in $CDCl_3$

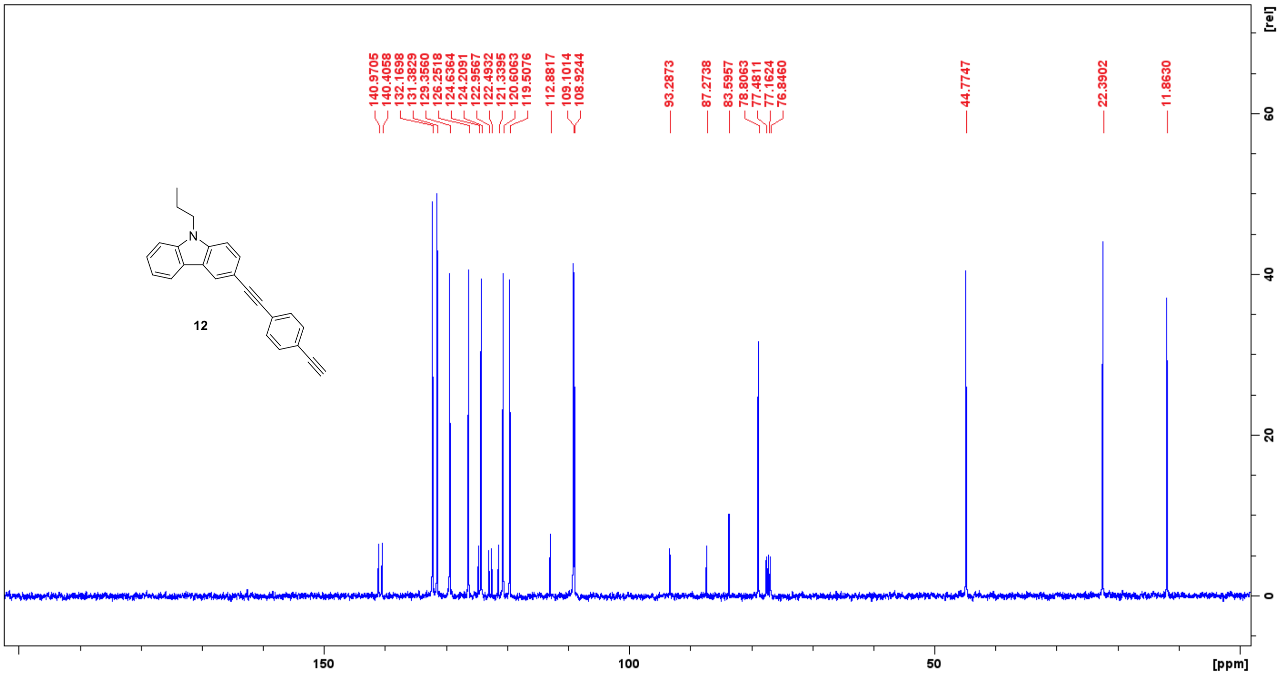

Fig. S26 $^{13}C\{^{1}H\}$ **NMR (101 MHz) spectrum of 12 in** $CDCl_3$

Elemental Composition Report **Page 1**

Single Mass Analysis
Tolerance = 5.0 mDa / DBE: min = -1.5, max = 50.0
Element prediction: Off
Number of isotope peaks used for i-FIT = 3

Monoisotopic Mass, Even Electron Ions
42 formula(e) evaluated with 2 results within limits (up to 5 closest results for each mass)
Elements Used:
C: 5-30 H: 5-100 N: 0-2 Na: 0-1 I: 0-1

MF4 Michal Farber
Vigalok4085C 9 (0.171) Cm (9) 1: TOF MS AP+
5.61e+006

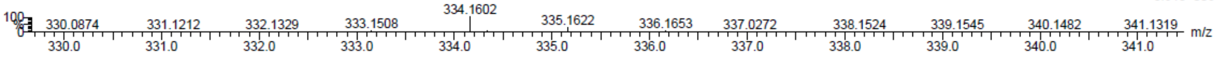

Minimum:				-1.5				
Maximum:		5.0	1.0	50.0				
Mass	Calc. Mass	mDa	PPM	DBE	i-FIT	Norm	Conf(%)	Formula
334.1602	334.1596	0.6	1.8	16.5	456.5	0.102	90.33	C25 H20 N
	334.1572	3.0	9.0	13.5	458.7	2.336	9.67	C23 H21 N Na

Fig. S27 Mass spectrum of 12

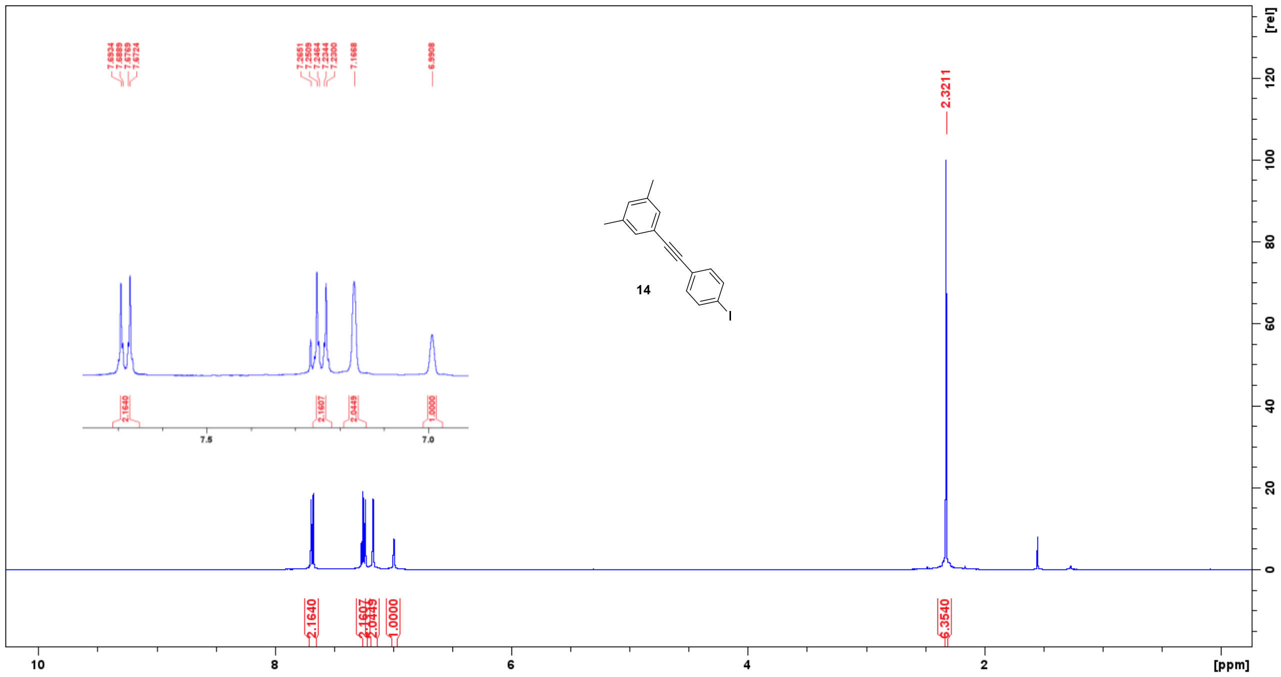

Fig. S28 ^{1}H NMR (400 MHz) spectrum of 14 in $CDCl_3$

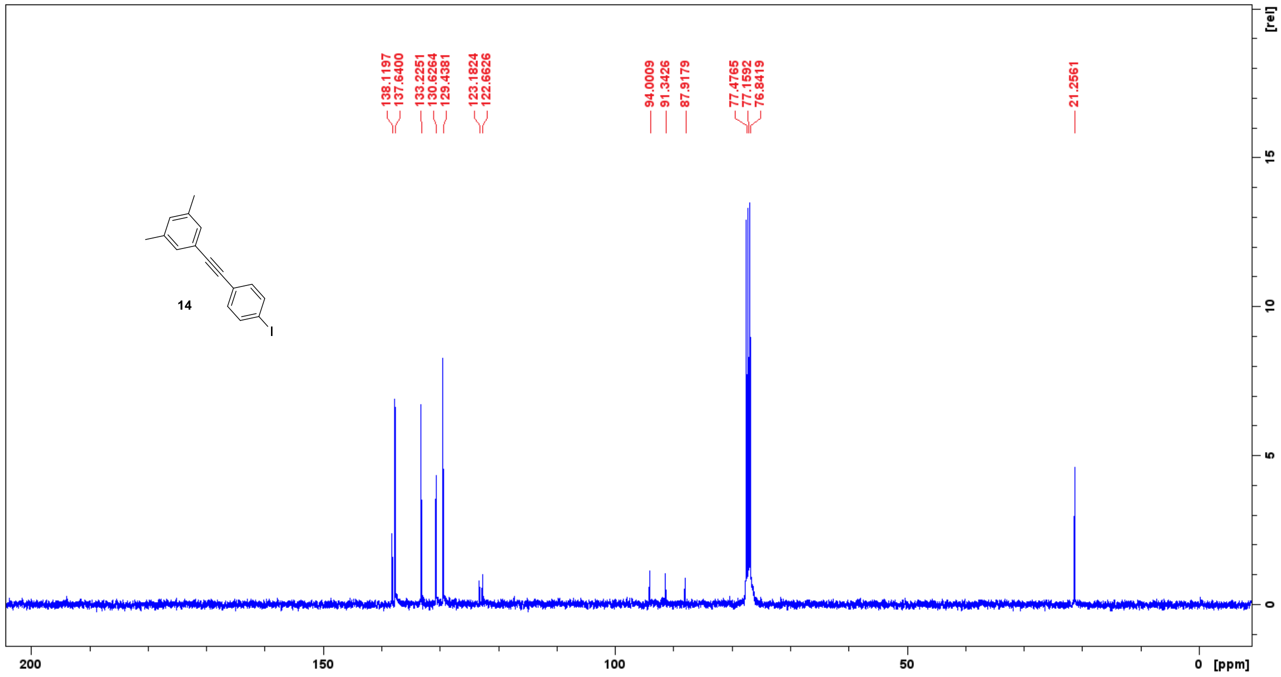

Fig. S29 $^{13}C\{^{1}H\}$ NMR (101 MHz) spectrum of 14 in $CDCl_3$

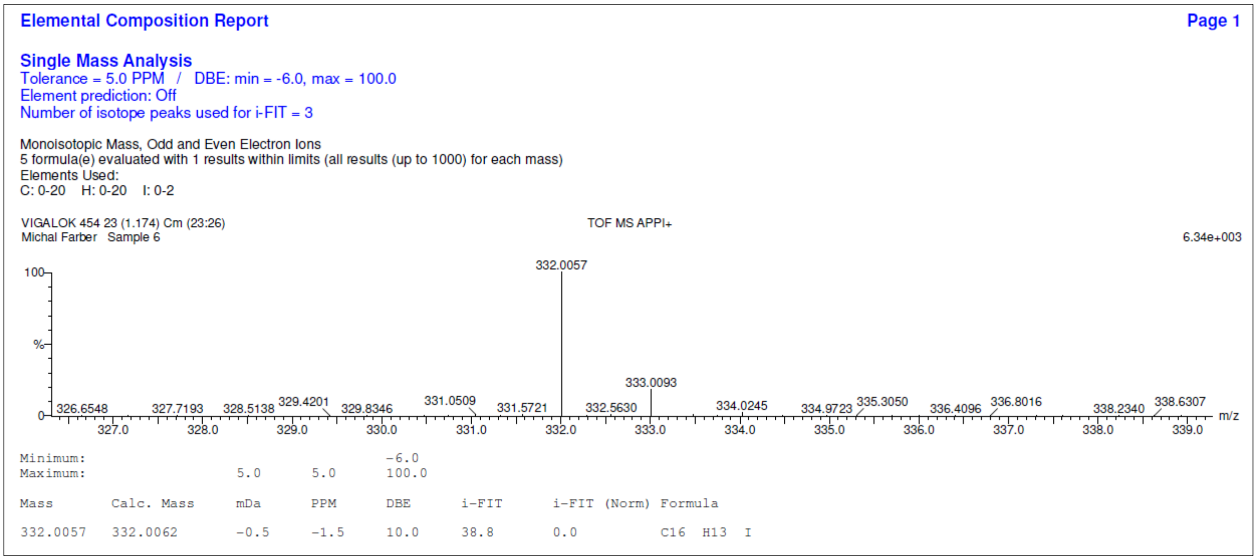

Fig. S30 Mass spectrum of 14

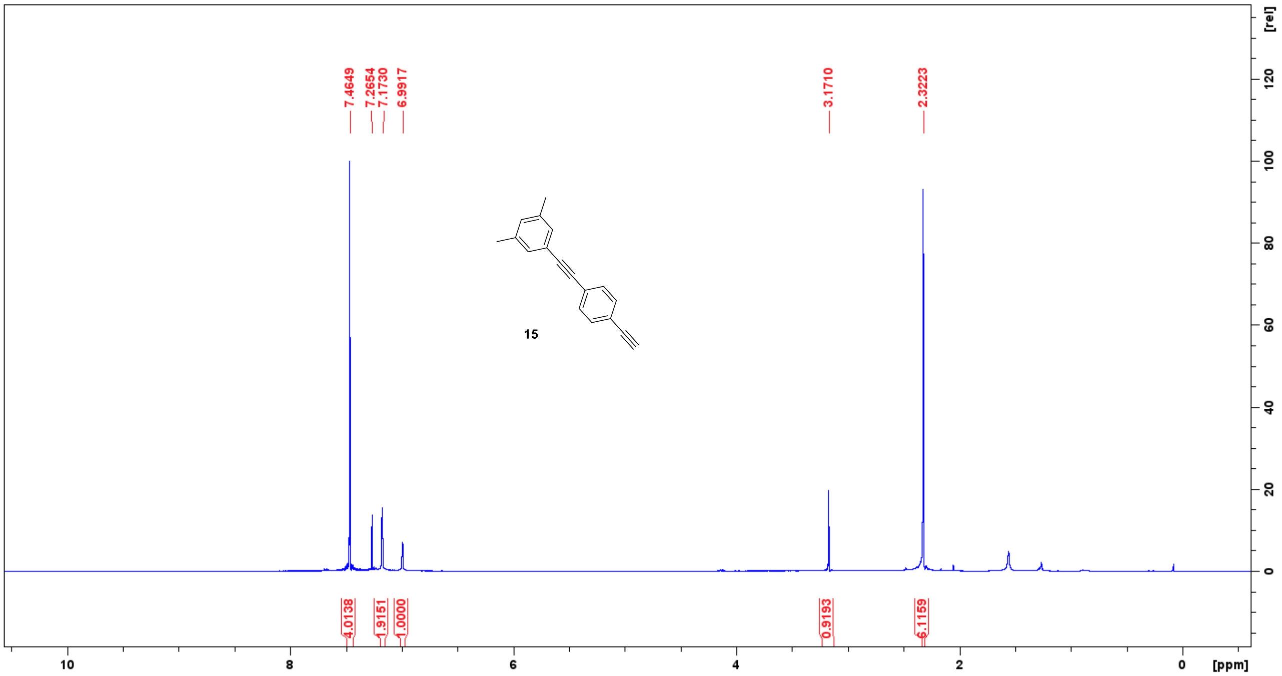

Fig. S31 ^{1}H NMR (400 MHz) spectrum of 15 in $CDCl_3$

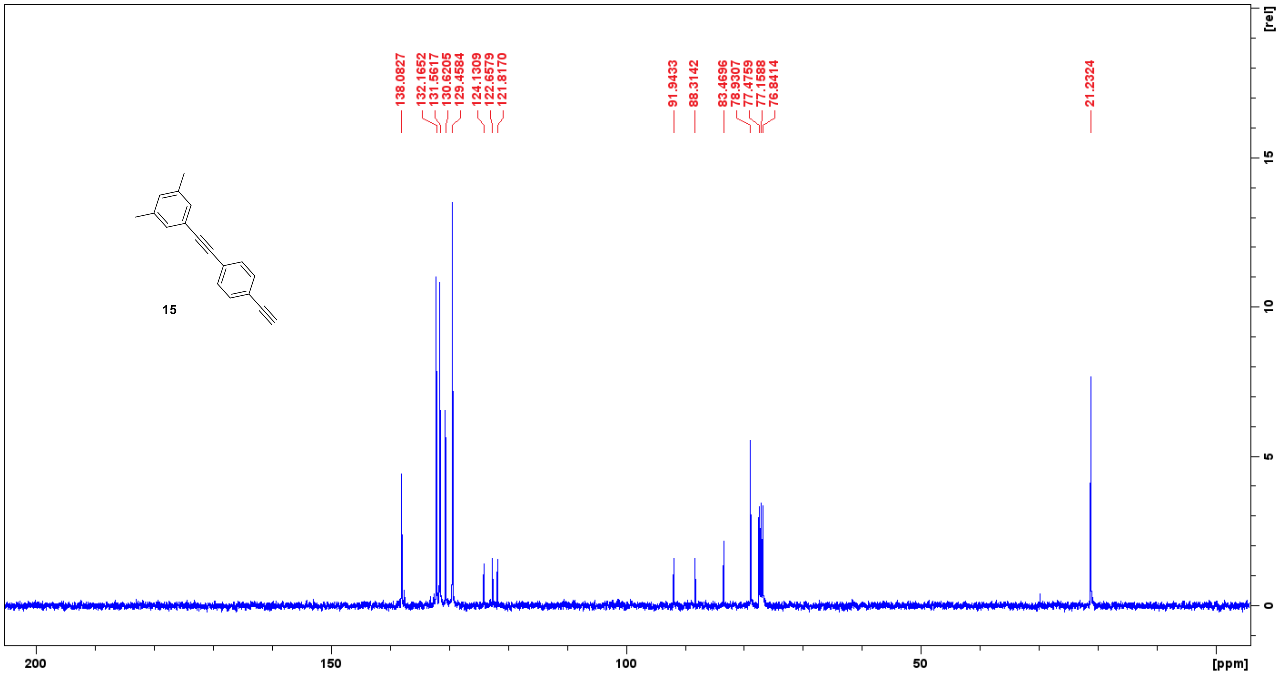

Fig. S32 $^{13}C\{^{1}H\}$ NMR (101 MHz) spectrum of 15 in $CDCl_3$

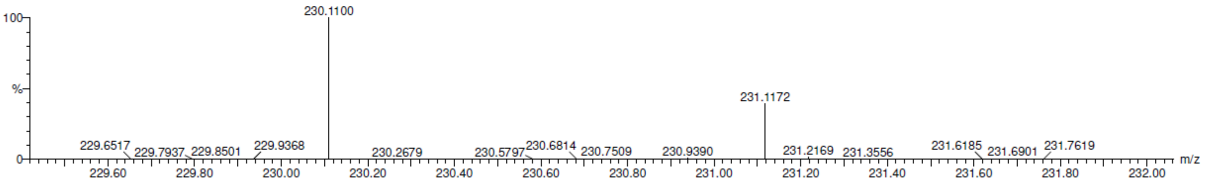

Elemental Composition Report Page 1

Single Mass Analysis
Tolerance = 10.0 PPM / DBE: min = -6.0, max = 100.0
Element prediction: Off
Number of isotope peaks used for i-FIT = 5

Monoisotopic Mass, Odd and Even Electron Ions
2 formula(e) evaluated with 1 results within limits (all results (up to 1000) for each mass)
Elements Used:
C: 10-20 H: 0-20
VIGALOK459D 117 (5.904) Cm (115:117) TOF MS AP+
sample7 Michal Farber 5.71e+004

Minimum:				-6.0			
Maximum:		5.0	10.0	100.0			
Mass	Calc. Mass	mDa	PPM	DBE	i-FIT	i-FIT (Norm)	Formula
230.1100	230.1096	0.4	1.7	12.0	310.6	0.0	C18 H14

Fig. S33 Mass spectrum of 15

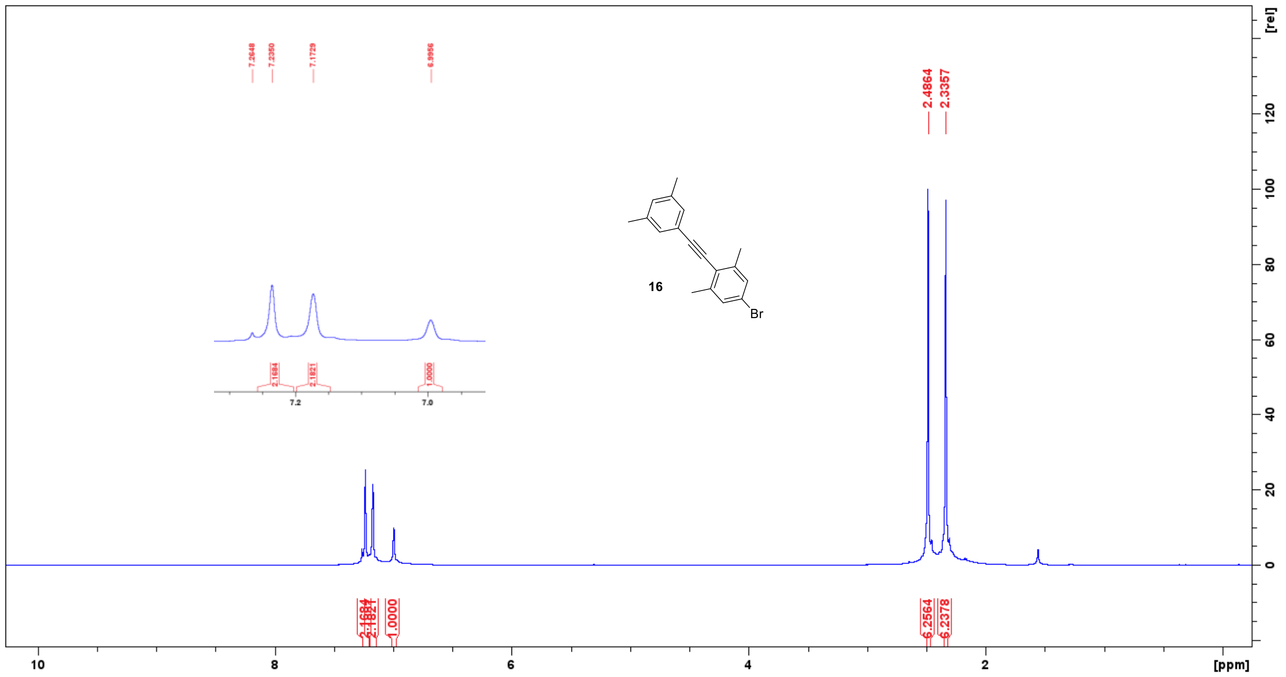

Fig. S34 ^{1}H NMR (400 MHz) spectrum of 16 in $CDCl_3$

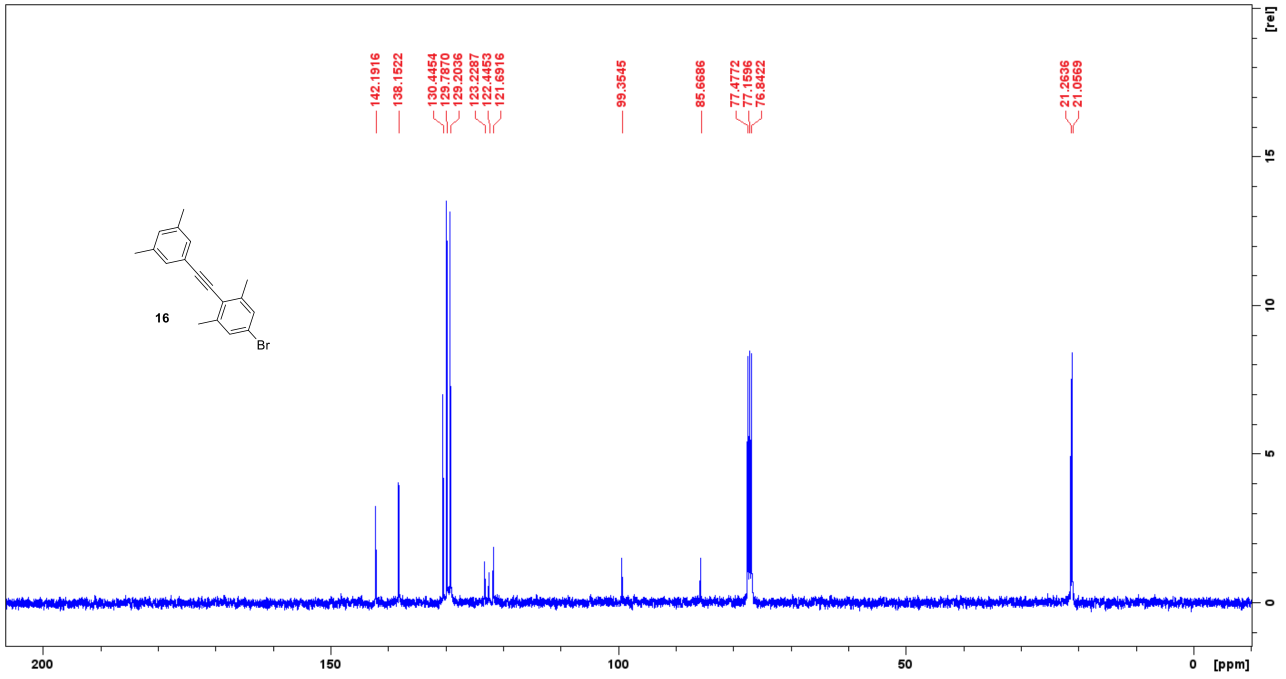

Fig. S35 $^{13}C\{^{1}H\}$ NMR (101 MHz) spectrum of 16 in $CDCl_3$

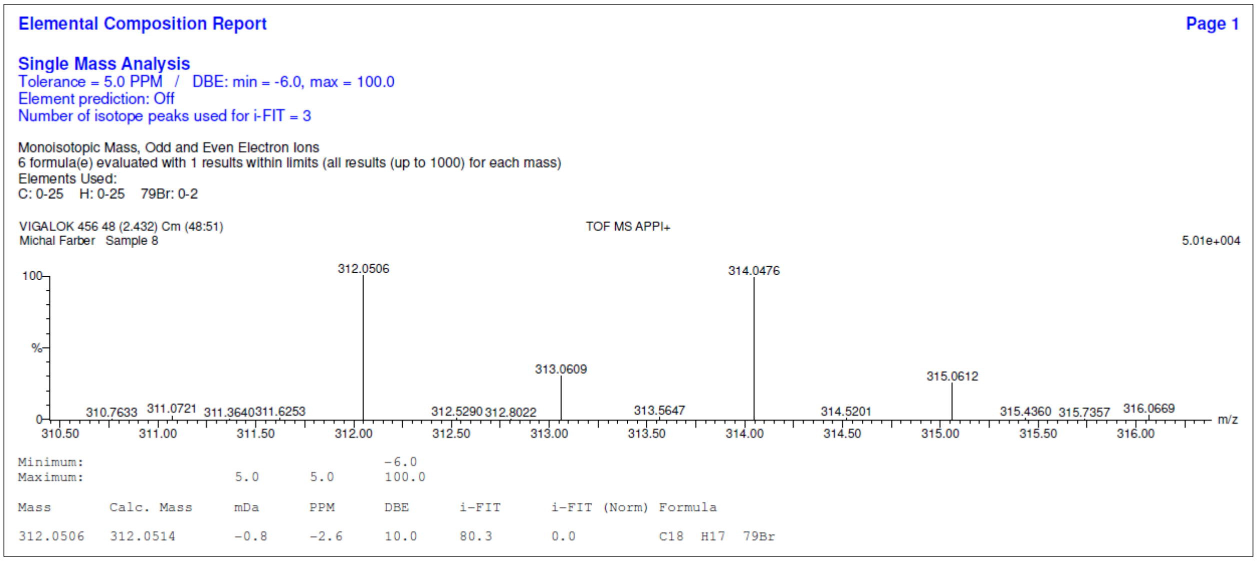

Fig. S36 Mass spectrum of 16

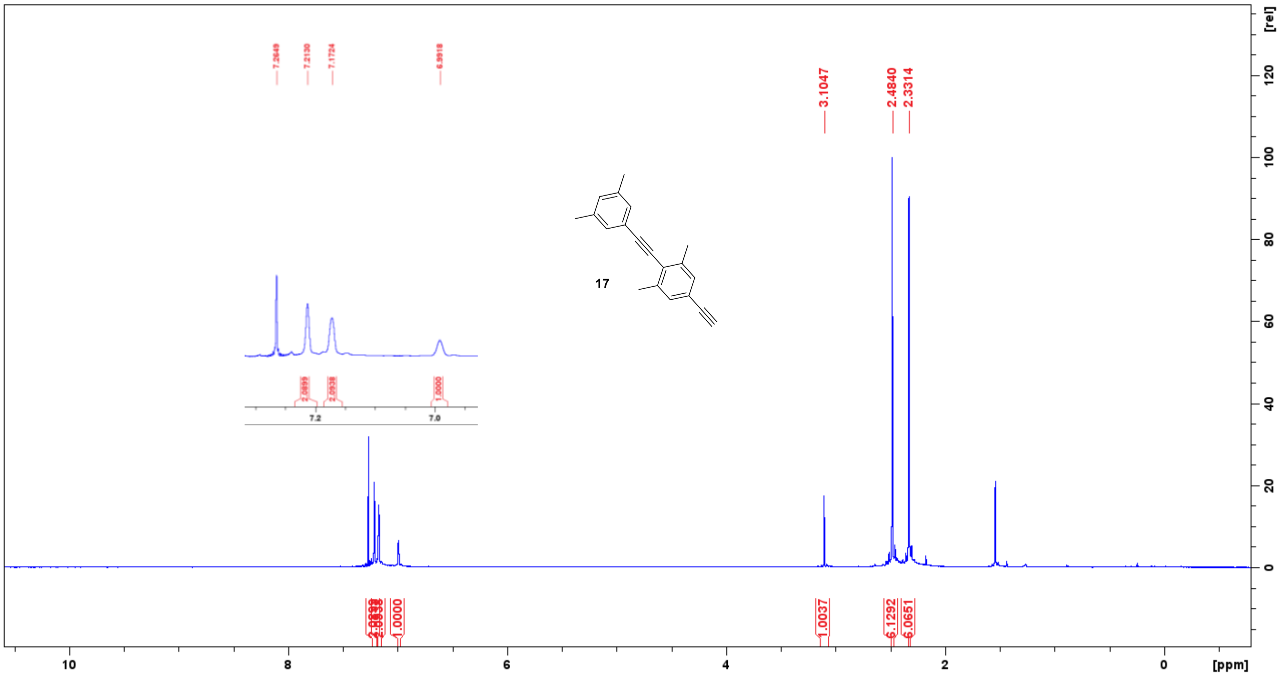

Fig. S37 ^{1}H NMR (400 MHz) spectrum of 17 in $CDCl_3$

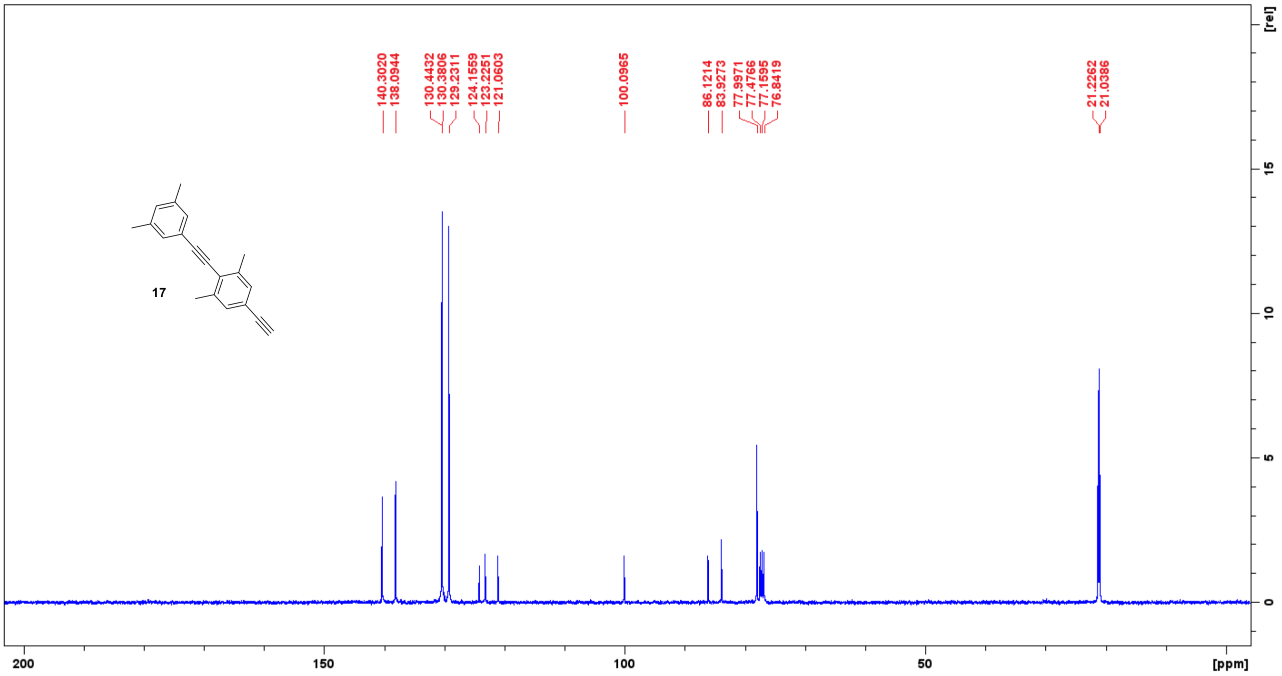

Fig. S38 $^{13}C\{^{1}H\}$ NMR (101 MHz) spectrum of 17 in $CDCl_3$

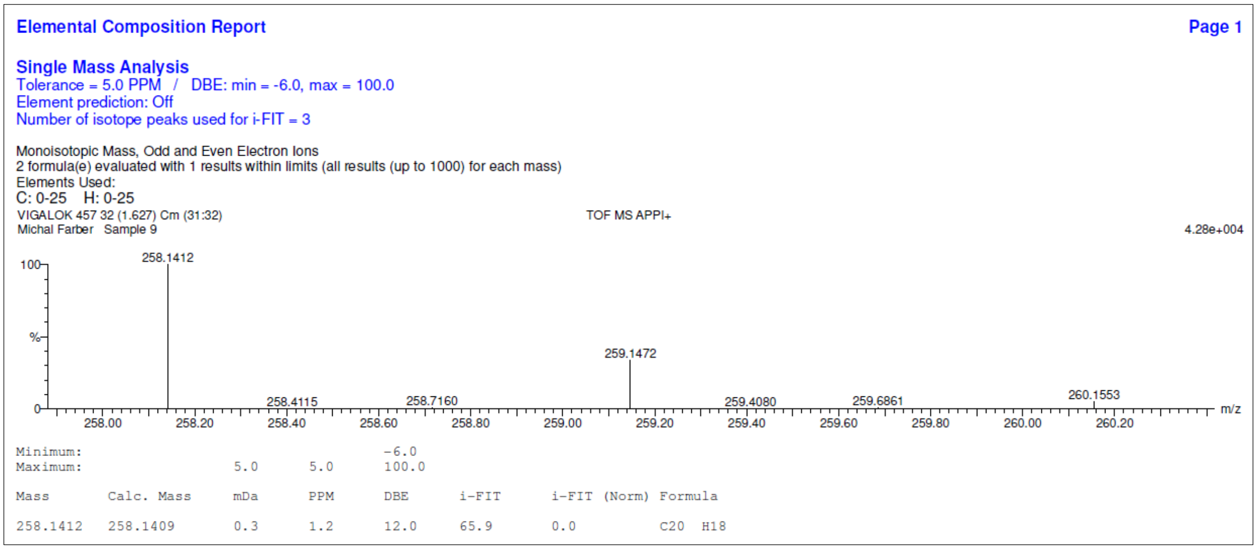

Fig. S39 Mass spectrum of 17

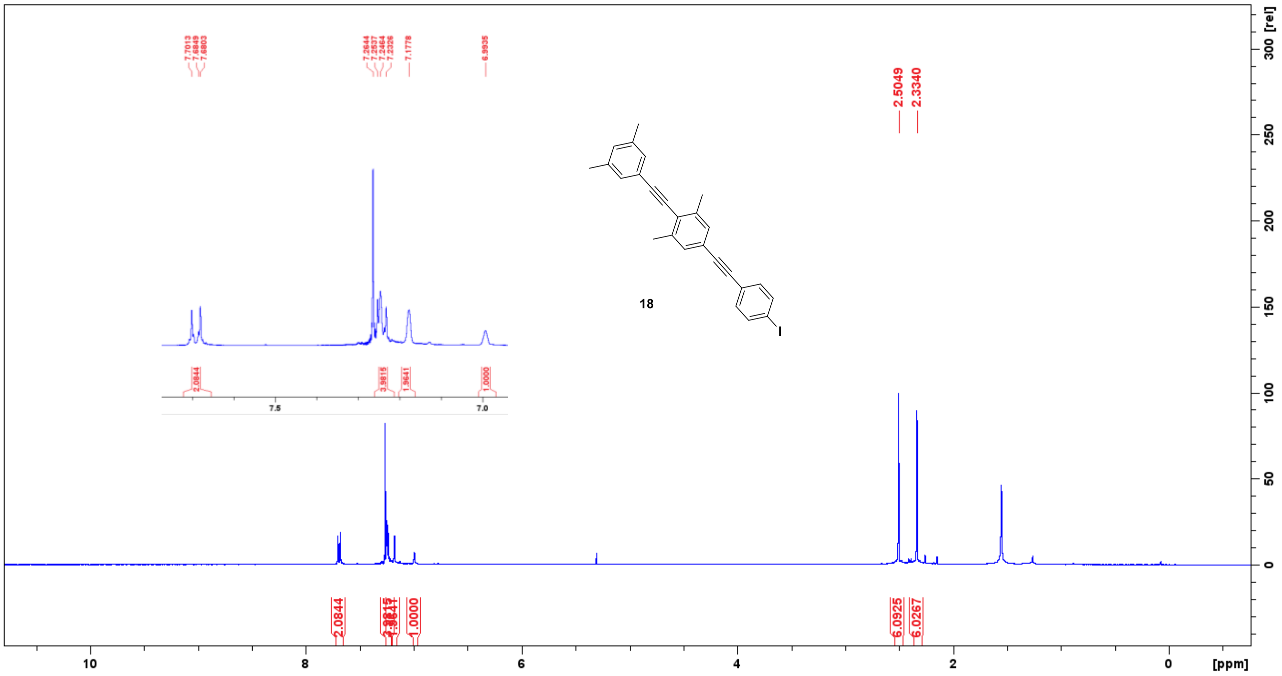

Fig. S40 ^{1}H NMR (400 MHz) spectrum of 18 in $CDCl_3$

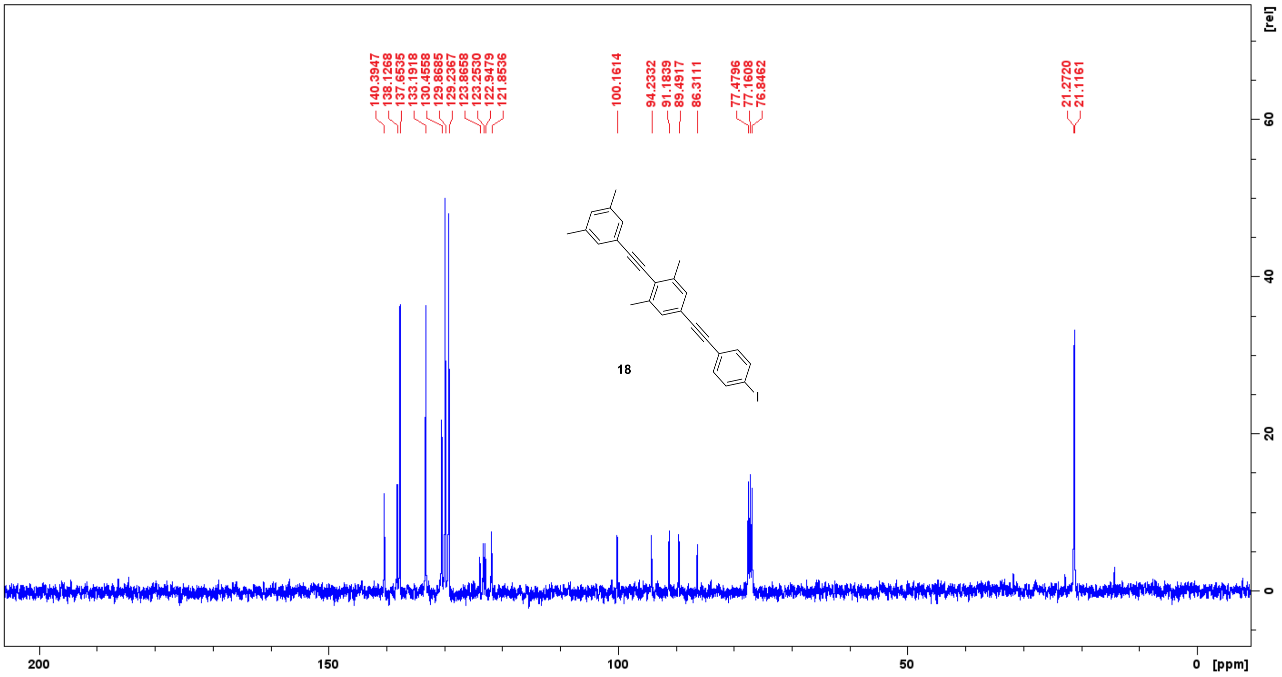

Fig. S41 $^{13}C\{^{1}H\}$ NMR (101 MHz) spectrum of 18 in $CDCl_3$

Elemental Composition Report **Page 1**

Single Mass Analysis
Tolerance = 5.0 PPM / DBE: min = -6.0, max = 100.0
Element prediction: Off
Number of isotope peaks used for i-FIT = 3

Monoisotopic Mass, Odd and Even Electron Ions
11 formula(e) evaluated with 1 results within limits (all results (up to 1000) for each mass)
Elements Used:
C: 0-40 H: 0-40 I: 0-2
VIGALOK 458 177 (8.923) Cm (174:178) TOF MS APPI+
Michal Farber Sample 10 2.64e+005

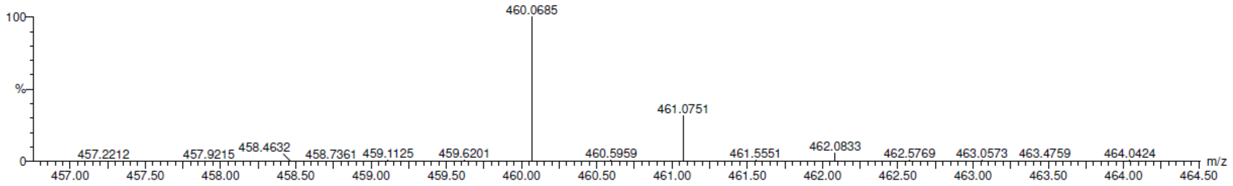

Minimum:				-6.0			
Maximum:		5.0	5.0	100.0			
Mass	Calc. Mass	mDa	PPM	DBE	i-FIT	i-FIT (Norm)	Formula
460.0685	460.0688	-0.3	-0.7	16.0	58.0	0.0	C26 H21 I

Fig. S42 Mass spectrum of 18

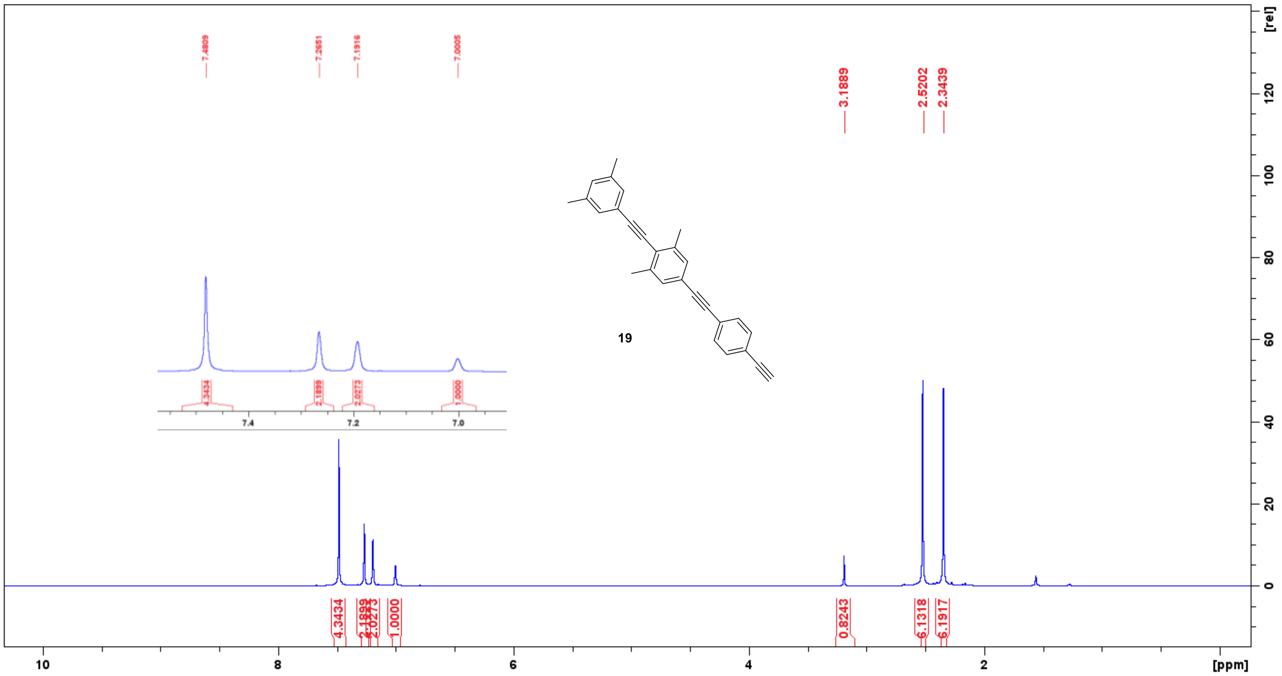

Fig. S43 ^{1}H NMR (400 MHz) spectrum of 19 in $CDCl_3$

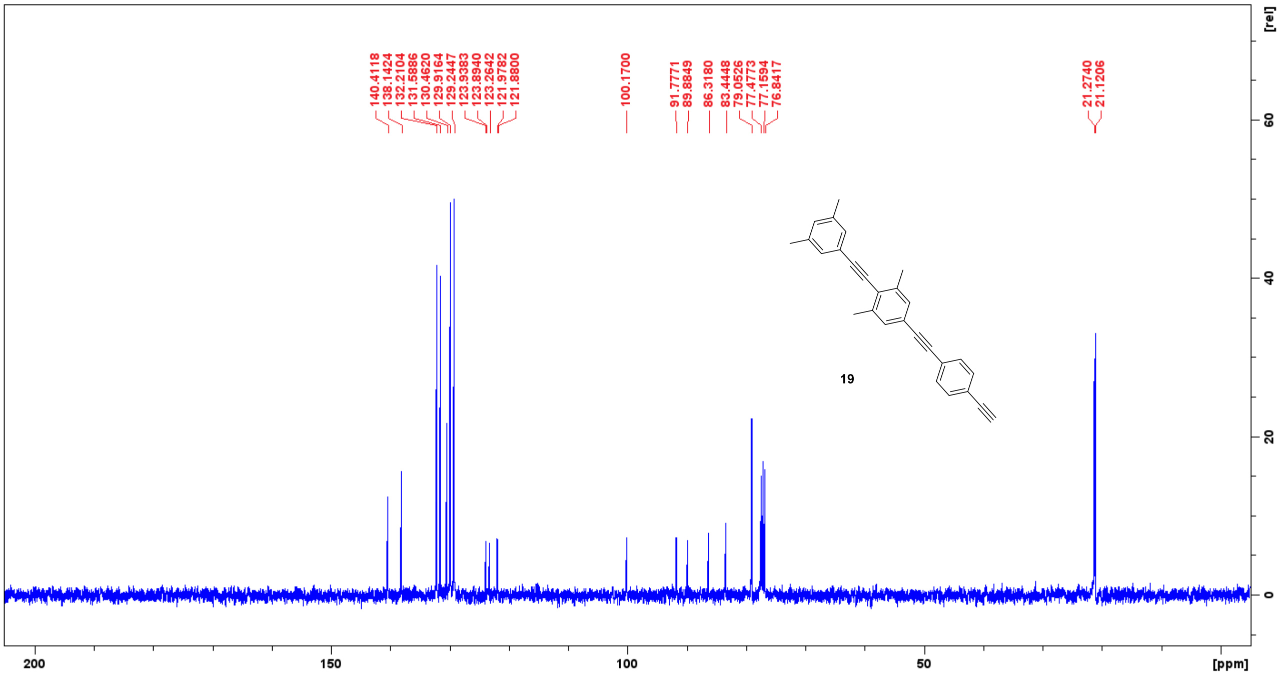

Fig. S44 $^{13}C\{^{1}H\}$ NMR (101 MHz) spectrum of 19 in $CDCl_3$

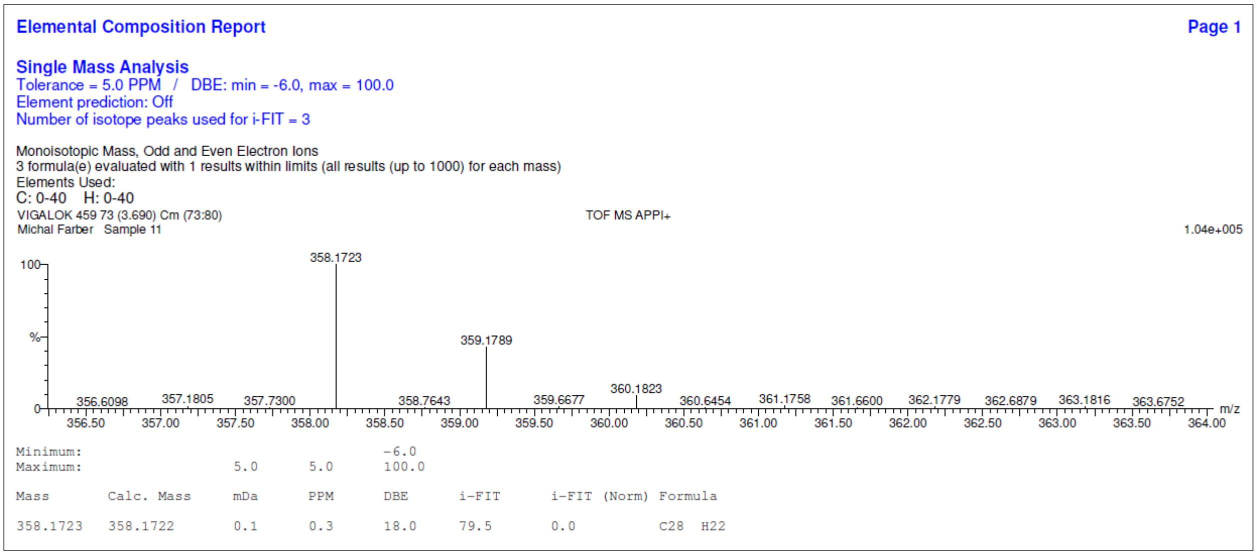

Elemental Composition Report

Page 1

Single Mass Analysis
Tolerance = 5.0 PPM / DBE: min = -6.0, max = 100.0
Element prediction: Off
Number of isotope peaks used for i-FIT = 3

Monoisotopic Mass, Odd and Even Electron Ions
3 formula(e) evaluated with 1 results within limits (all results (up to 1000) for each mass)
Elements Used:
C: 0-40 H: 0-40
VIGALOK 459 73 (3.690) Cm (73:80)
Michal Farber Sample 11

TOF MS APPI+

1.04e+005

Minimum:				-6.0			
Maximum:		5.0	5.0	100.0			
Mass	Calc. Mass	mDa	PPM	DBE	i-FIT	i-FIT (Norm)	Formula
358.1723	358.1722	0.1	0.3	18.0	79.5	0.0	C28 H22

Fig. S45 Mass spectrum of 19

4. Absorption and Emission Spectra

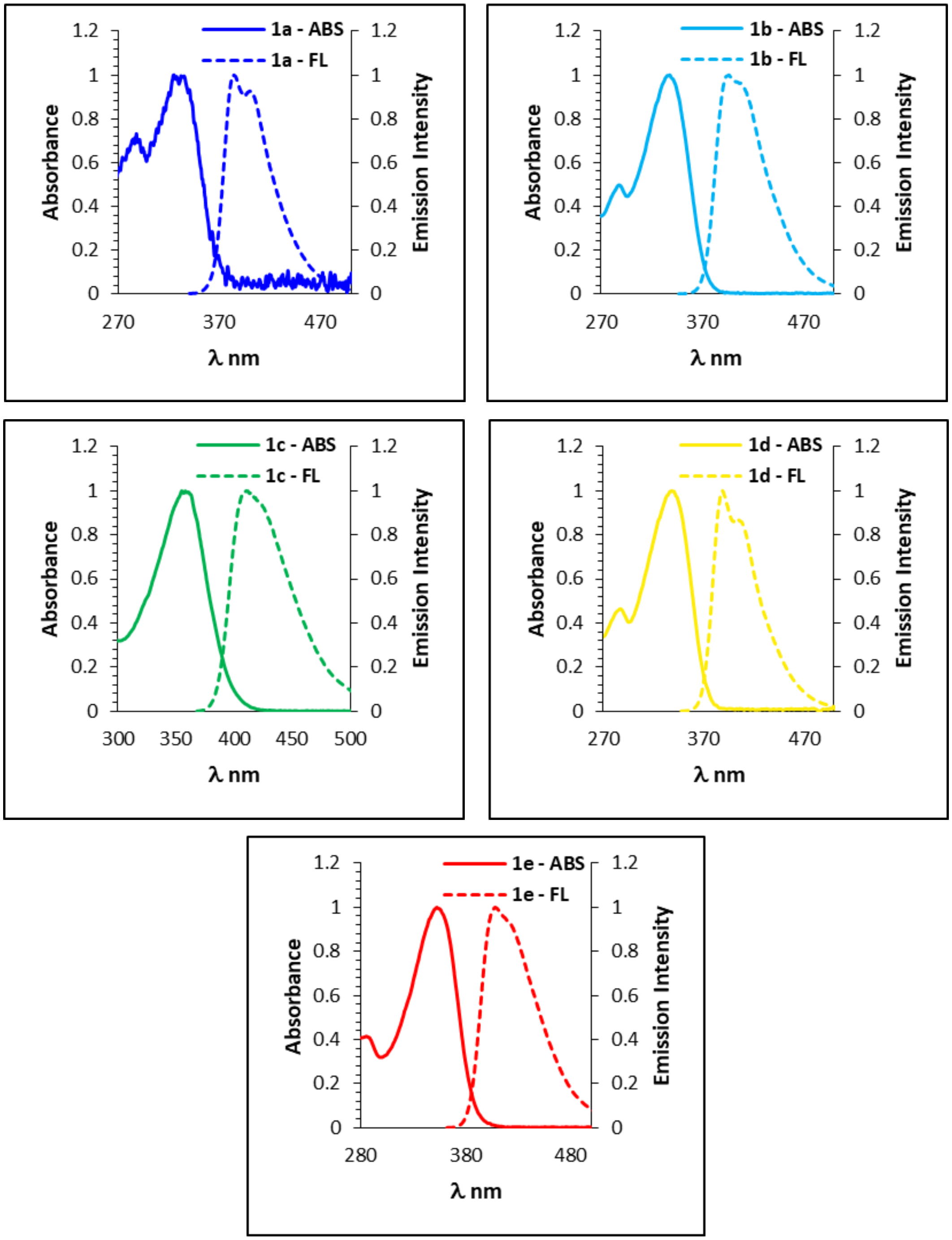

Figure S46: Normalized Absorption and Emission spectra of compounds **1** (10 μM in $CHCl_3$:CH_3CN – 9:1)

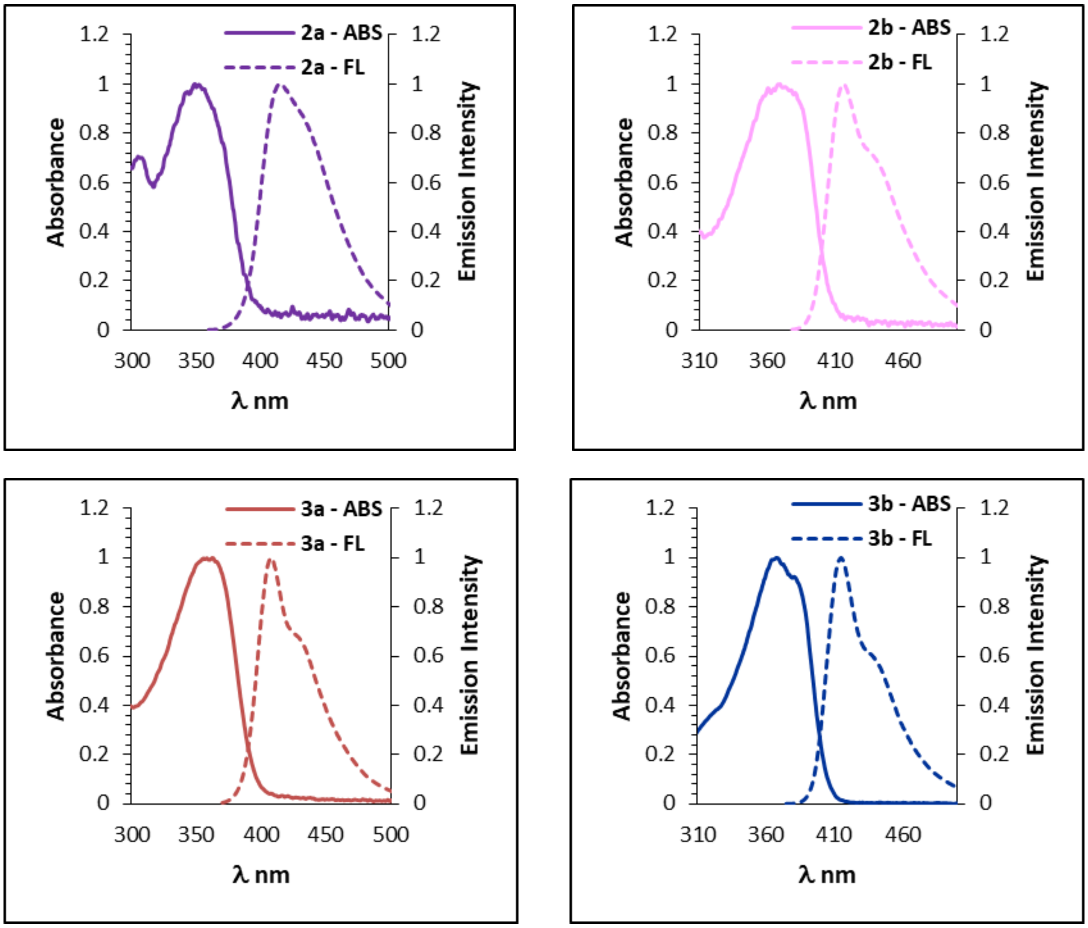

Figure S47: Normalized Absorption and Emission spectra of compounds **2-3** (10 μM in $CHCl_3$: CH_3CN – 9:1)

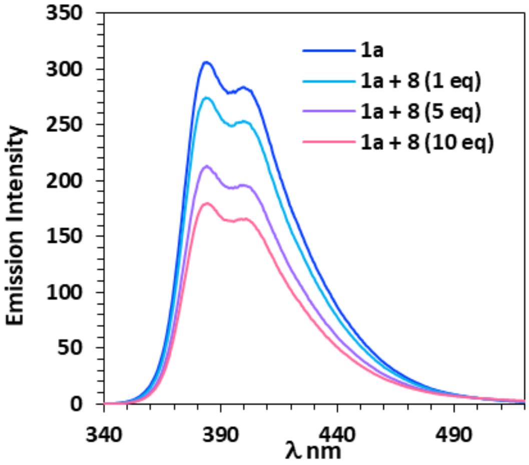
Emission Intensity
350
300
250
200
150
100
50
0
340
390
440
490
λ nm
1a
1a + 8 (1 eq)
1a + 8 (5 eq)
1a + 8 (10 eq)

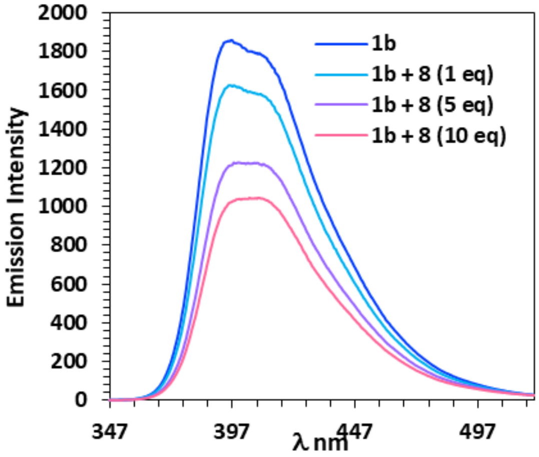
Emission Intensity
2000
1800
1600
1400
1200
1000
800
600
400
200
0
347
397
447
497
λ nm
1b
1b + 8 (1 eq)
1b + 8 (5 eq)
1b + 8 (10 eq)

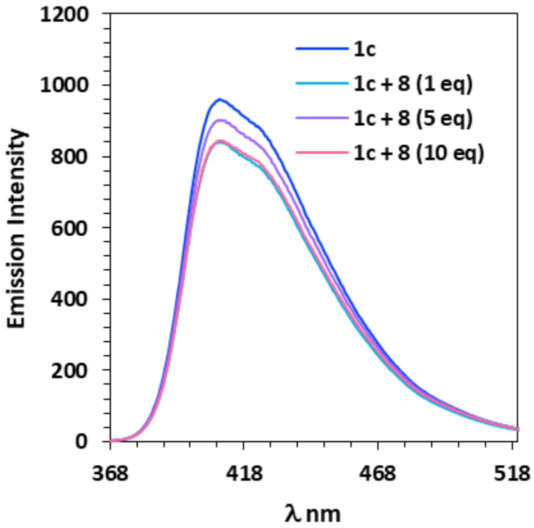
Emission Intensity
1200
1000
800
600
400
200
0
368
418
468
518
λ nm
1c
1c + 8 (1 eq)
1c + 8 (5 eq)
1c + 8 (10 eq)

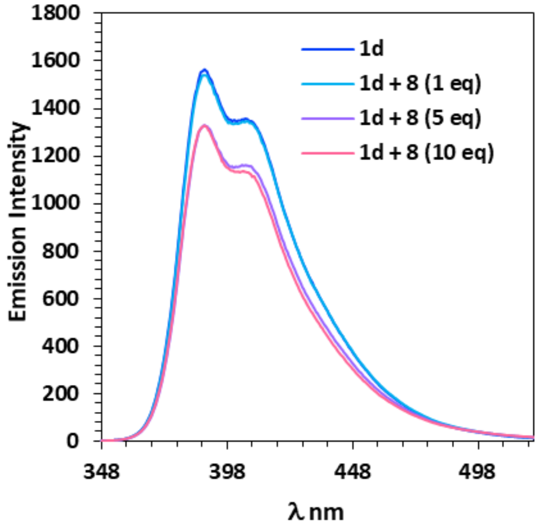
Emission Intensity
1800
1600
1400
1200
1000
800
600
400
200
0
348
398
448
498
λ nm
1d
1d + 8 (1 eq)
1d + 8 (5 eq)
1d + 8 (10 eq)

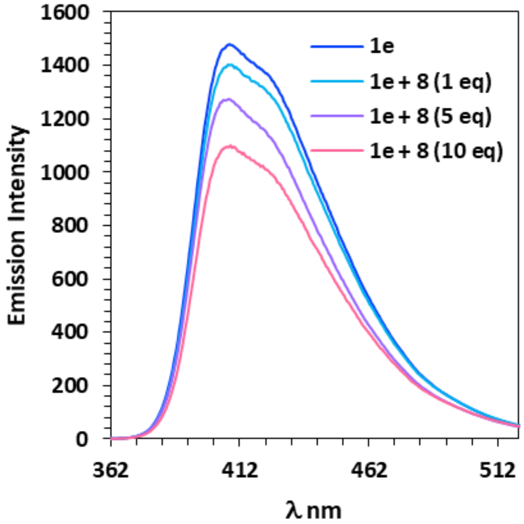
Emission Intensity
1600
1400
1200
1000
800
600
400
200
0
362
412
462
512
λ nm
1e
1e + 8 (1 eq)
1e + 8 (5 eq)
1e + 8 (10 eq)

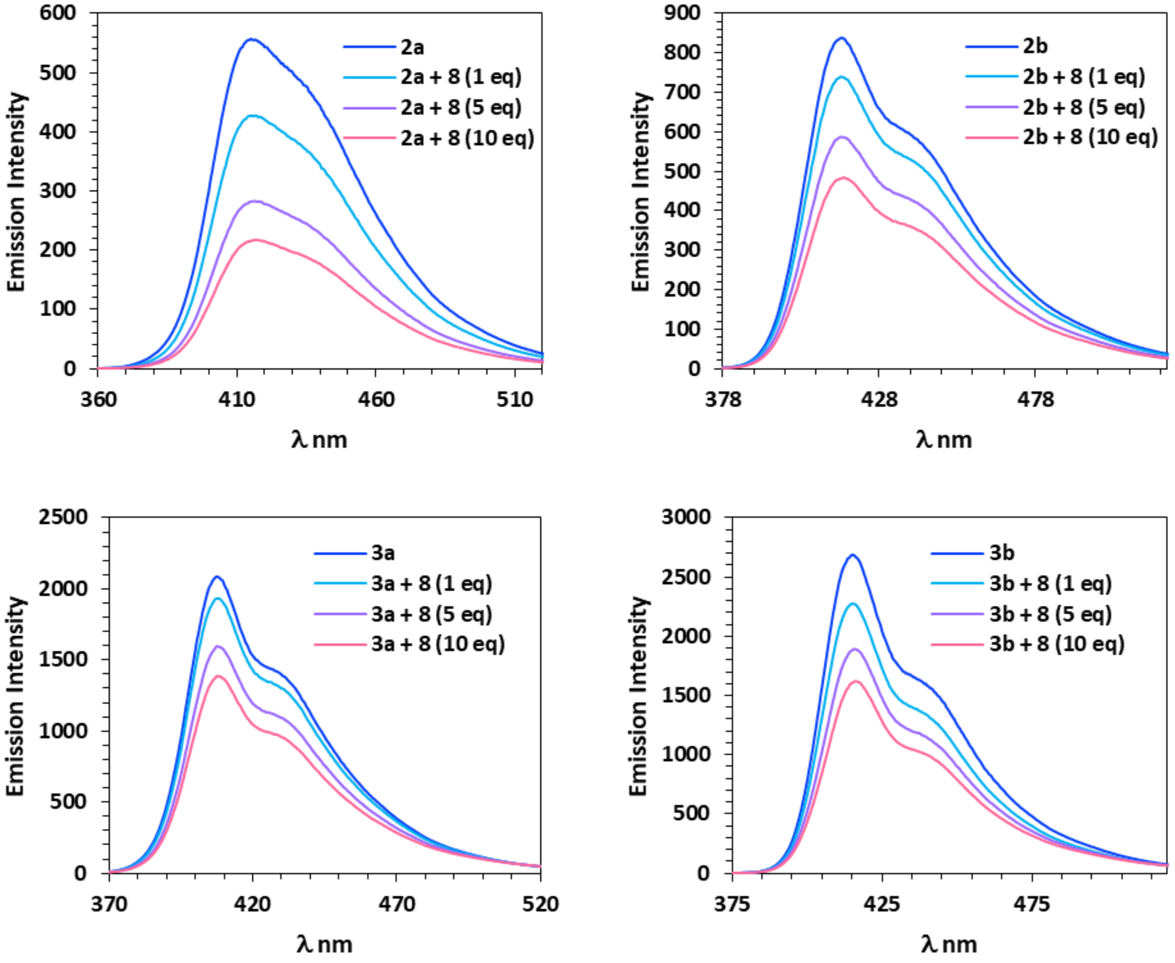

Figure S48: Emission spectra of **1-3** (10 µM in $CHCl_3$: CH_3CN – 9:1) with **8**.

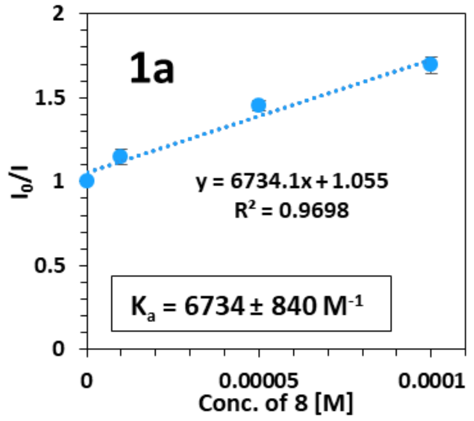
1a
y = 6734.1x + 1.055
R² = 0.9698
Ka = 6734 ± 840 M-1
I0/I
Conc. of 8 [M]

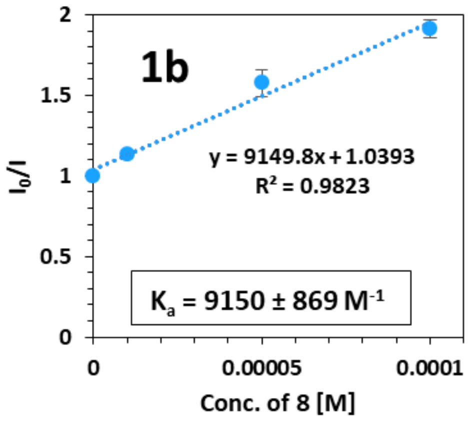
1b
y = 9149.8x + 1.0393
R² = 0.9823
Ka = 9150 ± 869 M-1
I0/I
Conc. of 8 [M]

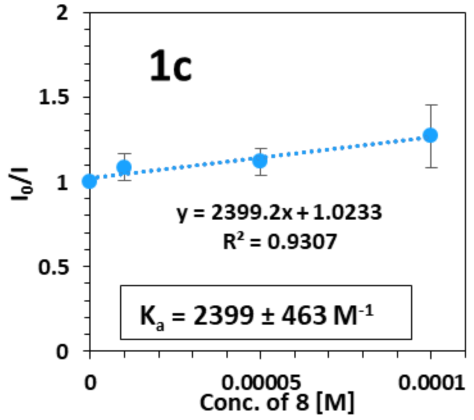
1c
y = 2399.2x + 1.0233
R² = 0.9307
Ka = 2399 ± 463 M-1
I0/I
Conc. of 8 [M]

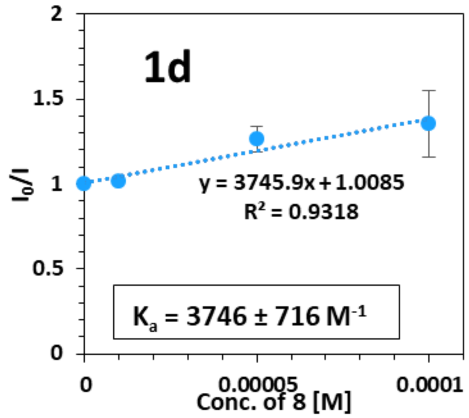
1d
y = 3745.9x + 1.0085
R² = 0.9318
Ka = 3746 ± 716 M-1
I0/I
Conc. of 8 [M]

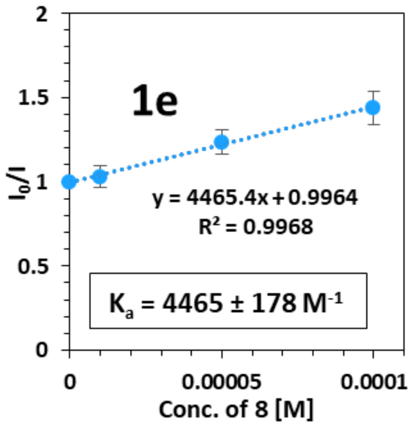
1e
y = 4465.4x + 0.9964
R² = 0.9968
Ka = 4465 ± 178 M-1
I0/I
Conc. of 8 [M]

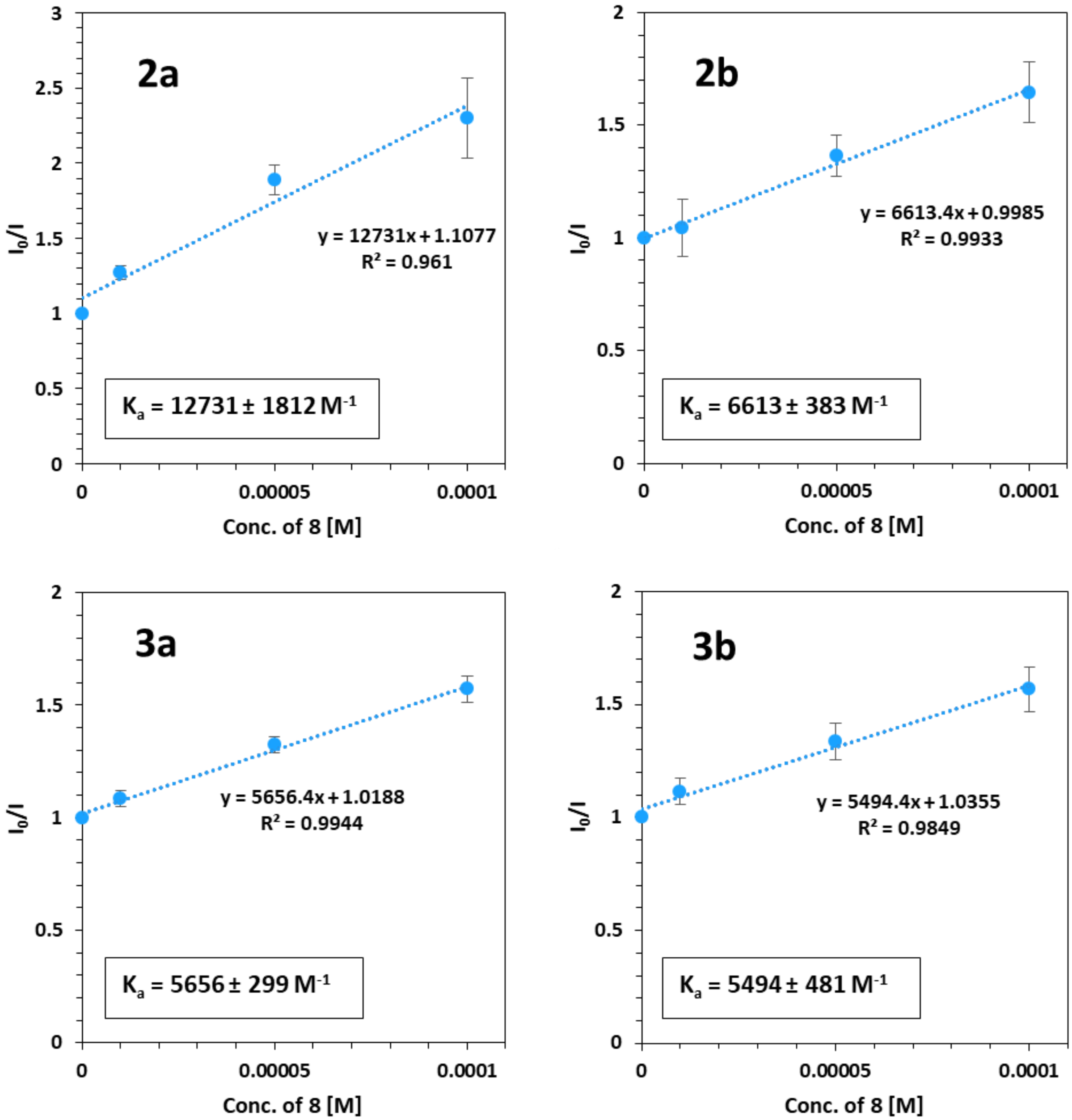

Figure S49: Calculation of the association constants between **1-3** and **8**.

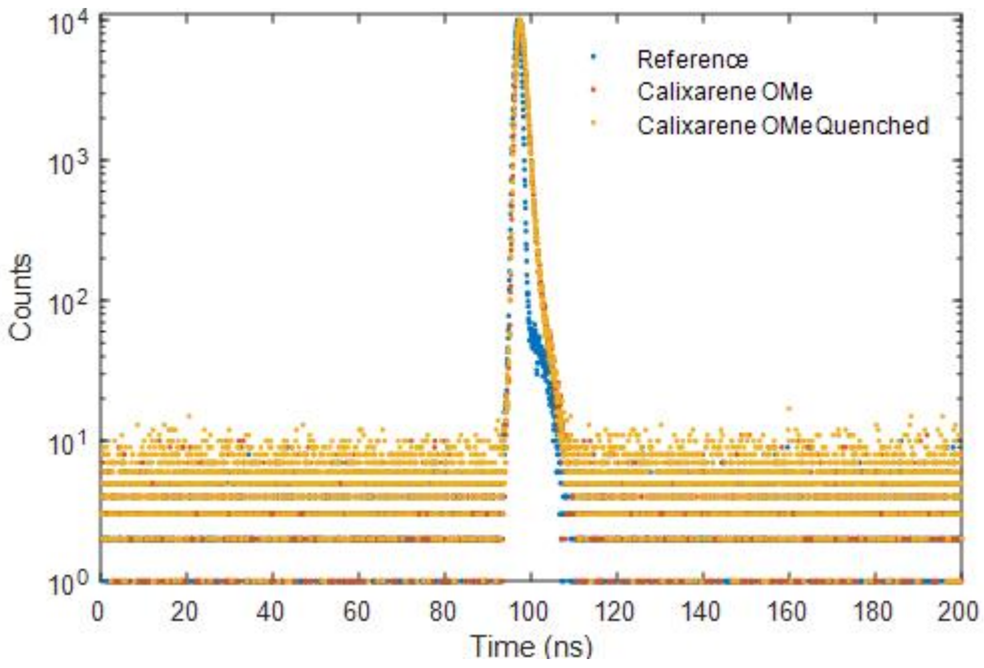

Figure S50: TCSPC data for **1b** ("Calixarene OMe") - semilog plot of the data. 1 MHz 390nm excitation, 405 nm emission.

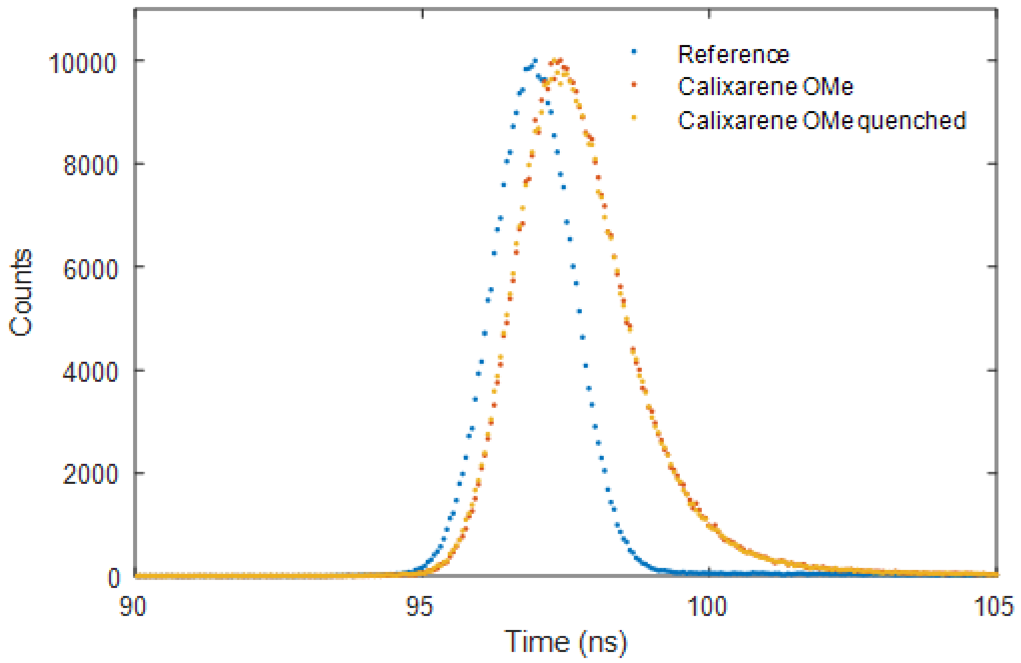

Figure S51: TCSPC data for **1b** ("Calixarene OMe") - plot of counts vs. time.

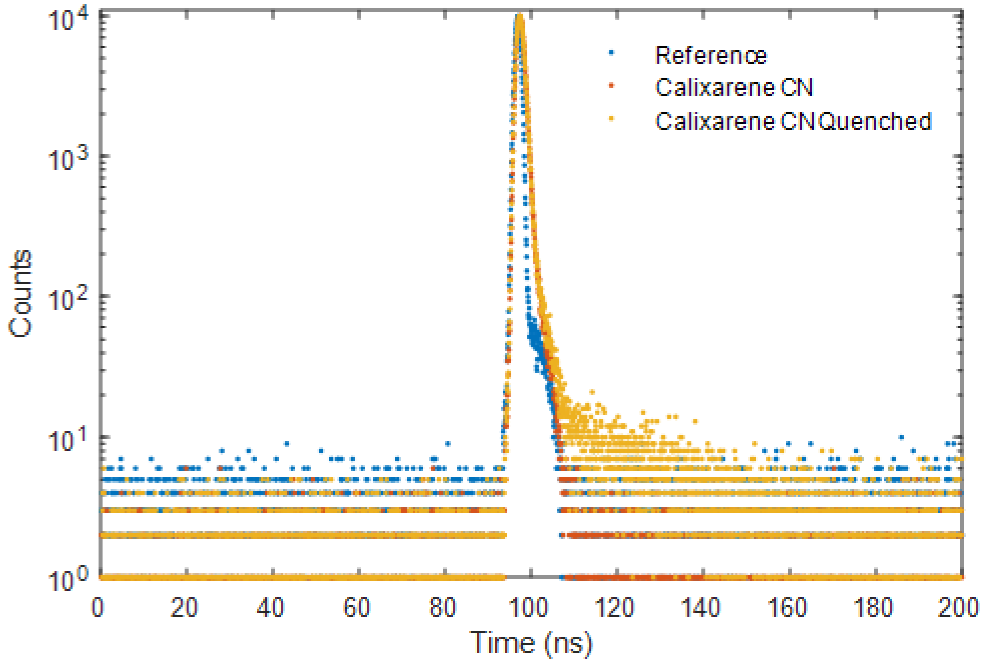

Figure S52: TCSPC data for **1b** ("Calixarene CN") - semilog plot of the data. 1 MHz 390nm excitation, 405 nm emission.

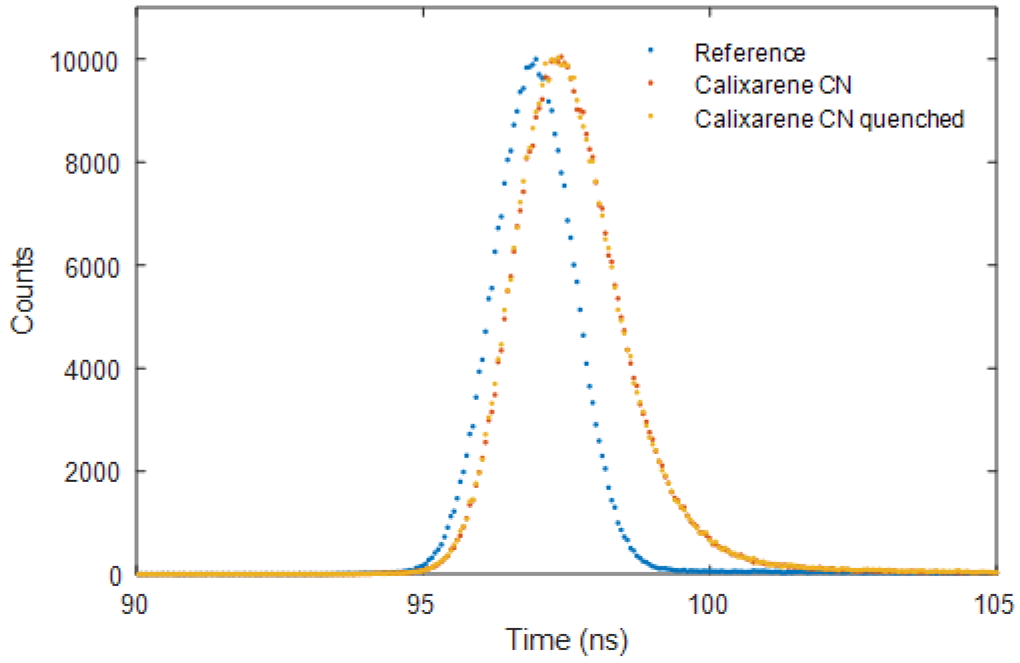

Figure S53: TCSPC data for ***1b*** ("*Calixarene CN*") - plot of counts vs. time.

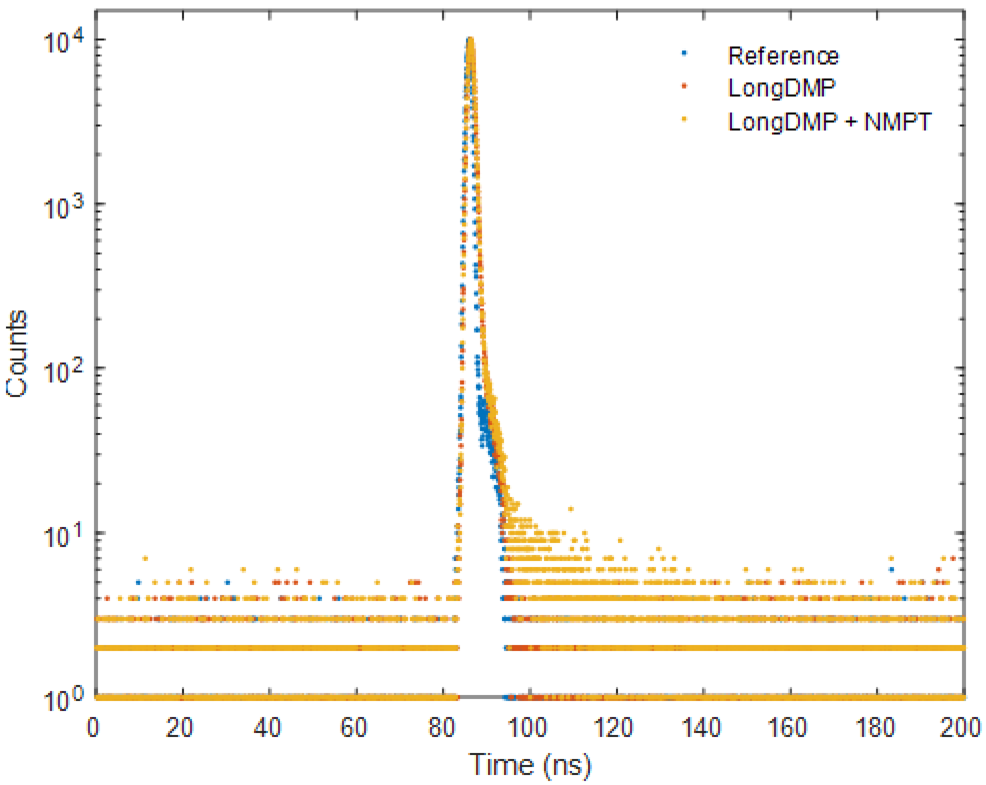

Figure S54: TCSPC data for **3b** ("*long DMP*") - semilog plot of the data. 1 MHz 390nm excitation, 416 nm emission.

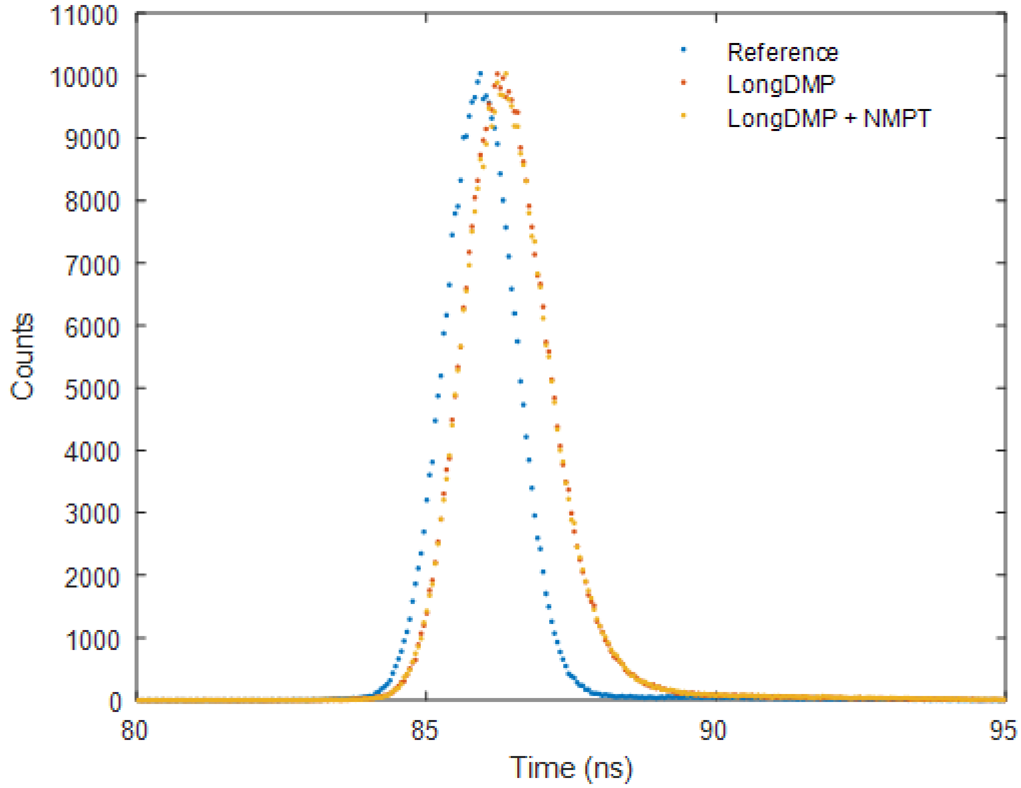

Figure S55: TCSPC data for **3b** ("*long DMP*") - plot of counts vs. time.

5. NMR Binding experiments

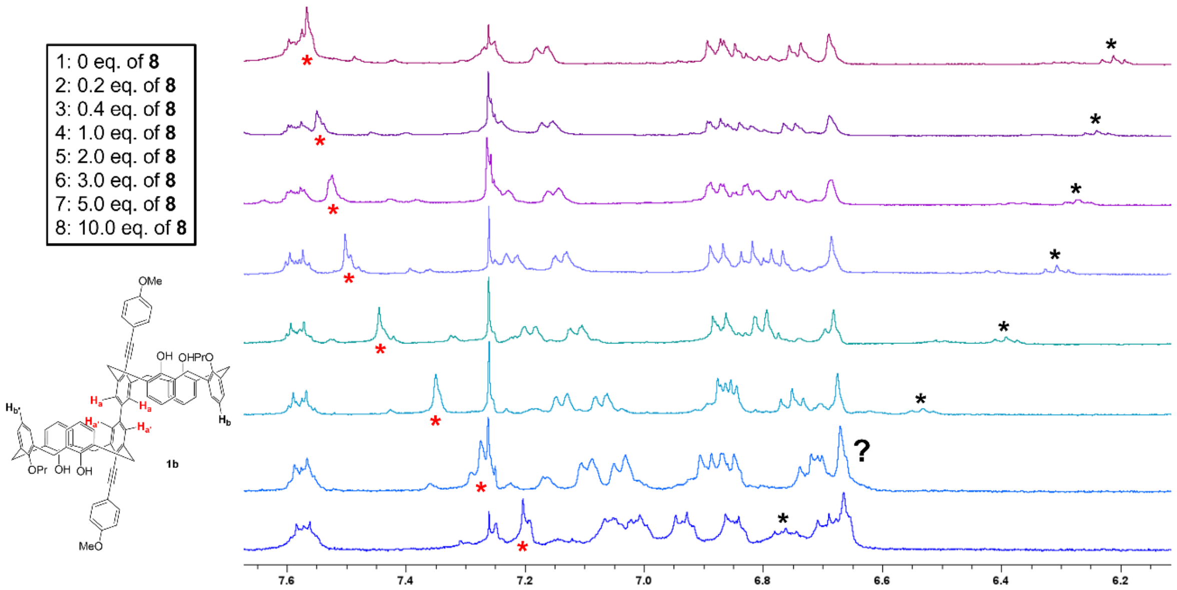

Figure S56: Aromatic region of the ^{1}H NMR spectra (9:1 $CDCl_3$:CD_3CN) of **1b** at various concentrations of **8**.

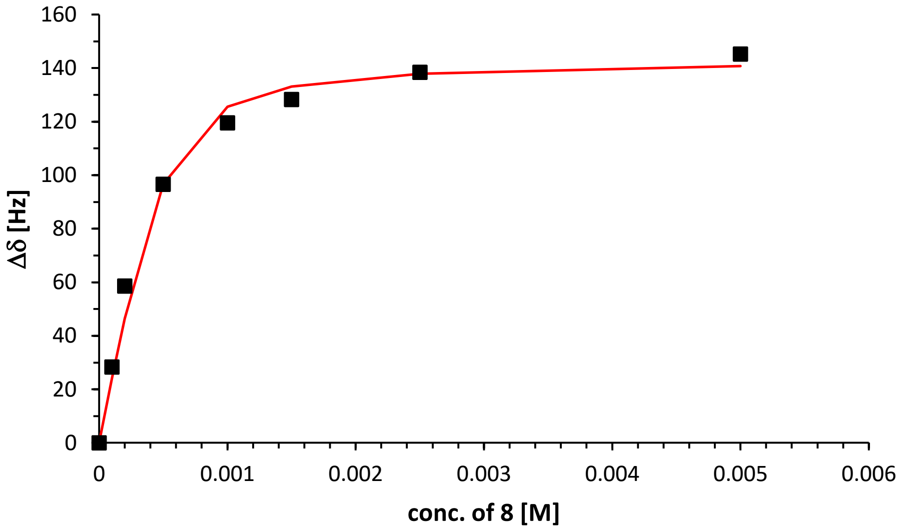

Figure S57: Chemical shift changes of the biphenylene protons of **1b** as a function of added **8**.

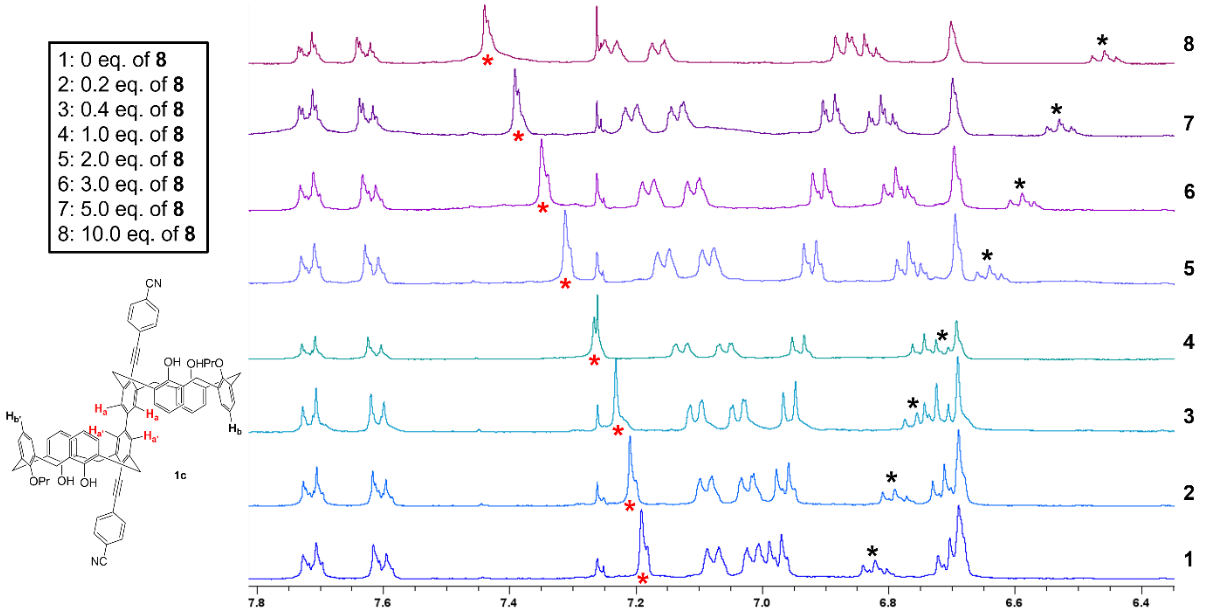

Figure S58: Aromatic region of the ^{1}H NMR spectra (9:1 $CDCl_3$:CD_3CN) of **1c** at various concentrations of **8**.

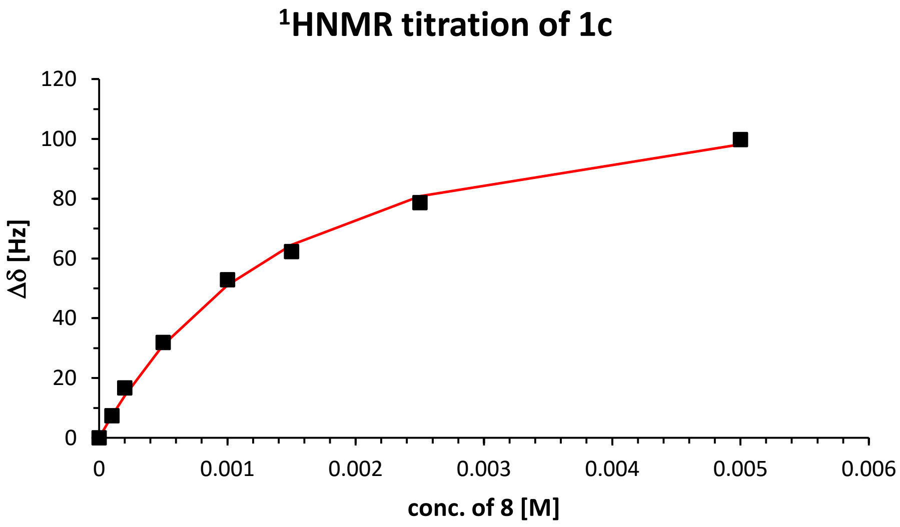

Figure S59: Chemical shift changes of the biphenylene protons of **1c** as a function of added **8**.

6. DFT calculations

DFT calculations were performed using Gaussian 09.2 Geometry optimization of all the molecules were carried out using the B3LYP-D3/6-31G(d,p) basis sets implemented in the Gaussian 09 software.[8] Thermal energy corrections were extracted from the results of frequency analysis performed at the same level of theory. Frequency analysis of all the molecules contained no imaginary frequency showing that these are energy minima.

Cartesian coordinates of the optimized structures:

1b-OMe open:

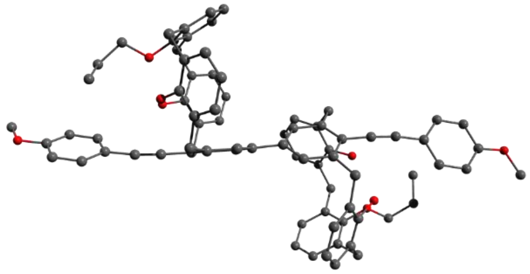

O	-6.41186	1.92796	-0.88425
O	-5.10640	-0.45776	-1.54624
O	-5.40747	1.19115	1.58894
H	-5.81543	1.53115	0.77439
C	-7.81140	2.09692	-1.24426
H	-7.86634	2.25982	-2.32696
H	-8.19216	2.99631	-0.74410
C	-4.03772	3.92029	-2.85837
H	-3.53831	3.88381	-3.82238
C	-3.76122	4.95465	-1.96626
H	-3.05499	5.73229	-2.24061
C	-4.38175	4.98714	-0.71949
H	-4.15109	5.78083	-0.01460
C	-8.57225	0.85026	-0.82460
H	-9.56555	0.90614	-1.28809
H	-8.07423	-0.02366	-1.25929
C	-5.55471	2.97347	-1.26757
C	-5.29497	3.99572	-0.34493
C	-5.92626	4.02187	1.04130
H	-6.84949	3.43456	1.04842
H	-6.21436	5.05268	1.26954
C	-4.93960	2.90453	-2.52506
C	-4.32076	4.42277	2.97093
H	-4.48341	5.48929	2.83457
C	-8.71146	0.67541	0.69021
H	-7.74174	0.57477	1.18117

H	-9.28165	-0.22855	0.91738
H	-9.23317	1.53141	1.13457
C	-4.99606	3.52427	2.13654
C	-4.78338	2.14779	2.32532
C	-3.90971	1.67232	3.32205
C	-3.63657	0.18132	3.41612
H	-3.12425	-0.02765	4.36188
H	-4.57597	-0.37647	3.41593
C	-2.76606	-0.28064	2.25366
C	-1.46985	0.22199	2.15712
H	-1.11369	0.90355	2.92478
C	-0.65039	-0.06010	1.05761
C	-1.16714	-0.87804	0.04544
H	-0.54664	-1.10582	-0.81514
C	-2.45649	-1.39483	0.09362
C	-2.96155	-2.16073	-1.12548
H	-2.27297	-2.98375	-1.34713
H	-3.94240	-2.59094	-0.92005
C	-3.01768	-1.22374	-2.32023
C	-4.07664	-0.30149	-2.42913
C	-4.06611	0.70017	-3.41678
C	-5.17093	1.74169	-3.48254
H	-6.13515	1.26558	-3.28437
H	-5.22213	2.13777	-4.50148
C	-3.46430	3.97286	3.97249
H	-2.95567	4.68285	4.61708
C	-3.26428	2.60040	4.13903
H	-2.58976	2.24078	4.91216
C	-2.99428	0.74409	-4.31580
H	-2.98564	1.51266	-5.08494
C	-1.95803	-0.18293	-4.24693
H	-1.14282	-0.14508	-4.96260
C	-1.97426	-1.15649	-3.24615
H	-1.14857	-1.85879	-3.16739
C	-3.26999	-1.11335	1.22071
C	-4.59192	-1.63467	1.27643
C	-5.73194	-2.05495	1.25359
H	-5.57541	0.37539	-1.36235
O	6.32172	-2.15353	-0.50941
O	5.66183	-0.52717	1.67589
O	4.84937	-0.17529	-1.76891
H	5.37897	-0.91000	-1.41366
C	7.70993	-2.48616	-0.79270
H	8.03537	-3.23424	-0.06068
H	7.76113	-2.94058	-1.79031
C	4.31918	-4.90768	0.87213

H	4.14840	-5.39972	1.82536
C	3.59463	-5.29351	-0.25376
H	2.86881	-6.09770	-0.18154
C	3.79355	-4.64295	-1.46943
H	3.21558	-4.92998	-2.34336
C	8.54046	-1.21650	-0.70424
H	9.59525	-1.51982	-0.69824
H	8.34736	-0.74174	0.26465
C	5.43849	-3.24418	-0.43576
C	4.72275	-3.60359	-1.58567
C	4.88894	-2.87259	-2.91176
H	5.86584	-2.38185	-2.95512
H	4.87850	-3.61379	-3.71683
C	5.25594	-3.87120	0.80449
C	2.70724	-2.16264	-4.00493
H	2.66803	-3.14244	-4.47501
C	8.29200	-0.21725	-1.83756
H	7.25881	0.13423	-1.85537
H	8.92880	0.66207	-1.71438
H	8.51569	-0.67009	-2.81082
C	3.79289	-1.85274	-3.17739
C	3.83584	-0.58142	-2.57888
C	2.81749	0.36567	-2.79963
C	2.84572	1.66889	-2.01906
H	2.14760	2.37773	-2.47803
H	3.84293	2.11268	-2.04547
C	2.42848	1.40221	-0.57802
C	1.14205	0.92446	-0.35809
H	0.46535	0.84587	-1.20323
C	0.70246	0.51709	0.90714
C	1.59722	0.62248	1.97880
H	1.29714	0.25902	2.95760
C	2.89359	1.10535	1.80848
C	3.84017	1.08075	3.00480
H	3.38138	1.62239	3.83957
H	4.77080	1.59075	2.75040
C	4.11122	-0.34975	3.43579
C	4.97105	-1.15913	2.66813
C	5.11622	-2.52952	2.94704
C	5.98070	-3.41502	2.06541
H	6.90505	-2.88928	1.81042
H	6.27476	-4.30161	2.63557
C	1.69390	-1.23687	-4.24216
H	0.86433	-1.49175	-4.89445
C	1.75605	0.02115	-3.63709
H	0.95593	0.73816	-3.80076

C	4.41622	-3.07081	4.03184
H	4.53211	-4.12846	4.25620
C	3.59493	-2.27758	4.82768
H	3.06978	-2.70883	5.67418
C	3.44424	-0.92456	4.51868
H	2.78608	-0.30192	5.11952
C	3.32046	1.51472	0.51724
C	4.64688	1.98985	0.32462
C	5.80254	2.33567	0.17743
H	5.93150	-1.12446	0.95624
C	7.20077	2.57019	0.07791
C	8.07357	1.95959	0.99749
C	7.75900	3.36976	-0.94136
C	9.45381	2.13286	0.90887
H	7.65175	1.32448	1.76996
C	9.13095	3.54497	-1.03608
H	7.10102	3.84439	-1.66185
C	9.99013	2.92899	-0.11250
H	10.09526	1.64410	1.63240
C	-7.08523	-2.45001	1.07407
C	-7.60134	-2.58839	-0.22771
C	-7.95358	-2.67183	2.16341
C	-8.93551	-2.93031	-0.44315
H	-6.94417	-2.39595	-1.06987
C	-9.28174	-3.01051	1.95605
H	-7.57271	-2.56503	3.17377
C	-9.78459	-3.14133	0.65187
H	-9.29994	-3.02338	-1.45917
H	9.56856	4.15597	-1.81849
H	-9.95746	-3.17866	2.78796
O	11.32219	3.16784	-0.29535
O	-11.10579	-3.47457	0.55983
C	12.24027	2.56471	0.60301
H	12.18334	1.46839	0.56617
H	13.23258	2.88293	0.27990
H	12.07415	2.89546	1.63675
C	-11.67724	-3.59710	-0.73326
H	-12.72657	-3.85176	-0.57695
H	-11.61429	-2.65542	-1.29513
H	-11.19635	-4.39312	-1.31701

Sum of electronic and zero-point Energies= -3691.436076
Sum of electronic and thermal Energies= -3691.361333
Sum of electronic and thermal Enthalpies= -3691.360389
Sum of electronic and thermal Free Energies= -3691.547684

1b-OMe closed:

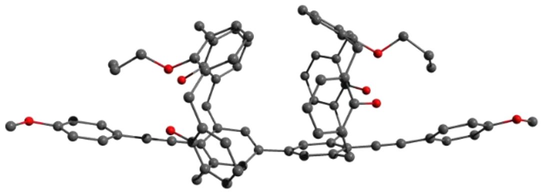

O	4.99209	0.84117	1.89382
O	4.53015	-1.59693	0.58626
O	4.83164	1.80397	-0.69059
H	4.90362	1.54212	0.24420
C	6.17782	0.93531	2.73686
H	5.98280	0.36921	3.65474
H	6.32268	1.98829	3.01014
C	1.87627	0.66580	3.83516
H	1.29389	-0.06333	4.38916
C	1.42472	1.97801	3.72095
H	0.49980	2.27828	4.20082
C	2.15373	2.90767	2.98488
H	1.78851	3.92469	2.87800
C	7.37011	0.37705	1.97855
H	8.17979	0.24311	2.70713
H	7.11403	-0.62378	1.61388
C	3.79546	1.22785	2.52184
C	3.35792	2.55075	2.36987
C	4.11984	3.56968	1.53365
H	5.18108	3.30698	1.48991
H	4.06029	4.54006	2.03621
C	3.07072	0.26010	3.23107
C	2.68471	4.75075	-0.20017
H	2.38478	5.44818	0.57844
C	7.85223	1.25473	0.82094
H	7.08836	1.37367	0.05059
H	8.72556	0.80472	0.34275
H	8.13299	2.25191	1.18081
C	3.57821	3.72189	0.12051
C	3.96274	2.82945	-0.89541
C	3.48257	2.96771	-2.21171
C	3.83786	1.90776	-3.24212
H	3.63186	2.29823	-4.24489
H	4.89990	1.66060	-3.18804
C	2.99743	0.66074	-2.99507
C	1.61850	0.77228	-3.13950
H	1.21066	1.70924	-3.50559
C	0.74587	-0.26098	-2.78075

C	1.30556	-1.44601	-2.28742
H	0.64767	-2.22960	-1.92697
C	2.67881	-1.61241	-2.14188
C	3.17511	-2.88907	-1.46839
H	2.78129	-3.75819	-2.00704
H	4.26377	-2.93600	-1.50990
C	2.68460	-2.93217	-0.03113
C	3.34754	-2.17695	0.95491
C	2.82251	-2.06409	2.25453
C	3.50579	-1.19861	3.30140
H	4.59027	-1.28529	3.19496
H	3.25707	-1.58424	4.29509
C	2.19969	4.90718	-1.49671
H	1.52041	5.72082	-1.73195
C	2.60738	4.01724	-2.49402
H	2.23338	4.13228	-3.50830
C	1.63021	-2.73226	2.55563
H	1.22333	-2.64974	3.55965
C	0.97227	-3.50147	1.60111
H	0.04117	-4.00398	1.84140
C	1.50560	-3.59637	0.31558
H	0.97990	-4.17417	-0.44021
C	3.54911	-0.55331	-2.51202
C	4.95377	-0.70780	-2.35663
C	6.14447	-0.86528	-2.17107
H	4.69345	-0.76011	1.05633
O	-5.00526	-0.40575	2.01354
O	-4.99207	-1.85891	-0.37418
O	-4.36474	1.64542	0.27560
H	-4.59201	0.98347	0.95130
C	-6.21038	-0.44952	2.83246
H	-6.25271	-1.43252	3.31542
H	-6.12073	0.31227	3.61716
C	-2.21488	-2.30688	3.46591
H	-1.87227	-3.33460	3.54304
C	-1.46916	-1.27754	4.03503
H	-0.55693	-1.50774	4.57477
C	-1.89052	0.04343	3.91203
H	-1.29622	0.84559	4.33711
C	-7.41985	-0.21025	1.94447
H	-8.30681	-0.48757	2.52847
H	-7.37498	-0.90675	1.09987
C	-3.81674	-0.69895	2.70412
C	-3.07444	0.36275	3.23864
C	-3.49023	1.81860	3.06799
H	-4.57425	1.89087	2.93671

H	-3.25394	2.35383	3.99301
C	-3.40638	-2.03704	2.78546
C	-1.65335	3.30506	2.12763
H	-1.28626	3.42602	3.14343
C	-7.56049	1.22701	1.43644
H	-6.71260	1.52787	0.81872
H	-8.45857	1.32581	0.82205
H	-7.63899	1.92916	2.27507
C	-2.79051	2.51744	1.91106
C	-3.26311	2.37782	0.59478
C	-2.62565	3.01952	-0.48384
C	-3.11849	2.74972	-1.89549
H	-2.72188	3.52020	-2.56591
H	-4.20841	2.79850	-1.93635
C	-2.63943	1.37931	-2.35885
C	-1.26842	1.18388	-2.48529
H	-0.60557	2.01395	-2.26222
C	-0.71882	-0.06895	-2.78423
C	-1.59949	-1.14324	-2.95559
H	-1.19836	-2.12832	-3.17149
C	-2.97670	-1.00163	-2.81940
C	-3.83194	-2.26406	-2.86577
H	-3.61606	-2.81682	-3.78685
H	-4.89059	-2.00215	-2.87350
C	-3.50047	-3.14301	-1.67037
C	-4.03654	-2.83375	-0.40528
C	-3.62519	-3.52965	0.74605
C	-4.17689	-3.16757	2.11569
H	-5.23596	-2.91185	2.02429
H	-4.12377	-4.05011	2.76059
C	-1.00209	3.93970	1.07351
H	-0.11182	4.53381	1.25176
C	-1.49489	3.79479	-0.22483
H	-0.97807	4.27571	-1.05136
C	-2.67772	-4.55112	0.61044
H	-2.36313	-5.09742	1.49645
C	-2.15870	-4.89154	-0.63610
H	-1.44116	-5.70115	-0.72796
C	-2.57449	-4.18398	-1.76581
H	-2.16510	-4.43428	-2.74117
C	-3.51819	0.28118	-2.54241
C	-4.92529	0.44167	-2.41994
C	-6.13003	0.53042	-2.28782
H	-5.00789	-1.37982	0.47372
C	-7.52465	0.52180	-2.01649
C	-8.13891	-0.65893	-1.55999

C	-8.32184	1.67734	-2.15701
C	-9.49690	-0.69272	-1.24688
H	-7.52693	-1.54598	-1.43014
C	-9.67267	1.65059	-1.84757
C	-10.27185	0.46642	-1.38945
H	-9.93516	-1.61828	-0.89336
C	7.49414	-1.08744	-1.78602
C	7.77618	-1.97596	-0.72279
C	8.56809	-0.42493	-2.40126
C	9.07846	-2.18160	-0.29772
H	6.94778	-2.47280	-0.22809
C	9.88166	-0.62725	-1.97765
C	10.14255	-1.50880	-0.92027
H	9.30449	-2.85702	0.52083
H	10.68501	-0.09640	-2.47378
H	8.36765	0.26391	-3.21537
O	11.38402	-1.78255	-0.42225
C	12.50004	-1.12386	-1.00140
H	12.42712	-0.03314	-0.89583
H	13.37614	-1.47893	-0.45665
H	12.61210	-1.37264	-2.06494
H	-7.86320	2.59715	-2.50462
H	-10.29242	2.53506	-1.95019
O	-11.60634	0.55002	-1.11315
C	-12.26828	-0.61356	-0.64217
H	-13.30982	-0.32719	-0.48877
H	-12.22324	-1.43126	-1.37355
H	-11.84671	-0.96322	0.30993

Sum of electronic and zero-point Energies= -3691.444720
Sum of electronic and thermal Energies= -3691.370000
Sum of electronic and thermal Enthalpies= -3691.369056
Sum of electronic and thermal Free Energies= -3691.554835

1c-CN open:

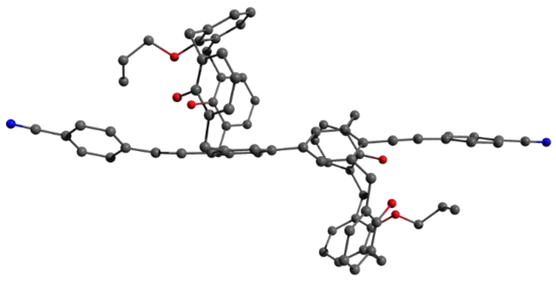

O	-6.44144	1.86994	-0.84222

O	-5.06845	-0.44544	-1.59536
O	-5.42327	1.01941	1.59511
H	-5.83981	1.39618	0.80101
C	-7.84050	2.04529	-1.20531
H	-7.88773	2.26795	-2.27744
H	-8.23442	2.91139	-0.65909
C	-4.07878	4.00115	-2.68084
H	-3.57002	4.02512	-3.64032
C	-3.82860	4.99073	-1.73201
H	-3.13383	5.79397	-1.95730
C	-4.46173	4.94663	-0.49190
H	-4.25143	5.70591	0.25590
C	-8.59268	0.77064	-0.86309
H	-9.58669	0.84762	-1.32090
H	-8.08942	-0.07150	-1.35203
C	-5.59553	2.94699	-1.15873
C	-5.36211	3.92229	-0.18026
C	-6.00708	3.86409	1.19868
H	-6.91841	3.25943	1.16704
H	-6.31810	4.87549	1.47747
C	-4.96752	2.95524	-2.41153
C	-4.43102	4.19667	3.16413
H	-4.61973	5.26451	3.08462
C	-8.72916	0.50501	0.63901
H	-7.75851	0.38145	1.12350
H	-9.29887	-0.41102	0.81330
H	-9.25401	1.33050	1.13382
C	-5.07583	3.32908	2.27493
C	-4.83089	1.94988	2.39104
C	-3.95542	1.44341	3.37051
C	-3.64749	-0.04355	3.39265
H	-3.13578	-0.28756	4.33010
H	-4.57329	-0.62261	3.35933
C	-2.75860	-0.42658	2.21644
C	-1.47130	0.10273	2.15580
H	-1.13197	0.74969	2.95988
C	-0.64090	-0.10955	1.04850
C	-1.13366	-0.88280	-0.00994
H	-0.50277	-1.05571	-0.87558
C	-2.41420	-1.42227	0.00007
C	-2.89812	-2.13205	-1.26003
H	-2.19250	-2.92876	-1.51954
H	-3.87096	-2.59265	-1.08495
C	-2.96655	-1.13498	-2.40443
C	-4.04253	-0.22898	-2.47097
C	-4.05014	0.81772	-3.41069

C	-5.17254	1.84203	-3.43166
H	-6.13079	1.34127	-3.26855
H	-5.22209	2.29034	-4.42861
C	-3.57236	3.71463	4.14881
H	-3.08850	4.40079	4.83665
C	-3.33970	2.34088	4.24300
H	-2.66481	1.95599	5.00350
C	-2.97724	0.92414	-4.30295
H	-2.98255	1.72690	-5.03618
C	-1.92195	0.01663	-4.27419
H	-1.10645	0.10480	-4.98495
C	-1.92100	-1.00333	-3.32108
H	-1.08273	-1.69347	-3.27382
C	-3.23731	-1.21167	1.13556
C	-4.54878	-1.75686	1.15325
C	-5.67652	-2.20770	1.10179
H	-5.57195	0.36455	-1.39671
O	6.34910	-2.05879	-0.57198
O	5.64885	-0.54533	1.68022
O	4.85882	-0.02595	-1.72197
H	5.39802	-0.77589	-1.41521
C	7.73714	-2.38332	-0.87051
H	8.05781	-3.17043	-0.17871
H	7.78767	-2.78425	-1.89066
C	4.33291	-4.87310	0.66141
H	4.15664	-5.41137	1.58826
C	3.61372	-5.20137	-0.48599
H	2.88652	-6.00698	-0.45760
C	3.82129	-4.49286	-1.66739
H	3.24876	-4.73638	-2.55792
C	8.57379	-1.12496	-0.71433
H	9.62710	-1.43049	-0.74259
H	8.39608	-0.71201	0.28549
C	5.46292	-3.14995	-0.55708
C	4.75318	-3.45122	-1.72709
C	4.92889	-2.65566	-3.01420
H	5.90451	-2.16065	-3.02521
H	4.92782	-3.35586	-3.85512
C	5.27294	-3.83756	0.64924
C	2.75839	-1.89610	-4.09502
H	2.73097	-2.84848	-4.61883
C	8.31188	-0.05727	-1.78026
H	7.28251	0.30543	-1.75391
H	8.96476	0.80476	-1.62318
H	8.50772	-0.45312	-2.78359
C	3.83165	-1.62726	-3.23781

C	3.85982	-0.39051	-2.57010
C	2.83972	0.56239	-2.75368
C	2.85237	1.82320	-1.90620
H	2.15226	2.55043	-2.33168
H	3.84620	2.27531	-1.90257
C	2.42627	1.47769	-0.48517
C	1.14459	0.97439	-0.29986
H	0.47436	0.93177	-1.15249
C	0.70385	0.49503	0.93964
C	1.58792	0.55351	2.02385
H	1.28473	0.13547	2.97944
C	2.88007	1.05740	1.88930
C	3.81856	0.98079	3.08916
H	3.34824	1.47592	3.94579
H	4.74629	1.51189	2.86992
C	4.09980	-0.46711	3.44913
C	4.96663	-1.23051	2.64387
C	5.12499	-2.61112	2.85741
C	5.99459	-3.44722	1.93357
H	6.91908	-2.90820	1.70857
H	6.28913	-4.36149	2.45764
C	1.74255	-0.96403	-4.29459
H	0.92348	-1.18721	-4.97114
C	1.79017	0.25949	-3.62190
H	0.98921	0.98133	-3.75820
C	4.42980	-3.20927	3.91484
H	4.55595	-4.27504	4.08884
C	3.60046	-2.46291	4.74705
H	3.08030	-2.93893	5.57222
C	3.43706	-1.09843	4.50289
H	2.77487	-0.51079	5.13377
C	3.30738	1.53814	0.62293
C	4.63040	2.02981	0.46452
C	5.78282	2.39966	0.35057
H	5.94583	-1.11163	0.94589
C	7.17998	2.63011	0.28492
C	8.02956	1.95735	1.19245
C	7.75169	3.48201	-0.68187
C	9.40404	2.12787	1.12904
H	7.58463	1.28244	1.91590
C	9.12738	3.65193	-0.74820
H	7.10390	3.99782	-1.38217
C	9.96660	2.97492	0.15548
H	10.05406	1.60434	1.82227
C	-7.01496	-2.61460	0.87421
C	-7.49635	-2.68007	-0.45308

C	-7.89148	-2.92301	1.93435
C	-8.81177	-3.03640	-0.70809
H	-6.82389	-2.41601	-1.26248
C	-9.20887	-3.27792	1.68091
H	-7.52700	-2.87114	2.95444
C	-9.68193	-3.33604	0.35716
H	-9.18063	-3.07851	-1.72757
H	9.56382	4.30377	-1.49745
H	-9.88173	-3.50882	2.49986
C	11.38679	3.13820	0.08004
C	-11.04409	-3.68876	0.09382
N	12.54213	3.26444	0.01622
N	-12.15286	-3.96922	-0.12254

Sum of electronic and zero-point Energies= -3646.935531
Sum of electronic and thermal Energies= -3646.862154
Sum of electronic and thermal Enthalpies= -3646.861210
Sum of electronic and thermal Free Energies= -3647.046421

1c-CN closed:

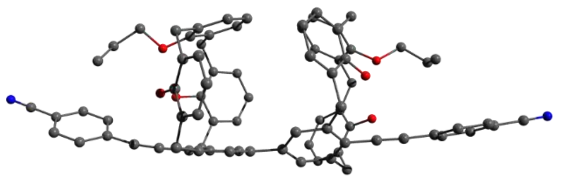

O	4.97396	0.54547	1.97520
O	4.50376	-1.63208	0.22989
O	4.82611	1.83694	-0.49821
H	4.91083	1.49968	0.40999
C	6.07315	0.54548	2.92585
H	5.82504	-0.14804	3.73781
H	6.16601	1.55219	3.35280
C	1.58096	0.05634	3.28868
H	0.92732	-0.75894	3.57693
C	1.08424	1.35409	3.21947
H	0.04558	1.55239	3.45358
C	1.90971	2.39519	2.80467
H	1.51294	3.40236	2.72244
C	7.33685	0.12332	2.19467
H	8.11324	-0.04055	2.95227
H	7.15448	-0.84751	1.71927
C	3.69937	0.83369	2.49301
C	3.23734	2.15616	2.43765

C	4.09790	3.30224	1.92179
H	5.15610	3.02497	1.94262
H	3.98791	4.15295	2.60137
C	2.90061	-0.23098	2.92930
C	2.93486	4.88884	0.31139
H	2.65052	5.49389	1.16898
C	7.82569	1.13376	1.15309
H	7.09071	1.29239	0.36141
H	8.74325	0.77753	0.67828
H	8.03861	2.10288	1.61906
C	3.70472	3.73922	0.52199
C	4.07124	2.96481	-0.59168
C	3.66923	3.32300	-1.89210
C	3.96808	2.37580	-3.03955
H	3.75826	2.88129	-3.98871
H	5.02279	2.09212	-3.03971
C	3.09568	1.13273	-2.91935
C	1.71375	1.29420	-2.96010
H	1.31132	2.28590	-3.14307
C	0.83786	0.22695	-2.72935
C	1.38978	-1.03573	-2.47677
H	0.73043	-1.85922	-2.22363
C	2.76293	-1.24761	-2.43014
C	3.25957	-2.63243	-2.02412
H	2.87270	-3.37561	-2.73018
H	4.34872	-2.66851	-2.06935
C	2.75979	-2.96055	-0.62952
C	3.36035	-2.33817	0.48032
C	2.80218	-2.45708	1.76422
C	3.38246	-1.67656	2.92874
H	4.47452	-1.72312	2.90460
H	3.07140	-2.14861	3.86600
C	2.54318	5.27074	-0.96975
H	1.95790	6.17303	-1.11732
C	2.91225	4.48250	-2.06235
H	2.60037	4.76400	-3.06503
C	1.64984	-3.23516	1.92623
H	1.21267	-3.32395	2.91768
C	1.06138	-3.88578	0.84600
H	0.16106	-4.47578	0.98018
C	1.61948	-3.74007	-0.42452
H	1.14161	-4.21299	-1.27858
C	3.63525	-0.15172	-2.65544
C	5.03754	-0.33778	-2.52686
C	6.22280	-0.52504	-2.33139
H	4.67436	-0.93398	0.88663

O	-5.08183	-0.81439	1.95461
O	-4.93959	-1.79789	-0.65565
O	-4.30609	1.40859	0.56117
H	-4.61856	0.71017	1.16211
C	-6.30540	-1.02717	2.71783
H	-6.34098	-2.08213	3.01217
H	-6.25311	-0.41919	3.62953
C	-2.24483	-2.83244	3.12454
H	-1.85651	-3.84175	3.02407
C	-1.56532	-1.89677	3.90155
H	-0.66317	-2.18898	4.43001
C	-2.04181	-0.59119	4.00687
H	-1.50059	0.13824	4.60227
C	-7.49180	-0.64440	1.85094
H	-8.39524	-0.98592	2.37118
H	-7.43813	-1.21234	0.91530
C	-3.89857	-1.17896	2.61817
C	-3.21807	-0.20062	3.35649
C	-3.70370	1.24288	3.43504
H	-4.78536	1.28527	3.27196
H	-3.53328	1.60771	4.45241
C	-3.43107	-2.49184	2.46747
C	-2.02667	3.08137	2.91111
H	-1.78542	3.10778	3.97129
C	-7.59399	0.85460	1.55560
H	-6.73664	1.21825	0.98532
H	-8.48930	1.06641	0.96648
H	-7.65570	1.43137	2.48584
C	-3.00553	2.18546	2.46338
C	-3.33650	2.19392	1.09624
C	-2.69096	3.05806	0.18949
C	-3.07048	3.00804	-1.28152
H	-2.64817	3.88549	-1.78314
H	-4.15741	3.06100	-1.38368
C	-2.55488	1.74696	-1.96202
C	-1.18015	1.58732	-2.10958
H	-0.52266	2.36711	-1.73630
C	-0.62577	0.41903	-2.65260
C	-1.49817	-0.60931	-3.02736
H	-1.08885	-1.52909	-3.43181
C	-2.87576	-0.50857	-2.87154
C	-3.71893	-1.74760	-3.15261
H	-3.47959	-2.13102	-4.15029
H	-4.78000	-1.49724	-3.13715
C	-3.39788	-2.81571	-2.11883
C	-3.96481	-2.73863	-0.83200

C	-3.56172	-3.61695	0.19083
C	-4.14612	-3.51231	1.59058
H	-5.21206	-3.27921	1.52177
H	-4.07306	-4.49111	2.07436
C	-1.37416	3.94151	2.03064
H	-0.62455	4.63531	2.39787
C	-1.70489	3.91737	0.67382
H	-1.19248	4.57959	-0.01924
C	-2.59776	-4.58857	-0.10178
H	-2.29329	-5.27839	0.68164
C	-2.04995	-4.70135	-1.37689
H	-1.31999	-5.47530	-1.59287
C	-2.45389	-3.81274	-2.37444
H	-2.02298	-3.88572	-3.36970
C	-3.42232	0.69931	-2.36773
C	-4.83221	0.83159	-2.26153
C	-6.04376	0.88418	-2.17616
H	-4.99936	-1.49018	0.26643
C	-7.44108	0.78929	-1.95993
C	-8.01795	-0.48202	-1.73951
C	-8.26897	1.93003	-1.92414
C	-9.37466	-0.60465	-1.48126
H	-7.37209	-1.35372	-1.74579
C	-9.62688	1.80901	-1.66507
C	-10.19187	0.54071	-1.43784
H	-9.81293	-1.58116	-1.30382
C	7.54234	-0.79972	-1.89118
C	7.74365	-1.81028	-0.92337
C	8.65414	-0.07045	-2.35899
C	9.01469	-2.07590	-0.43755
H	6.87949	-2.34750	-0.54711
C	9.92663	-0.33365	-1.87154
C	10.11849	-1.33774	-0.90500
H	9.16542	-2.84669	0.31102
H	10.77905	0.23382	-2.22949
H	8.50365	0.70772	-3.09923
H	-7.83027	2.90758	-2.09167
H	-10.25940	2.68969	-1.63240
C	-11.58934	0.41526	-1.15393
C	11.42798	-1.60150	-0.39021
N	-12.72390	0.31147	-0.91562
N	12.49046	-1.81364	0.03521

Sum of electronic and zero-point Energies= -3646.943704
Sum of electronic and thermal Energies= -3646.870637
Sum of electronic and thermal Enthalpies= -3646.869693

Sum of electronic and thermal Free Energies= -3647.051426

[Me-Pyr]$^+$:

C	0.19169	-1.17798	-0.00997
C	0.19085	1.17851	-0.00996
C	-1.19332	1.20426	0.00288
C	-1.89949	-0.00051	0.00863
C	-1.19233	-1.20485	0.00287
H	0.79583	-2.07709	-0.01423
H	0.79402	2.07823	-0.01420
H	-1.70270	2.16072	0.00535
H	-2.98437	-0.00096	0.01641
H	-1.70101	-2.16167	0.00533
C	2.34601	0.00004	0.01576
H	2.67938	-0.01570	1.05537
H	2.71560	-0.88026	-0.50892
H	2.71583	0.89581	-0.48165
N	0.86099	0.00058	-0.01781

Sum of electronic and zero-point Energies= -287.864193
Sum of electronic and thermal Energies= -287.858057
Sum of electronic and thermal Enthalpies= -287.857113
Sum of electronic and thermal Free Energies= -287.894887

1b-OMe closed with [Me-Pyr]$^+$:

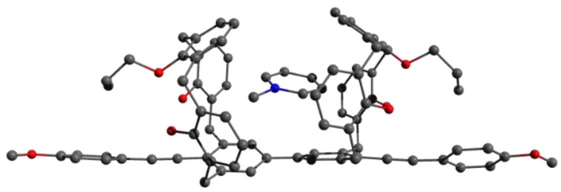

O	5.78005	1.30659	1.45310
O	4.72303	-1.24062	1.25427
O	5.12177	0.99518	-1.20629
H	5.53471	1.23781	-0.35925
C	7.05932	1.56540	2.11273
H	6.91675	1.43190	3.19098
H	7.33196	2.61140	1.92931
C	2.79630	2.53715	3.20959
H	2.12952	2.22960	4.01032
C	2.56712	3.73093	2.52852
H	1.72792	4.36154	2.80530
C	3.40963	4.11346	1.48649
H	3.21800	5.03304	0.94070
C	8.09034	0.60352	1.55107

H	8.99078	0.70198	2.16945
H	7.73108	-0.42273	1.68843
C	4.72066	2.13916	1.83815
C	4.49984	3.31893	1.11268
C	5.34636	3.70067	-0.09669
H	6.32390	3.21222	-0.05246
H	5.54002	4.77663	-0.06418
C	3.88167	1.71754	2.88048
C	4.01949	4.37274	-2.15826
H	4.07965	5.40203	-1.81429
C	8.43670	0.84608	0.07969
H	7.57882	0.68141	-0.57573
H	9.21588	0.15327	-0.24575
H	8.80060	1.86861	-0.07480
C	4.65310	3.37032	-1.41160
C	4.57519	2.04310	-1.87497
C	3.88997	1.71439	-3.06348
C	3.85470	0.27425	-3.55789
H	3.51523	0.27845	-4.59865
H	4.86979	-0.13115	-3.54604
C	2.94745	-0.63993	-2.74368
C	1.56185	-0.53636	-2.88599
H	1.16836	0.16639	-3.61516
C	0.67605	-1.32153	-2.13123
C	1.24087	-2.18090	-1.17414
H	0.58978	-2.72781	-0.50076
C	2.61175	-2.32240	-0.99934
C	3.10408	-3.19257	0.15379
H	2.60810	-4.16753	0.11207
H	4.17797	-3.35847	0.06543
C	2.77022	-2.52264	1.47713
C	3.58539	-1.47481	1.95790
C	3.21916	-0.73533	3.09981
C	4.09479	0.39846	3.61259
H	5.14521	0.09896	3.56077
H	3.87464	0.55444	4.67262
C	3.31967	4.06844	-3.32435
H	2.83896	4.85671	-3.89449
C	3.26285	2.74408	-3.76694
H	2.73302	2.50471	-4.68505
C	2.02428	-1.06256	3.75425
H	1.74069	-0.49835	4.63915
C	1.21544	-2.10575	3.30678
H	0.29612	-2.35644	3.82611
C	1.59913	-2.83070	2.17490
H	0.96339	-3.63276	1.81161

C	3.49123	-1.58578	-1.83154
C	4.88978	-1.81232	-1.74071
C	6.07844	-2.02693	-1.60657
H	5.13605	-0.37855	1.44823
O	-5.72538	0.63689	1.86624
O	-5.14131	-1.83157	0.69009
O	-4.76958	1.19826	-0.63071
H	-5.24827	1.18263	0.21659
C	-7.00734	0.87218	2.52782
H	-7.01315	0.30601	3.46578
H	-7.07714	1.93967	2.76927
C	-2.81168	0.44025	4.09378
H	-2.32649	-0.27522	4.75167
C	-2.25307	1.70336	3.90273
H	-1.34272	1.97835	4.42677
C	-2.85963	2.61289	3.03731
H	-2.41889	3.59302	2.87752
C	-8.11313	0.43013	1.58642
H	-9.05291	0.47697	2.15002
H	-7.95795	-0.62316	1.32659
C	-4.58447	1.00901	2.58618
C	-4.04725	2.28774	2.37077
C	-4.69413	3.28775	1.42023
H	-5.74852	3.03730	1.27028
H	-4.68038	4.27370	1.89342
C	-3.97925	0.05987	3.42257
C	-3.23859	4.52013	-0.26201
H	-3.17326	5.33857	0.45017
C	-8.22133	1.27263	0.31255
H	-7.32517	1.18993	-0.30625
H	-9.05834	0.93111	-0.30035
H	-8.38196	2.33016	0.55294
C	-3.99439	3.38895	0.07347
C	-4.08402	2.34183	-0.86480
C	-3.43129	2.41527	-2.11468
C	-3.56402	1.27417	-3.10903
H	-3.14323	1.59867	-4.06641
H	-4.62542	1.06893	-3.27280
C	-2.87822	-0.01476	-2.67326
C	-1.48380	-0.07668	-2.64207
H	-0.92105	0.81314	-2.91169
C	-0.80042	-1.23813	-2.24025
C	-1.58158	-2.32882	-1.82741
H	-1.09247	-3.24392	-1.51341
C	-2.96936	-2.28478	-1.76786
C	-3.69231	-3.42820	-1.06602

H	-3.34393	-4.38589	-1.46636
H	-4.76615	-3.36148	-1.24107
C	-3.38343	-3.37104	0.42472
C	-4.09467	-2.48604	1.26300
C	-3.74150	-2.33058	2.61909
C	-4.49547	-1.36866	3.52806
H	-5.56449	-1.41131	3.30367
H	-4.38338	-1.70454	4.56294
C	-2.59294	4.61617	-1.49313
H	-2.02997	5.50910	-1.74652
C	-2.69231	3.56224	-2.40651
H	-2.19672	3.63477	-3.37089
C	-2.66817	-3.07675	3.12227
H	-2.40467	-2.97480	4.17222
C	-1.96804	-3.97002	2.31519
H	-1.16413	-4.56856	2.73170
C	-2.33258	-4.10936	0.97424
H	-1.79429	-4.80773	0.33899
C	-3.64191	-1.13664	-2.25272
C	-5.06112	-1.12691	-2.30097
C	-6.27610	-1.15113	-2.28869
H	-5.40059	-1.02844	1.17538
C	-7.68779	-1.19477	-2.13569
C	-8.25503	-1.99294	-1.12536
C	-8.54964	-0.42840	-2.94901
C	-9.63297	-2.02840	-0.92269
H	-7.59632	-2.57293	-0.48661
C	-9.92037	-0.45914	-2.75335
C	-10.47547	-1.25921	-1.73954
H	-10.03764	-2.65447	-0.13695
C	7.45288	-2.23885	-1.31762
C	7.83722	-2.66953	-0.02685
C	8.45847	-2.00282	-2.26915
C	9.17250	-2.84986	0.29091
H	7.06706	-2.84060	0.71853
C	9.80446	-2.18489	-1.95709
C	10.16966	-2.61093	-0.67149
H	9.47923	-3.18039	1.27756
H	10.55363	-1.99636	-2.71586
H	8.17963	-1.67051	-3.26373
O	11.44589	-2.82131	-0.25645
C	12.50562	-2.62268	-1.18623
H	12.54732	-1.58230	-1.53318
H	13.42378	-2.85960	-0.64810
H	12.41078	-3.28927	-2.05236
H	-8.12792	0.19190	-3.73298

H	-10.59230	0.12390	-3.37378
O	-11.82957	-1.21648	-1.63553
C	-12.46234	-2.02202	-0.64837
H	-13.53212	-1.83973	-0.75384
H	-12.25914	-3.08844	-0.80746
H	-12.14596	-1.74131	0.36472
C	0.91315	0.29893	0.63881
C	-0.76158	1.86005	0.08694
C	0.17978	2.69726	-0.48064
C	1.51616	2.30437	-0.51643
C	1.88754	1.09257	0.06468
H	1.12443	-0.65473	1.09513
H	-1.81380	2.09820	0.11546
H	-0.15571	3.63136	-0.90999
H	2.25358	2.93343	-0.99236
H	2.90917	0.73774	0.04507
C	-1.40721	-0.27427	1.11244
H	-1.45954	-1.11753	0.42462
H	-1.13078	-0.61960	2.10599
H	-2.37211	0.22381	1.14317
N	-0.38686	0.67651	0.62124

Sum of electronic and zero-point Energies= -3979.404866
Sum of electronic and thermal Energies= -3979.323516
Sum of electronic and thermal Enthalpies= -3979.322572
Sum of electronic and thermal Free Energies= -3979.520833

1c-CN closed with [Me-Pyr]$^+$:

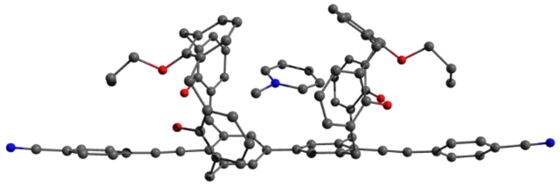

O	5.78499	1.17977	1.47242
O	4.73706	-1.34980	1.11447
O	5.11002	1.02924	-1.20112
H	5.53119	1.22109	-0.34493
C	7.06324	1.41020	2.14647
H	6.92451	1.20420	3.21360
H	7.32314	2.46907	2.03205
C	2.80133	2.30212	3.30090
H	2.13613	1.94823	4.08362
C	2.57448	3.53660	2.69588
H	1.73805	4.15135	3.01355
C	3.41637	3.98114	1.67817

H	3.22648	4.93357	1.19136
C	8.10520	0.50069	1.52200
H	9.00652	0.57257	2.14236
H	7.75924	-0.53681	1.59595
C	4.72355	1.98640	1.90520
C	4.50408	3.20879	1.25406
C	5.34831	3.66117	0.06790
H	6.32384	3.16701	0.07747
H	5.54706	4.73219	0.16511
C	3.88490	1.50251	2.92026
C	4.01322	4.46190	-1.94180
H	4.07965	5.46831	-1.53691
C	8.44296	0.84226	0.06812
H	7.58942	0.69409	-0.59744
H	9.24582	0.19834	-0.29748
H	8.77670	1.88216	-0.02360
C	4.64705	3.41406	-1.26083
C	4.56196	2.11789	-1.80346
C	3.87057	1.86455	-3.00643
C	3.83477	0.45954	-3.59309
H	3.48779	0.52886	-4.62891
H	4.85120	0.05816	-3.61580
C	2.93599	-0.50749	-2.83359
C	1.54920	-0.39937	-2.95625
H	1.14755	0.34764	-3.63486
C	0.67369	-1.23499	-2.24471
C	1.24714	-2.15454	-1.35152
H	0.60325	-2.74555	-0.70997
C	2.61971	-2.30079	-1.19524
C	3.12249	-3.23714	-0.10037
H	2.63277	-4.21081	-0.20128
H	4.19679	-3.39165	-0.20094
C	2.78710	-2.64939	1.26096
C	3.59936	-1.63022	1.80329
C	3.23097	-0.96256	2.98825
C	4.10088	0.14168	3.57080
H	5.15263	-0.14994	3.50385
H	3.87800	0.23182	4.63776
C	3.30603	4.23123	-3.12024
H	2.82611	5.05436	-3.63937
C	3.24274	2.93689	-3.64267
H	2.70987	2.75684	-4.57240
C	2.03757	-1.33342	3.62155
H	1.75354	-0.82585	4.53982
C	1.23171	-2.35019	3.11209
H	0.31470	-2.63660	3.61654

C	1.61661	-3.00315	1.93813
H	0.98440	-3.78536	1.52799
C	3.48650	-1.50621	-1.98535
C	4.88697	-1.73061	-1.91654
C	6.07532	-1.95821	-1.80797
H	5.14641	-0.50030	1.36593
O	-5.72146	0.51208	1.91316
O	-5.11986	-1.87197	0.59105
O	-4.77723	1.20418	-0.56198
H	-5.24766	1.14040	0.28812
C	-6.99653	0.72031	2.59996
H	-6.99375	0.11086	3.51026
H	-7.05889	1.77540	2.89155
C	-2.79517	0.19921	4.11101
H	-2.30634	-0.54980	4.72747
C	-2.24180	1.47315	3.98937
H	-1.33215	1.72267	4.52712
C	-2.85406	2.42746	3.17763
H	-2.41766	3.41697	3.07328
C	-8.11487	0.32658	1.65290
H	-9.04891	0.35872	2.22634
H	-7.97127	-0.71719	1.35128
C	-4.57525	0.84582	2.64373
C	-4.04229	2.13572	2.49673
C	-4.69320	3.18248	1.60115
H	-5.74768	2.93975	1.44047
H	-4.67917	4.14304	2.12368
C	-3.96475	-0.14622	3.42437
C	-3.23924	4.49982	-0.01493
H	-3.17415	5.27892	0.74003
C	-8.22699	1.22142	0.41556
H	-7.33877	1.15521	-0.21717
H	-9.07839	0.91866	-0.19795
H	-8.37134	2.26991	0.70050
C	-3.99540	3.35262	0.26018
C	-4.08688	2.35751	-0.73295
C	-3.43459	2.49861	-1.97704
C	-3.57012	1.41626	-3.03467
H	-3.15204	1.79455	-3.97311
H	-4.63182	1.22080	-3.20798
C	-2.88145	0.10627	-2.67499
C	-1.48710	0.04128	-2.65681
H	-0.92514	0.94397	-2.88030
C	-0.80440	-1.14352	-2.32912
C	-1.58213	-2.25515	-1.96943
H	-1.09252	-3.18733	-1.71276

C	-2.96927	-2.21227	-1.89319
C	-3.68810	-3.38999	-1.24703
H	-3.34011	-4.32515	-1.69739
H	-4.76289	-3.31817	-1.41352
C	-3.37337	-3.40840	0.24359
C	-4.07981	-2.56681	1.12882
C	-3.72816	-2.48942	2.49194
C	-4.48014	-1.57851	3.45395
H	-5.55004	-1.60895	3.23159
H	-4.36539	-1.97048	4.46846
C	-2.59478	4.66228	-1.23966
H	-2.03345	5.56822	-1.44588
C	-2.69525	3.65915	-2.20804
H	-2.20186	3.78396	-3.16815
C	-2.65736	-3.26608	2.95234
H	-2.39477	-3.22476	4.00655
C	-1.95857	-4.11414	2.09660
H	-1.15638	-4.73707	2.47916
C	-2.32388	-4.17855	0.75042
H	-1.79002	-4.84507	0.07828
C	-3.63987	-1.03753	-2.31116
C	-5.05964	-1.02840	-2.35322
C	-6.27374	-1.07152	-2.34796
H	-5.39299	-1.11240	1.13602
C	-7.67996	-1.14793	-2.16815
C	-8.19750	-1.99432	-1.16330
C	-8.56923	-0.37469	-2.94014
C	-9.56336	-2.05294	-0.92871
H	-7.50677	-2.57714	-0.56313
C	-9.93659	-0.43209	-2.70653
C	-10.44380	-1.26744	-1.69510
H	-9.96038	-2.69882	-0.15287
C	7.44895	-2.17942	-1.52768
C	7.82047	-2.68085	-0.26110
C	8.45368	-1.87811	-2.46828
C	9.15772	-2.86461	0.05808
H	7.04357	-2.89738	0.46445
C	9.79265	-2.06099	-2.15059
C	10.15542	-2.55120	-0.88325
H	9.44284	-3.24519	1.03304
H	10.56513	-1.82475	-2.87426
H	8.17217	-1.49555	-3.44315
H	-8.17560	0.27264	-3.71607
H	-10.61989	0.16725	-3.29828
C	0.91436	0.23595	0.61176
C	-0.76069	1.84534	0.21910

C	0.17472	2.71789	-0.30363
C	1.50673	2.32024	-0.40084
C	1.88160	1.06537	0.07798
H	1.12814	-0.75106	0.99024
H	-1.81001	2.08823	0.29218
H	-0.16210	3.68505	-0.65080
H	2.23894	2.97942	-0.84375
H	2.90095	0.70948	0.01255
C	-1.39786	-0.35794	1.09591
H	-1.45606	-1.16146	0.36250
H	-1.11147	-0.75948	2.06524
H	-2.36270	0.13586	1.16541
N	-0.38312	0.62113	0.65053
C	11.53668	-2.71957	-0.54393
C	-11.85159	-1.31080	-1.43502
N	12.65742	-2.84635	-0.25915
N	-12.99282	-1.33959	-1.21137

Sum of electronic and zero-point Energies= -3934.891894
Sum of electronic and thermal Energies= -3934.811905
Sum of electronic and thermal Enthalpies= -3934.810961
Sum of electronic and thermal Free Energies= -3935.006943

7. Reference:

1 Baheti, A.; Dobrovetsky, R.; Vigalok, A. Fluorophore-Appendant 5,5′-Bicalixarene Scaffolds for Host-Guest Sensing of Nitric Oxide. *Org. Lett.* **2020**, *22*, 9706–9711.

2 Lisbjerg, M.; Jessen, B. M.; Rasmussen, B.; Nielsen, B. E.; Madsen, A. Ø.; Pittelkow, M. Discovery of a Cyclic 6 + 6 Hexamer of D-Biotin and Formaldehyde. *Chem. Sci*. **2014**, *5*, 2647-2650

3 Uetomo, A.; Kozaki, M.; Suzuki, S.; Yamanaka, K. I.; Ito, O.; Okada, K. Efficient Light-Harvesting Antenna with a Multi-Porphyrin Cascade. *J. Am. Chem. Soc.* **2011**, *133*, 13276–13279.

4 Mullaney, B. R.; Partridge, B. E.; Beer, P. D. A Halogen-Bonding Bis-Triazolium Rotaxane for Halide-Selective Anion Recognition. *Chem. - A Eur. J.* **2015**, *21*, 1660–1665.

5 Zhao, W.; Huang, L.; Guan, Y.; Wulff, W. D. Three-Component Asymmetric Catalytic Ugi Reaction - Concinnity from Diversity by Substrate-Mediated Catalyst Assembly. *Angew. Chemie - Int. Ed.* **2014**, *53*, 3436–3441.

6 Molad, A.; Goldberg, I.; Vigalok, A. Tubular Conjugated Polymer for Chemosensory Applications. *J. Am. Chem. Soc.* **2012**, *134*, 7290–7292.

7 Marinelli, D.; Fasano, F.; Najjari, B.; Demitri, N.; Bonifazi, D. Borazino-Doped Polyphenylenes. *J. Am. Chem. Soc.* **2017**, *139*, 5503–5519.

8 Gaussian 09, Revision D.01, M. J. Frisch, G. W. Trucks, H. B. Schlegel, G. E. Scuseria, M. A. Robb, J. R. Cheeseman, G. Scalmani, V. Barone, B. Mennucci, G. A. Petersson, H. Nakatsuji, M. Caricato, X. Li, H. P. Hratchian, A. F. Izmaylov, J. Bloino, G. Zheng, J. L. Sonnengerg, M. Hada, M. Ehara, K. Toyota, R. Fukuda, J. Hasegawa, M. Ishida, T. Nakajima, Y. Honda, O. Kitao, H. Nakai, T. Vreven, J. A. Montgomery Jr, J. E. Peralta, F. Ogliaro, M. Bearpark, J. J. Heyd, E. Brothers, K. N. Kudin, V. N. Staroverov, T. Keith, R. Kobayashi, J. Normand, K. Raghavachari, A. Rendell, J. C. Burant, S. S. Iyengar, J. Tomasi, M. Cossi, N. Rega, N. J. Millam, M. Klene, J. E. Knox, J. B. Cross, V. Bakken, C. Adamo, J. Jaramillo, R. Gomperts, R. E. Stratmann, O. Yazyev, A. J. Austin, R. Cammi, C. Pomelli, J. W. Ochterski, R. L. Martin, K. Morokuma, V. G. Zakrzewski, G. A. Voth, P. Salvador, J. J. Dannenberg, S. Dapprich, A. D. Daniels, Ö. Farkas, J. B. Foresman, J. V. Ortiz, J. Cioslowski and D. J. Fox, Gaussian, Inc., Wallingford CT, 2010.
